# Indirect comparisons of traction table versus standard table in total hip arthroplasty through direct anterior approach: a systematic review and frequentist network meta-analysis

**DOI:** 10.1186/s13018-024-04852-3

**Published:** 2024-06-29

**Authors:** Nikolai Ramadanov, Maximilian Voss, Robert Hable, Robert Prill, Hassan Tarek Hakam, Mikhail Salzmann, Dobromir Dimitrov, Emanuele Diquattro, Marko Ostojic, Aleksandra Królikowska, Roland Becker

**Affiliations:** 1 Center of Orthopaedics and Traumatology, University Hospital Brandenburg/Havel, Brandenburg Medical School Theodor Fontane, Brandenburg an der Havel, Germany; 2 Faculty of Health Science Brandenburg, Brandenburg Medical School Theodor Fontane, Brandenburg an der Havel, Germany; 3 Faculty of Applied Computer Science, Deggendorf Institute of Technology, Deggendorf, Germany; 4 Department of Surgical Diseases, Faculty of Medicine, Medical University of Pleven, Pleven, Bulgaria; 5 Orthopaedics,Traumatology and Prosthetic Surgery and Revisions of Hip and Knee Implants, Rizzoli Orthopaedic Institute, Bologna, Italy; 6 Department of Orthopaedics and Traumatology, University Hospital Mostar, Mostar, Bosnia and Herzegovina; 7 Ergonomics and Biomedical Monitoring Laboratory, Wroclaw Medical University, Wrocław, Poland

**Keywords:** Total hip arthroplasty, Hip replacement, Direct anterior approach, DAA, Traction table, Orthopaedic table, Meta-analysis

## Abstract

**Background:**

It remains unclear whether the use of an orthopaedic traction table (TT) in direct anterior approach (DAA) total hip arthroplasty (THA) results in better outcomes. The aim of this systematic review and network meta-analysis was to compare the THA outcomes through DAA on a standard operating table and the THA outcomes through DAA on a TT.

**Methods:**

PubMed, Epistemonikos, and Google Scholar were searched for relevant randomized controlled trials (RCTs) up to 01 January 2024. An indirect comparison in network meta-analysis was performed to assess treatment effects between DAA on a TT and DAA on a standard table, using fixed-effects and random-effects models estimated with frequentist approach and consistency assumption. Standardized mean differences (SMDs) with 95% confidence intervals (CIs) were estimated for continuous variables and odds ratios (ORs) with 95% CIs were estimated for binary variables.

**Results:**

The systematic review of the literature identified 43 RCTs with a total of 2,258 patients. DAA with TT had a 102.3 mL higher intraoperative blood loss and a 0.6 mmol/L lower Hb 3 days postoperatively compared with DAA without TT (SMD = 102.33, 95% CI 47.62 to 157.04; SMD = − 0.60, 95% CI  − 1.19 to − 0.00). DAA with TT had a 0.15 lower periprosthetic fracture OR compared with DAA without TT (OR 0.15, 95% CI 0.03 to 0.86). There were no further significant differences in surgical, radiological, functional outcomes and in complication rates.

**Conclusion:**

Based on our findings and taking into account the limitations, we recommend that particular attention be paid to the risk of periprosthetic fracture in DAA on a standard operating table and blood loss in DAA with TT. Since numerous other surgical, radiological, functional outcome parameters and other complication rates studied showed no significant difference between DAA on a standard operating table and DAA with TT, no recommendation for a change in surgical technique seems justified.

**Level of evidence:**

Level I evidence, because this is a systematic review and meta-analysis of randomized controlled trials.

**Supplementary Information:**

The online version contains supplementary material available at 10.1186/s13018-024-04852-3.

## Introduction

In present day total hip arthroplasty (THA), the direct anterior approach (DAA) has emerged as the leading technique regarding the short-term outcome of THA [1–10]. Today's modern THA through DAA [11, 12] can be performed with both a standard operating table and an orthopedic traction table [11–14]. Both surgical techniques have numerous proponents with rational arguments for their preferred choice. The main advantage of using a TT in DAA is generally a better view of the surgical site with a relatively short skin incision length [11–14]. There is also no risk of injuring the gluteal muscle during the operation [11–14]. However, this improved view is achieved by temporarily placing the operated leg in a non-physiological position [13–15]. Therefore, the foot of the operated leg must be rotated almost 180° externally in the foot holder and the hip must be fully extended under permanent traction [13–15]. With THA through DAA on a standard operating table, this non-physiological leg positioning is not necessary [11–14]. The leg only has to be lowered onto the operating table intraoperatively and thus the hip joint is simply hyperextended by about 30° [11–14]. In addition, on a standard operating table the leg length discrepancy can be easily checked and the prosthesis can be easily tested for a tendency to dislocation. With the DAA on a TT, this is only possible if the operated leg is removed from the foot holder [13–15].

Given the advantages and disadvantages, it is important to determine patient outcomes with both DAA techniques. Nonetheless, the literature is sparse on meaningful studies on this controversial subject. Therefore, our aim is to perform the first systematic review and network meta-analysis of the THA outcome through DAA on a standard operating table compared with the THA outcome through DAA on a TT, including only randomized controlled trials (RCTs) as a source of primary data.

We formulated the following PICO question: In human participants with a hip condition such as osteoarthritis, dysplasia, and avascular necrosis of the femoral head or femoral neck fracture, is THA through DAA on a TT superior to THA through DAA on a standard operating table in terms of surgical, functional and radiological outcomes, and complications?

## Methods

### Search strategy and data selection

The PRISMA Extension Statement for Reporting of Systematic Reviews Incorporating Network Meta-analyses of Health Care Interventions was strictly adhered to for proper reflection of methodology and presentation of meta-data. [16]. The PRISMA Checklist is provided in the supplement. After registration of the study protocol in PROSPERO [CRD42023446806] on 31 July 2023, PubMed, Epistemonikos, and Google Scholar were searched for relevant records up to 01 January 2024. The exact search string was: (((direct anterior approach) OR (DAA) OR (anterior approach)) AND ((total hip arthroplasty) OR (THA) OR (hip replacement))). A BOOLEAN search strategy was used and adapted to the syntax of the searched databases. The search was limited to studies that were not older than 15 years. No further restrictions to the initial literature search were applied.

A step-by-step screening process was conducted according to PRISMA guidelines [17]. After the identification of relevant records in the initial literature search, all duplicates were removed. In the next step, the titles and abstracts of the identified records were screened. Finally, the full texts of the selected records were screened for eligibility, according to the inclusion criteria. The decision on the inclusion of each study was made by the consensus between two reviewers. In terms of persisting disagreement a third reviewer was involved. The inter-reviewer agreement for the two reviewers was calculated for each stage of the search process and it was reported with a Kappa (κ) statistic.

### Inclusion/exclusion criteria

The following inclusion criteria were applied: (i) types of studies: 2- or 3-arm randomized controlled trials (RCTs); (ii) types of participants: human participants with a hip condition such as osteoarthritis, dysplasia, and avascular necrosis of the femoral head or femoral neck fracture; (iii) types of interventions: THA through DAA on a standard operating table compared with conventional surgical THA approach; THA through DAA on an orthopedic traction table compared with another approach or technique, or with another DAA group; (iv) types of outcome measures: surgical outcome parameters: operation time, incision length, intraoperative blood loss; radiological outcome: acetabular cup inclination angle; functional outcome: pain visual analog scale (VAS), Harris Hip Score (HHS) [18]; serum biomarkers: hemoglobin (Hb); complications such as dislocation, infection, periprosthetic fracture, deep vein thrombosis (DVT)/pulmonary embolism (PE), haematoma, lateral femoral cutaneous nerve (LFCN) palsy, and reoperation.

The following exclusion criteria were applied: (i) bilateral THA; (ii) navigated THA or robotic assisted THA; (iii) unclear use of traction table; (iv) no outcome of interest.

### Data extraction

The following data were independently extracted by two reviewers: author names, publication year and study origin, characteristics of participants, THA indication, follow-up period, operating table usage, patient positioning, relevant outcomes, and relevant additional information for the RCT quality assessment. For serum biomarkers, different units were often used in the included RCTs. Therefore, some values had to be converted in order to standardize the units. If the author group and the hospital where the RCT was conducted were the same, we carefully checked whether the patient cohort was the same or different to avoid overlapping data extraction. The extracted data are provided in the supplement.

### Definition of traction table

The “traction table” is a common orthopedic operating table. In the literature, other terms such as “Hana table”, “fracture table” or “extension table” are used as synonyms for “traction table”. Furthermore, this operating table is often described in more detail with the adjective "orthopedic". This network meta-analysis adhered to the term “traction table” (TT). As an alternative to the TT, the standard operating table is also used in DAA regularly. As positioning the patient on a TT does not necessarily mean that the foot is clamped in the foot holder, the corresponding authors of the included RCTs were strictly contacted if there was any doubt about the reported information on the operating table, as this particular information is crucial to the conduct of this study. Information on all authors contacted by phone or email is reported in Table 1.

**Table 1:** Main characteristics of the RCTs and the patient cohort

RCT	Year of publi-cation	Origin	Patients, N	TT used	Patient positioning	Age, years, SD	Sex, male, %	BMI, kg/m^2^, SD	HHS preoperatively, points , SD
Alvarez-Pinzon et al. [33]	2015	USA	25	Yes	Supine	62.4 ± 10.5	60.0	28.2 ± 4.2	48.0 ± 13.8
Barrett et al. [34]*	2013	USA	43	Yes	Supine	61.4 ± 9.2	67.4	30.7 ± 5.4	NR
Barrett et al. [35]*	2019	USA	43	Yes	Supine	61.4 ± 9.2	67.4	30.7 ± 5.4	NR
Bon et al. [36]	2019	France	50	Yes	Supine	67.3 ± 10.0	42.0	26.5 ± 3.6	54.0 ± 14.9
Brismar et al. [37]	2018	Sweden	50	No	Supine	66.0 ± 4.8	64.0	27.0 ± 1.3	NR
Brun et al. [38]	2021	Norway	84	No	Supine	67.2 ± 8.6	29.8	27.7 ± 3.6	NR
Cheng et al. [39]	2017	Australia	35	Yes	Supine	59.0 ± 3.8	42.9	27.7 ± 1.1	NR
Cooper et al. [40]	2022	USA/Canada	60	No	Supine	64.4 ± 10.2	38.3	32.9 ± 4.3	NR
D'Arrigo et al. [41]	2009	Italy	20	No	NR	64.0 ± 8.0	60.0	37.7 ± 19.0	NR
De Anta-Diaz et al. [42]	2016	Spain	50	No	NR	64.8 ± 10.1	52.0	26.6 ± 3.9	44.4 ± 13.6
Fahs et al. [43]	2018	USA	50	Yes	Supine	68.0 ± 8.0	56.0	27.3 ± 4.2	NR
Fraval et al. [44]	2017	Australia	51	Yes	Supine	60.1 ± 10.1	54.9	28.0 ± 3.5	NR
Fraval et al. [45]	2019	Australia	53	Yes	Supine	63.0 ± 9.4	50.9	27.9 ± 5.4	NR
Goyal et al. [46]	2017	USA	108	No	Supine	60.2 ± 8.9	53.7	28.3 ± 4.7	NR
Guild et al. [47]	2017	USA	110	Yes	Supine	61.2 ± 9.6	53.6	30.0 ± 5.4	41.6 ± 11.4
Iorio et al. [48]	2021	Italy	29	No	Supine	62.7 ± 4.9	48.3	28.7 ± 3.4	49.2 ± 9.0
Jin et al. [49]	2023	China	50	No	Supine	51.4 ± 13.6	52.0	21.8 ± 2.2	49.8 ± 4.4
Kleinert et al. [50] ****	2012	Switzerland	80	Yes	Supine	65.0 ± 10.5	47.5	26.0 ± 7.9	53.0 ± 13.0
Mjaaland et al. [51]	2015	Norway	84	No	Supine	67.2 ± 8.6	31.3	27.2 ± 3.6	53.6 ± 13.7
Mjaaland et al. [52]	2019	Norway	84	No	Supine	67.0 ± 9.0	29.8	28.0 ± 4.0	53.6 ± 13.7
Moerenhout et al. [53]	2020	Canada	28	Yes	Supine	70.4 ± 9.1	64.3	27.6 ± 4.4	52.1 ± 19.7
Mortazavi et al. [54]	2022	Iran	77	No	NR	48.5 ± 14.7	55.8	26.1 ± 4.5	NR
Nambiar et al. [55]	2021	Australia	23	Yes	Supine	64.0 ± 11.0	47.8	27.0 ± 3.0	NR
Nistor et al. [56]	2017	Romania	35	No	Supine	67.0 ± 10.2	25.7	27.5 ± 3.8	NR
Parvizi et al. [57]	2016	USA	44	No	Supine	NR	40.1	NR	NR
Perry et al. [58]	2018	USA	25	Yes	NR	58.1 ± 4.8	40.0	NR	NR
Reichert et al. [59]	2018	Germany	73	No	Supine	62.5 ± 8.0	61.6	28.3 ± 4.0	54.0 ± 14.2
Restreppo et al. [60]	2010	USA	50	No	Supine	60.2 ± 10.2	34.0	25.2 ± 4.3	51.9 ± 7.9
Rykov et al. [61]**	2017	Netherlands	23	No	Supine	62.8 ± 6.1	34.8	29.0 ± 5.6	52.0 ± 6.7
Rykov et al. [62]**	2021	Netherlands	23	No	Supine	62.0 ± 9.0	34.8	27.8 ± 7.3	51.7 ± 6.7
Schwartz et al. [63]	2021	USA	48	Yes	Supine	62.0 ± 9.3	43.8	28.1 ± 4.8	NR
Suarez et al. [64]	2015	USA	61	Yes	Supine	64.7 ± 10.4	47.5	27.0 ± 4.5	NR
Taunton et al. [65]	2014	USA	27	Yes	Supine	62.1 ± 9.3	44.4	27.7 ± 4.8	55.0 ± 4.3
Taunton et al. [66]	2018	USA	52	Yes	Supine	65.0 ± 10.0	51.9	29.0 ± 5***	57.0 ± 13.0
Thaler et al. [67]	2018	Austria/Germany	16	No	Supine	66.0 ± 10.0	NR	27.0 ± 3.8	NR
Vandeputte et al. [68]	2021	Belgium	104	No	Supine	60.1 ± 15.5	35.6	27.1 ± 9.5	44.3 ± 21.0
Vles et al. [69]	2021	Belgium	60	No	Supine	64.0 ± 13.4	36.7	26.3 ± 4.4	NR
Wang et al. [70]	2020	China	50	No	Supine	55.9 ± 12.6	62.0	24.2 ± 2.9	NR
Xiao et al. [71]	2022	China	54	No	Supine	57.5 ± 13.6	55.6	24.0 ± 3.6	59.4 ± 20.3
Zhang et al. [72]	2021	China	58	No	Supine	68.5 ± 4.5	48.3	24.8 ± 2.8	24.2 ± 15.1
Zhao et al. [73]	2017	China	60	No	Supine	64.8 ± 12.3	40.0	24.3 ± 3.1	40.2 ± 9.2
Zhao et al. [74]	2020	China/USA	28	No	Supine	70.0 ± 5.1	28.6	NR	NR
Zhao et al. [75]****	2018	China	80	No	Supine	60.0 ± 10.8	56.3	22.4 ± 1.9	NR

RCT	Osteo-arthrosis, N	Dysplasia, N	ANFH, N	Fracture, N	Follow up, months	Outcome parameter	How was information on TT gathered?
Alvarez-Pinzon et al. [33]	21	0	4	0	3	1; 2; 3; 13	By phone or e-mail
Barrett et al. [34]*	NR	NR	NR	NR	12	1; 2; 3; 4; 5; 6; 8; 9; 10; 11; 13; 14; 15; 16; 21; 26	Clear description
Barrett et al. [35]*	NR	NR	NR	NR	60	21; 22	Clear description
Bon et al. [36]	NR	NR	NR	NR	3	1; 4; 12; 13; 14; 21; 22; 25; 27	By phone or e-mail
Brismar et al. [37]	NR	NR	NR	NR	60	1; 3; 21; 22; 23; 28;	Clear description
Brun et al. [38]	84	0	0	0	18	4	By phone or e-mail
Cheng et al. [39]	35	0	0	0	12	1; 2; 4; 21; 22; 24; 28	Clear description
Cooper et al. [40]	NR	NR	NR	NR	NR	21; 23; 28	By phone or e-mail
D'Arrigo et al. [41]	NR	NR	NR	NR	1,5	1; 3; 13; 21; 24; 26; 27;	By phone or e-mail
De Anta-Diaz et al. [42]	50	0	0	0	12	1; 2; 14; 16;	By phone or e-mail
Fahs et al. [43]	50	0	0	0	12	1; 5; 21; 27;	Clear description
Fraval et al. [44]	51	0	0	0	24	1; 3;	By phone or e-mail
Fraval et al. [45]	53	0	0	0	12	1; 3;	By phone or e-mail
Goyal et al. [46]	102	1	5	0	12	5; 8; 13; 21; 23; 28;	By phone or e-mail
Guild et al. [47]	NR	NR	NR	NR	24	1; 3; 13;	Clear description
Iorio et al. [48]	29	0	0	0	11	1; 6; 7; 21; 27;	By phone or e-mail
Jin et al. [49]	NR	NR	NR	NR	36	1; 2; 4; 5; 7; 8; 9; 12; 13; 14; 15; 16; 17; 21; 27;	Clear description
Kleinert et al. [50] ****	80	0	0	0	8	1; 3; 5; 6; 7; 14; 18; 21; 22; 28;	Clear description
Mjaaland et al. [51]	84	0	0	0	18	1; 2; 18; 19; 20;	Clear description
Mjaaland et al. [52]	84	0	0	0	24	2; 21; 24; 25; 27; 28;	Clear description
Moerenhout et al. [53]	NR	0	NR	0	55	1; 4; 8; 9; 10; 11; 12; 13; 14; 15; 16; 17; 21; 23; 28;	By phone or e-mail
Mortazavi et al. [54]	32	18	23	4	24	1; 20; 21; 23; 28;	
Nambiar et al. [55]	23	0	0	0	60	21; 23; 27; 28;	By phone or e-mail
Nistor et al. [56]	35	0	0	0	22	1; 2; 4; 5; 6; 7; 8; 9; 21; 24; 26; 27;	Clear description
Parvizi et al. [57]	44	0	0	0	24	1; 3;	Clear description
Perry et al. [58]	NR	NR	NR	NR	21	5; 8;	
Reichert et al. [59]	73	0	0	0	28	4; 8; 9; 10; 11; 13; 14; 15; 16; 21; 27; 28;	By phone or e-mail
Restreppo et al. [60]	50	0	0	0	24	1; 2; 3; 13; 15; 16; 17; 18;	Clear description
Rykov et al. [61]**	23	0	0	0	1,5	1; 3; 13; 18; 21; 23; 28;	By phone or e-mail
Rykov et al. [62]**	23	0	0	0	12	4; 16; 21; 22; 23; 27; 28;	By phone or e-mail
Schwartz et al. [63]	48	0	0	0	18	1; 3; 8; 10;	By phone or e-mail
Suarez et al. [64]	NR	NR	NR	NR	NR	1; 3; 18; 19; 20;	By phone or e-mail
Taunton et al. [65]	27	0	0	0	6	12; 13; 16; 21; 24; 28;	By phone or e-mail
Taunton et al. [66]	52	0	0	0	37	1; 4; 5; 14; 16; 21; 22; 28;	By phone or e-mail
Thaler et al. [67]	16	0	0	0	24	5; 17;	Clear description
Vandeputte et al. [68]	104	0	0	0	12	1; 4; 16; 21; 24;	By phone or e-mail
Vles et al. [69]	NR	0	NR	0	10	3; 18; 20;	By phone or e-mail
Wang et al. [70]	2	20	28	0	3	1; 4; 5; 6; 18; 19; 21; 27;	Clear description
Xiao et al. [71]	NR	NR	NR	NR	6	1; 3; 4; 8; 10; 12; 13; 15; 18; 20; 21; 22; 24;	By phone or e-mail
Zhang et al. [72]	18	0	27	13	9	1; 13; 14; 21; 27;	Clear description
Zhao et al. [73]	41	6	13	0	14	1; 2; 3; 4; 5; 6; 7; 14; 15; 21; 24;	Clear description
Zhao et al. [74]	0	0	0	28	12	5; 6; 13; 15; 18; 19;	Clear description
Zhao et al. [75]****	34	0	56	0	10	1; 3; 18; 19; 20;	By phone or e-mail

RCT: randomized controlled trials; TT: traction table; SD: standard deviation; BMI: Body Mass Index; HHS: Harris Hip Score; ANFH: avascular necrosis of the femoral head; DAA: direct anterior approach; NR: not reported; *These two RCTs included the same patient cohort with different follow-up period; **These two studies included the same patient cohort with different follow-up period; ***This SD value was an obvious typo of the original RCT as it is statistically impossible. To obtain reliable results, the original SD value of this RCT was replaced by a reliable SD value that was calculated from the extracted range; ****In these two RCTs, the DAA group data were calculated because the original data were split into two groups in relation to an outcome that was irrelevant to our research question. 1: operation time; 2: incision length; 3: intraoperative blood loss; 4: acetabular cup inclination; 5: VAS 1 day postoperatively; 6: VAS 2 days postoperatively; 7: VAS 3 days postoperatively; 8: VAS 2–6 weeks postoperatively: 9: VAS 2–3 months postoperatively; 10: VAS 6 months postoperatively; 11: VAS 12 months postoperatively; 12: HHS 1–3 weeks postoperatively; 13: HHS 4–6 weeks postoperatively; 14: HHS 2–3 months postoperatively; 15: HHS 6 months postoperatively; 16: HHS 12 months postoperatively; 17: HHS 24 months postoperatively; 18: Hb 1 day postoperatively; 19: Hb 2 days postoperatively; 20: Hb 3 days postoperatively; 21: overall complications; 22: dislocation; 23: infection; 24: periprosthetic fracture; 25: DVT/PE; 26: haematoma; 27: LFCN palsy; 28: reoperation

### RCT quality assessment

The revised JBI Critical Appraisal Tool for the assessment of risk of bias in RCTs was used to critically appraise the internal validity [19]. In addition to the overall assessment of study quality, the revised tool was designed to facilitate specific assessments of the bias domains to which the questions belong, if necessary. Thresholds for grading the severity of bias are not appropriate in the tool. It is recommended that results are presented using a checklist approach. The checklist uses ‘+’ for fulfilled, ‘−’ for unclear and ‘×’ for not fulfilled [19]. Publication bias for all RCTs was calculated, using the Egger’s test and it was presented in funnel plots [20].

### Missing data and data preparation

If relevant data was missing, the corresponding authors were contacted by email or phone. If the standard deviation (SD) was not reported, the missing SD value was replaced with the weighted average of the existing SDs (weighted average imputation) [21]. If information on the TT application was missing or was in doubt, the corresponding authors were strictly contacted so that the primary data do not provide us with any doubtful information about the TT application. When the RCTs provided different information on the intention-to-treat (ITT) analysis and the per-protocol (PP) analysis, the numbers from the ITT analysis were used. If the literature search identified 3-arm RCTs of DAA, one of the three patient groups was included in the common comparator group, and the other two patient groups were statistically combined and included in either the DAA with TT or DAA without TT treatment group. If an RCT investigated different DAA groups, the DAA group with the specific treatment (use of bone wax, special retraction system, etc.) was included in the common comparator group, and the RCT’s DAA control group without the specific treatment was included in either the network meta-analysis’ DAA with TT or the network meta-analysis’ DAA without TT treatment group. In this way, we have tried to ensure homogeneous treatment groups without interfering factors.

### Measures of treatment effect

#### Indirect comparison: network *meta*-analysis

An indirect comparison in network meta-analysis was performed to assess treatment effects between DAA on a TT and DAA on a standard table. The surgical approach or technique in THA to which DAA was compared in the primary RCT was used as a common comparator and reference node within the network. All analyses were conducted using fixed-effects and random-effects models estimated with frequentist approach and consistency assumption. In interpreting the meta-results, the random effects model was followed since it seems to be generalizable beyond the included RCTs, due to low to moderate heterogeneity and content validity of the included studies [22]. Standardized mean differences (SMDs) with 95% confidence intervals (CIs) were estimated for continuous variables and odds ratios (ORs) with 95% CIs were estimated for binary variables. Heterogeneity was assessed using a test on Cochrane’s Q statistic and Higgins’ I^2^ test. The meta-results were presented graphically in forest plots, where the results of each RCT were represented as boxes on a horizontal axis, with the size of the box indicating the statistical power of the study. The overall effect of all RCTs was illustrated with a rhombus. In the forest plot, the position of the rhombus along the abscissa favors either DAA on a TT or DAA on a standard operating table. If the rhombus does not cross the ordinate, these are significant results in favor of one of both groups. As we calculated SD values by imputation, we also performed a sensitivity analysis to check the robustness of the results after imputation. We added the weighted average and multiplied it by 1.5, which means that we increased the SD from imputation by 50%. All statistical analyses were performed by a professional statistician (RH) using netmeta and metaphor packages in the R software version 4.2.1 [23].

## Results

### Systematic review of literature

**Fig. 1:**
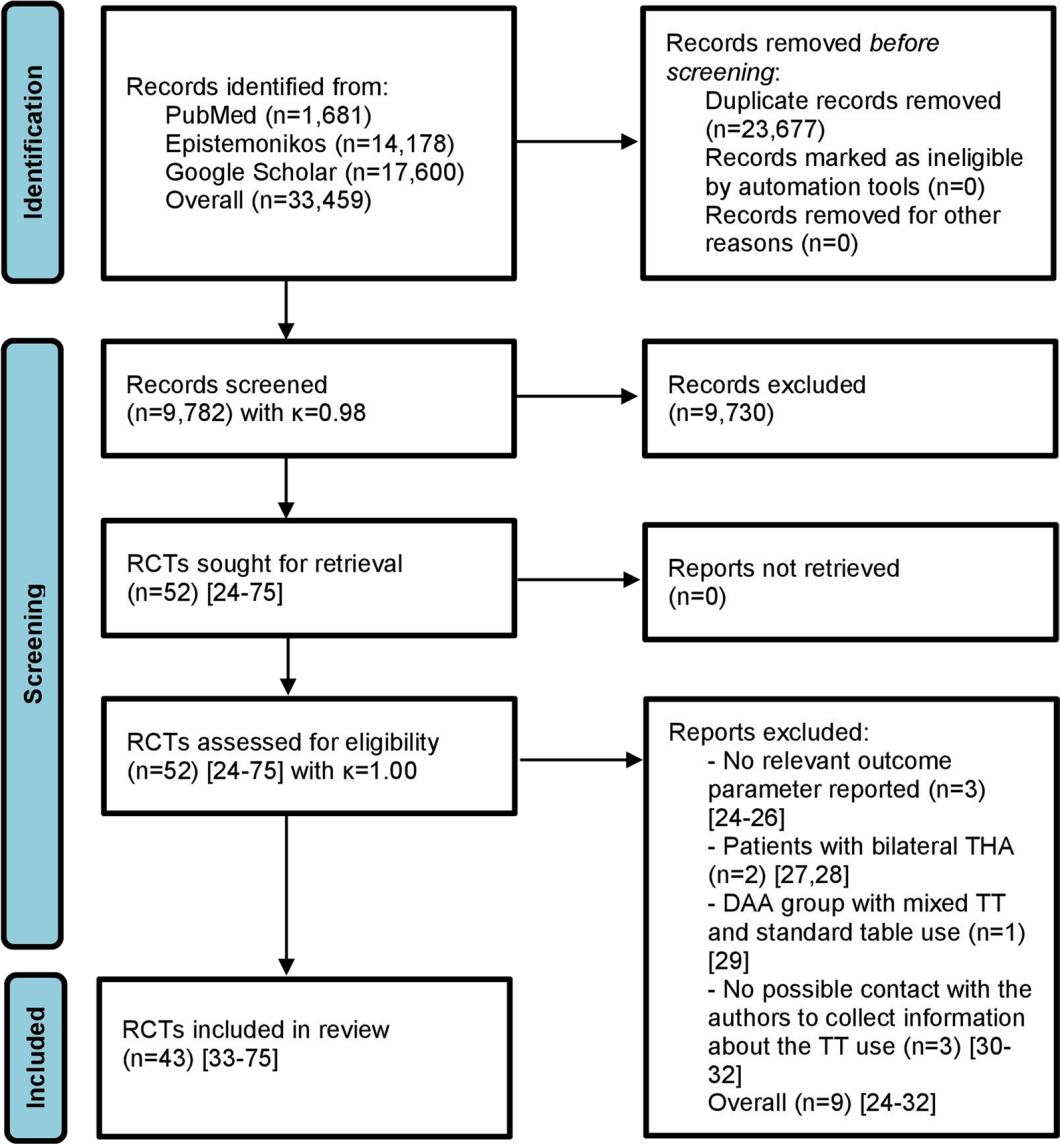
PRISMA flow diagram of the search results and selection according to our inclusion criteria. DAA: direct anterior approach; TT: traction table; RCT: randomized controlled trial; THA: total hip arthroplasty

After an initial literature search in PubMed, Epistemonikos and Google Scholar and a subsequent stepwise inclusion process, a total of 52 [24–75] were assessed for eligibility with full inter-reviewer agreement (κ = 1.0). After excluding 9 RCTs [24–32], 43 RCTs [33–75] with a total of 2,258 patients met the eligibility criteria for inclusion in the network meta-analysis (Fig. 1). Of these 43 RCTs [33–75], 17 RCTs [33–36, 39, 43–45, 47, 50, 53, 55, 58, 63–66] with a total of 804 patients reported THA using a TT and 26 RCTs [37, 38, 40–42, 46, 48, 49, 51, 52, 54, 56, 57, 59–62, 67–75] with a total of 1,454 patients reported THA using a standard operating table. Further information on the RCTs included [33–75] and patient characteristics are shown in Table 1. Some of the included RCTs had the same author group and the same hospital where the RCT was conducted [34, 35, 44, 45, 51, 52, 61, 62, 65, 66, 73, 75]. These RCTs were nevertheless included because the patient cohorts [44, 45, 61, 62, 65, 66, 73, 75] or at least the extracted outcome parameters were still different [34, 35, 51, 52]. This was the case for the following reasons: (i) the RCTs were conducted at different periods of time and had different patient cohorts [44, 45, 61, 62, 65, 66, 73, 75]; (ii) the RCTs had identical patient cohorts, but the outcome parameters were different, assessed and reported at different time points [34, 35, 51, 52].

Of the 43 RCTs included in this network meta-analysis, 24 were 2-arm RCTs comparing either DAA with TT or on a standard operating table with a conventional approach [34–39, 41, 42, 48, 49, 51–53, 55–57, 59–62, 65–67, 73]. Furthermore, of the 43 RCTs, two were 3-arm RCTs [50, 75]. Kleinert et al. [50] divided their patient cohort according to the postoperative redon drainage application. The first group of patients was treated postoperatively with a redon drain, the second group was treated postoperatively with a standard redon drain and the third group was treated with an investigated special drain. The first and second groups were statistically combined and included in the experimental group of the present network meta-analysis; the third group with the special drainage was included in the common comparator group of the present network meta-analysis. Zhao et al. [75] divided their patient cohort according to the tranexamic acid application. The first group of patients was treated postoperatively with oral tranexamic acid application, the second group was treated intraoperatively with intravenous tranexamic acid application and the third group was treated without tranxamic acid application. The first and second groups were statistically combined and included in the experimental group of the present network meta-analysis; the third group without tranxamic acid application was included in the common comparison group of the present network meta-analysis. Of the 43 RCTs included in this network meta-analysis, 17 were 2-arm RCTs [33, 40, 43–47, 54, 58, 63, 64, 68–72, 74], comparing two different DAA groups. The DAA group with the specific treatment (use of bone wax, special retraction system, etc.) was included in the common comparator group of the present network meta-analysis. During data extraction, an obvious typing error was found in the RCT by Taunton et al. [66]. In this RCT [66], the standard deviation value for BMI was calculated from the extracted range (calculated SD = 5). The calculated value of '5' replaced the original value of '22' as it could not possibly be statistically correct.

### RCT quality assessment

The results of the risk of bias quality assessment of the included RCTs using the revised JBI Critical Appraisal Tool varied from low to moderate (Table 2). The assessment of publication bias using the Egger’s test is shown in Table 3 (Table 3). The funnel plots for each outcome parameter are available in the supplement.

**Table 2:**
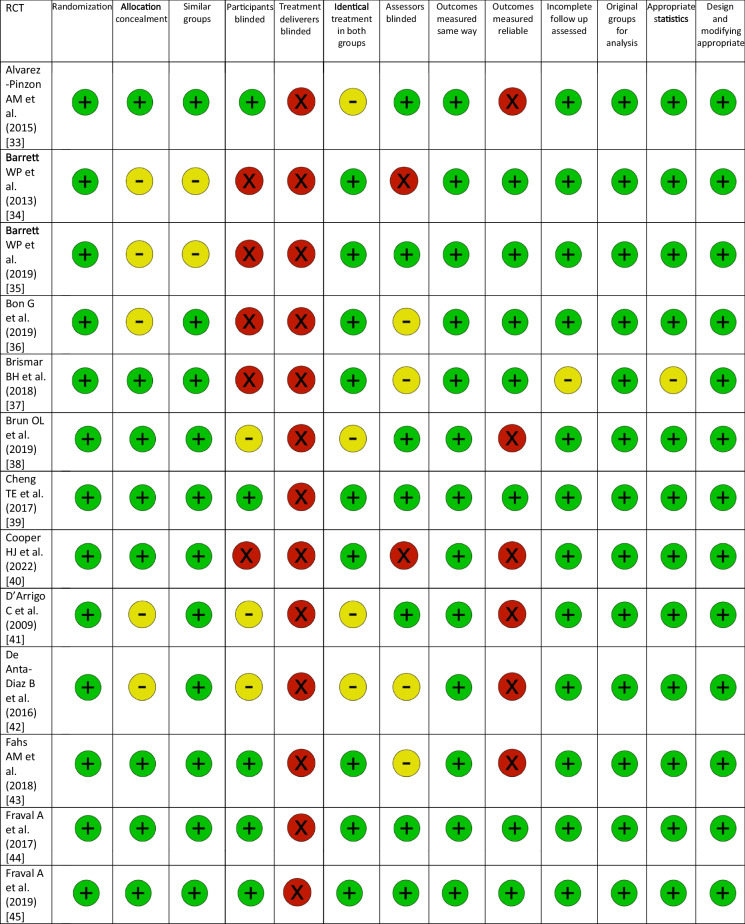

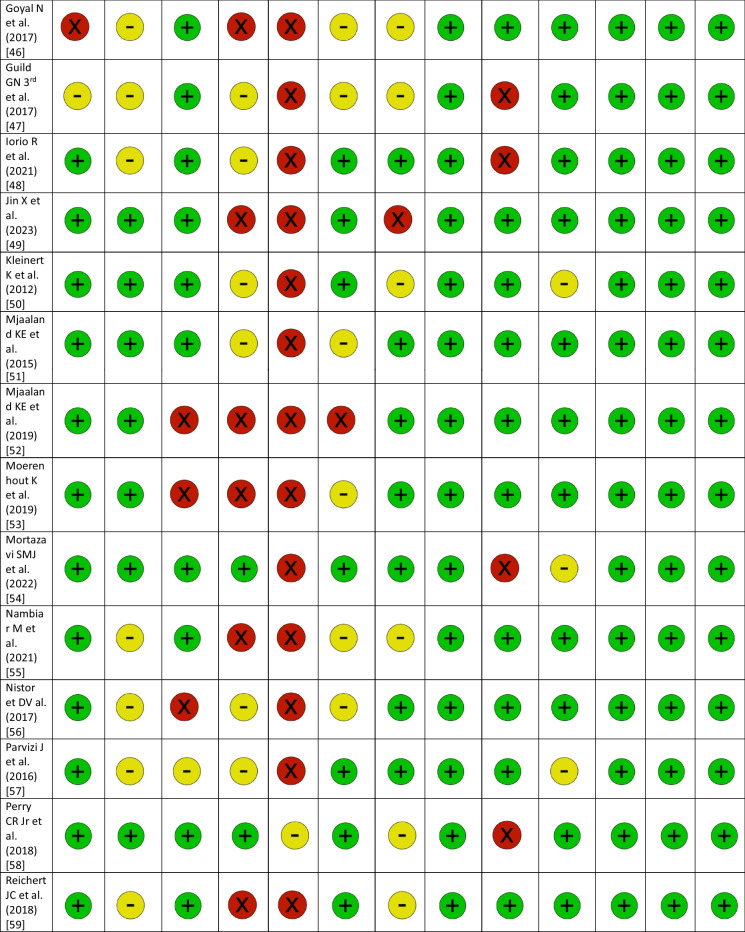

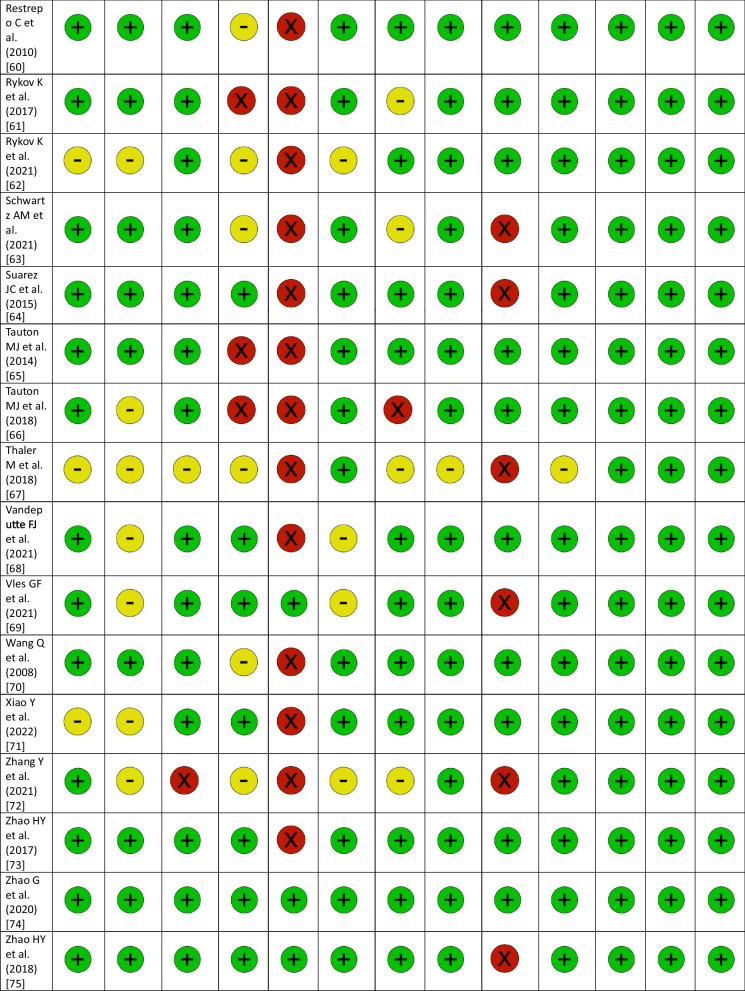
Assessment of risk of bias with the revised JBI Critical Appraisal Tool for RCTs

RCT: randomized controlled trial; (+): fulfilled; (−): unclear; (X): not fulfilled

**Table 3:** Results of the network meta-analysis for all outcome parameters included

	RCTs, N	Patients, N	Treatment effect: TT vs. no TT (SMD or OR)	95% CI	*P* value: TT versus no TT	Treatment effect: common comparator versus TT (SMD or OR)	*P* value : common comparator versus TT	Treatment effect: common comparator versus no TT (SMD or OR)
Operation time (min)	30	3238	0.51	− 7.27 to 8.36	0.89	− 8.11	< 0.01*	− 7.60
Incision length (cm)	10	1027	2.17	− 0.49 to 4.81	0.10	0.60	0.59	2.77
Intraoperative blood loss (mL)	17	1850	101.38	47.62–157.04	< 0.01*	− 126.66	< 0.01*	− 25.28
Acetabular cup inclination (°)	14	1447	1.05	− 1.25 to 3.34	0.37	− 0.54	0.58	0.51
VAS 1 day postopertively (points)	12	1154	0.147	− 0.63 to 0.87	0.72	0.34	0.28	0.48
VAS 2 days postopertively (points)	7	612	0.75	− 0.85 to 2.35	0.36	− 0.09	0.89	0.66
VAS 3 days postopertively (points)	5	470	0.59	− 0.24 to 1.42	0.16	0.00	1.00	0.59
VAS 2–6 weeks postopertively (points)	9	892	− 0.02	− 0.17 to 0.14	0.85	0.02	0.60	0.01
VAS 2–3 months postopertively (points)	5	435	0.12	− 0.97 to 1.21	0.83	0.10	0.82	0.22
VAS 6 months postopertively (points)	5	453	0.00	− 0.50 to 0.50	1.00	− 0.23	0.24	− 0.23
VAS 12 months postopertively (points)	3	265	− 0.69	− 1.63 to 0.24	0.15	− 0.01	0.98	− 0.70
HHS 1–3 weeks postopertively (points)	5	399	5.51	− 7.61 to 20.33	0.37	− 7.74	0.06	− 2.23
HHS 4–6 weeks postopertively (points)	15	1599	1.77	− 1.75 to 5.34	0.37	− 2.98	0.06	− 1.21
HHS 2–3 months postopertively (points)	10	1022	1.29	− 1.14 to 3.71	0.30	− 2.48	0.01*	− 1.19
HHS 6 months postopertively (points)	8	731	− 0.36	− 3.51 to 2.74	0.82	0.12	0.94	− 0.24
HHS 12 months postopertively (points)	10	984	− 0.20	− 1.00 to 2.22	0.85	− 1.15	0.19	− 1.36
HHS 24 months postopertively (points)	4	288	0.48	− 8.79 to 9.11	0.91	− 0.70	0.88	− 0.22
Hb 1 day postopertively (mmol/L)	10	1033	− 0.26	− 0.80 to 0.28	0.33	0.36	0.14	0.09
Hb 2 days postopertively (mmol/L)	5	557	− 0.28	− 1.16 to 0.61	0.54	0.36	0.37	0.09
Hb 3 days postopertively (mmol/L)	6	764	− 0.60	− 1.19 to 0.00	0.05*	0.81	< 0.01*	0.21
Overall complications	28	2941	0.46	0.16–1.34	0.16	0.96	0.93	0.45
Dislocation	10	927	0.87	0.20–3.76	0.85	1.09	0.89	0.94
Infection	11	1224	0.73	0.09–5.67	0.77	0.64	0.64	0.47
Periprosthetic fracture	12	1300	0.15	0.03–0.86	0.03*	1.48	0.53	0.22
DVT/PE	4	452	0.72	0.02–20.44	0.84	1.47	0.69	1.05
Haematoma	4	386	2.09	0.00–1119.02	0.82	0.32	0.68	0.67
LFCN palsy	12	1199	0.77	0.04–14.37	0.86	0.25	0.29	0.20
Reoperation	15	1513	0.98	0.30–3.19	0.97	0.96	0.94	0.94

	*P* value: common comparator versus no TT	I^2^ common comparator versus TT	τ^2^ common comparator versus TT	I^2^ common comparator versus no TT	τ^2^ common comparator versus no TT	Heterogeneity * P* value	Type of variable	Egger * P* value
Operation time (min.)	< 0.01*	0.89	70.90	0.95	95.30	< 0.01*	Continuous	0.02*
Incision length (cm)	< 0.01*	0.98	4.30	0.99	3.50	< 0.01*	Continuous	0.23
Intraoperative blood loss (mL)	0.24	0.96	2356.60	0.82	1787.20	< 0.01*	Continuous	0.06
Acetabular cup inclination (°)	0.44	0.79	6.60	0.72	2.00	< 0.01*	Continuous	0.79
VAS 1 day postopertively (points)	0.07	0.86	0.40	0.88	0.40	< 0.01*	Continuous	0.75
VAS 2 days postopertively (points)	0.13	0.79	0.30	0.98	0.90	< 0.01*	Continuous	0.66
VAS 3 days postopertively (points)	< 0.01*	N/A	N/A	0.83	0.10	< 0.01*	Continuous	0.81
VAS 2–6 weeks postopertively (points)	0.90	0.00	0.00	0.00	0.00	0.76	Continuous	0.78
VAS 2–3 months postopertively (points)	0.50	0.00	0.00	0.95	0.30	< 0.01*	Continuous	0.52
VAS 6 months postopertively (points)	0.16	0.09	0.00	0.70	0.00	0.13	Continuous	0.98
VAS 12 months postopertively (points)	0.06	0.68	0.10	N/A	N/A	0.08	Continuous	0.97
HHS 1–3 weeks postopertively (points)	0.62	0.76	45.50	0.91	33.70	< 0.01*	Continuous	0.41
HHS 4–6 weeks postopertively (points)	0.28	0.79	22.80	0.77	5.40	< 0.01*	Continuous	0.44
HHS 2–3 months postopertively (points)	0.09	0.54	5.00	0.16	0.20	0.10	Continuous	0.38
HHS 6 months postopertively (points)	0.66	0.00	0.00	0.32	0.70	0.29	Continuous	0.98
HHS 12 months postopertively (points)	0.02	0.00	0.00	0.55	0.20	0.12	Continuous	0.08
HHS 24 months postopertively (points)	0.54	N/A	N/A	0.00	0.00	0.64	Continuous	0.63
Hb 1 day postopertively (mmol/L)	0.46	0.00	0.00	0.83	0.10	< 0.01*	Continuous	0.39
Hb 2 days postopertively (mmol/L)	0.67	N/A	N/A	0.88	0.10	< 0.01*	Continuous	0.55
Hb 3 days postopertively (mmol/L)	0.09	N/A	N/A	0.73	0.10	< 0.01*	Continuous	0.78
Overall complications	0.01*	0.49	1.40	0.31	0.40	0.02*	Dichotomous	0.06
Dislocation	0.90	0.00	0.00	0.20	0.30	0.69	Dichotomous	0.39
Infection	0.08	0.00	0.00	0.00	0.00	0.94	Dichotomous	0.20
Periprosthetic fracture	0.02*	0.00	0.00	0.00	0.00	0.82	Dichotomous	0.02*
DVT/PE	0.98	0.00	0.00	N/A	N/A	0.53	Dichotomous	1.00
Haematoma	0.79	N/A	N/A	0.71	5.10	0.03*	Dichotomous	0.64
LFCN palsy	0.03*	0.81	7.80	0.64	2.40	< 0.01*	Dichotomous	0.02*
Reoperation	0.86	0.00	0.00	0.00	0.00	0.93	Dichotomous	0.80

RCT: randomized controlled trials; TT: traction table; SMD: standardized mean difference; OR: odds ratio; CI: confidence interval; VAS: visual analog scale; HHS: Harris Hip Score; Hb: hemoglobin; DVT: deep vein thrombosis; PE pulmonary embolism; LFCN: lateral femoral cutaneous nerve; *statistically significant; N/A: Not applicable (calculation was impossible due insufficient data)

### Indirect comparison in network *meta*-analysis

**Fig. 2:**
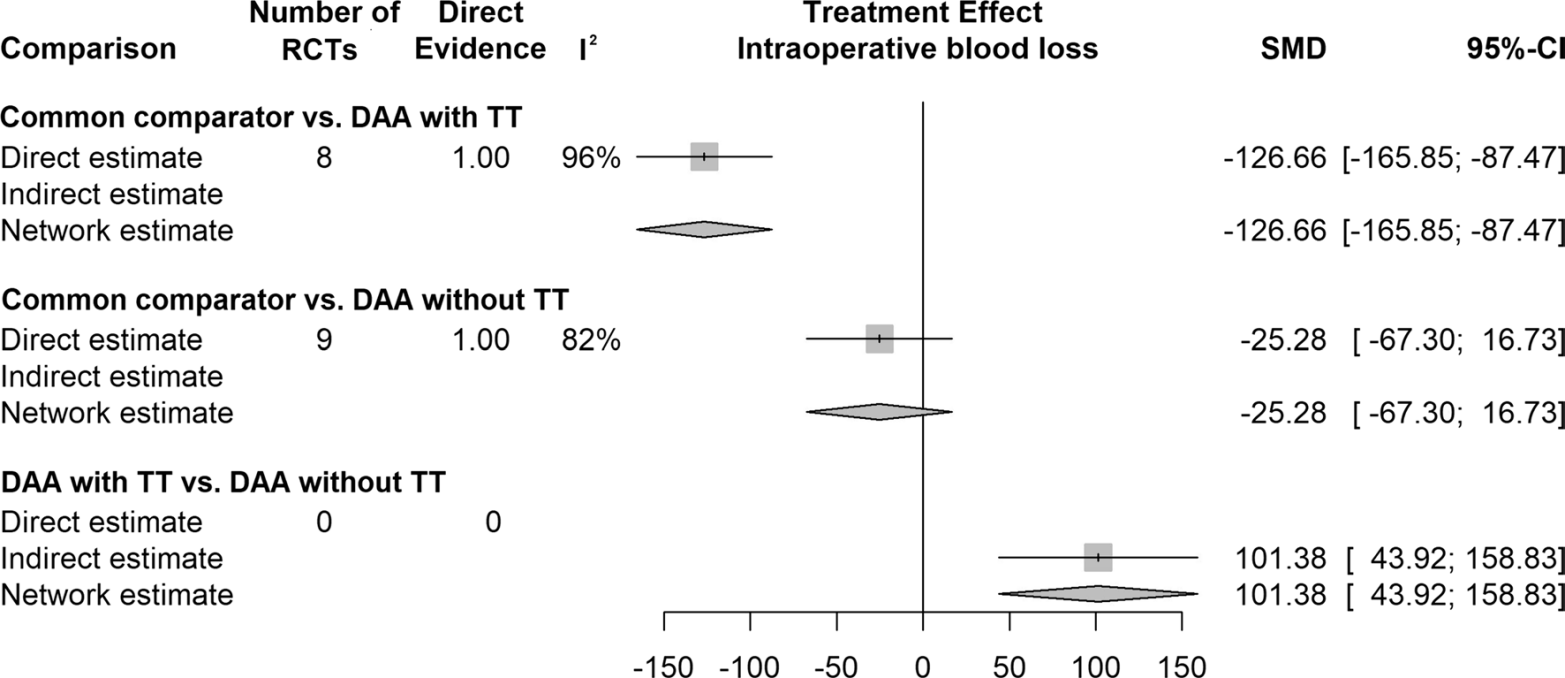
Forest plot of the intraoperative blood loss. The SMD of the summary measure has positive values, which favours DAA THA on a standard operating table (SMD = 102.33, 95% CI  47.62 to 157.04). RCT: randomized controlled trial; SMD: standardized mean difference; CI: confidence interval; DAA: direct anterior approach; TT: traction table

**Fig. 3:**
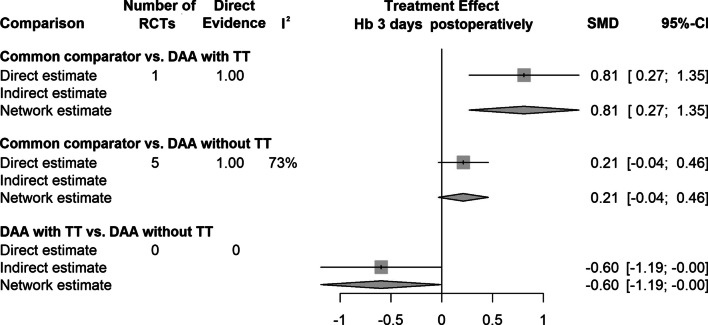
Forest plot of the Hb 3 days postoperatively. The SMD of the summary measure has negative values, which favours DAA THA on a standard operating table (SMD = − 0.60, 95% CI  − 1.19 to − 0.00). RCT: randomized controlled trial; SMD: standardized mean difference; CI: confidence interval; DAA: direct anterior approach; TT: traction table

**Fig. 4:**
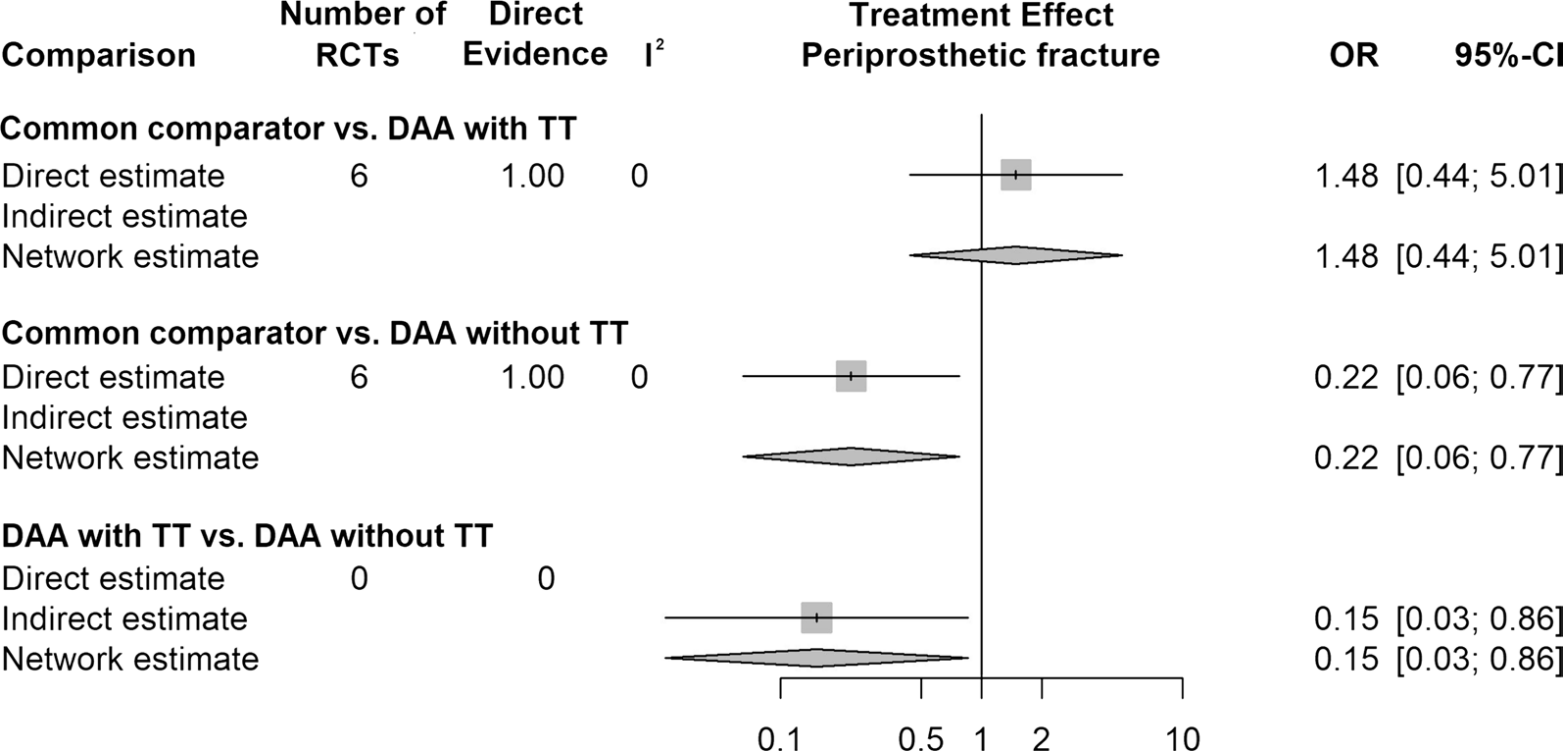
Forest plot of the periprosthetic fracture rate. The OR of the summary measure has values < 1, which favours DAA THA with TT (OR 0.15, 95% CI  0.03 to 0.86). RCT: randomized controlled trial; OR: odds ratio; CI: confidence interval; DAA: direct anterior approach; TT: traction table

The results of the network meta-analysis for all outcome parameters included are shown in Table 3. A summary of the extracted data showing the mean values of the continuous outcome parameters and the event percentages of the dichotomous outcome parameters is shown in Table 4 and 5. The 3 outcome parameters that showed statistically significant differences are presented in forest plots (Fig. 2–4). The forest plots for each outcome parameters are available in the supplement.

**Table 4:** Summary of the extracted data showing the mean values of the continuous outcome parameters

RCT	DAA-group (TT, no TT)	DAA THA patients	Operation time (min.)	Incision length (cm)	Intraoperative blood loss (mL)	Cup inclination (°)	VAS 1 day postoperatively (points)	VAS 2 days postoperatively (points)
		N	Mean, SD	Mean, SD	Mean, SD	Mean, SD	Mean, SD	Mean, SD
Alvarez-Pinzon et al. [33]	TT	25	114.0 ± 16.0	12.0 ± 0.9	444.0 ± 258.0	NR	NR	NR
Barrett et al. [34]	TT	43	84.3 ± 12.4	13.7 ± 0.9	391.0 ± 206.0	47.1 ± 6.1	4.0 ± 1.0	3.8 ± 1.1
Barrett et al. [35]	TT	43	NR	NR	NR	NR	NR	NR
Bon et al. [36]	TT	50	70.1 ± 11.0	NR	NR	37.7 ± 4.2	NR	NR
Brismar et al. [37]	no TT	50	101.0 ± 6.3	NR	325.0 ± 75.0	NR	NR	NR
Brun et al. [38]	no TT	84	NR	NR	NR	49.5 ± 7.4	NR	NR
Cheng et al. [39]	TT	35	125.0 ± 6.8	10.7 ± 0.8	NR	46.2 ± 5.6	NR	NR
Cooper et al. [40]	no TT	60	NR	NR	NR	NR	NR	NR
D'Arrigo et al. [41]	no TT	20	121.0 ± 23.6	NR	1344.0 ± 710.0	NR	NR	NR
De Anta-Diaz et al. [42]	no TT	50	78.2 ± 16.2	10.4 ± 0.9	NR	NR	NR	NR
Fahs et al. [43]	TT	50	88.9 ± 10.6	NR	NR	NR	2.9 ± 2.2	NR
Fraval et al. [44]	TT	51	63.7 ± 13.0	NR	687.0 ± 13.0	NR	NR	NR
Fraval et al. [45]	TT	53	63.8 ± 13.1	NR	690.0 ± 30.0	NR	NR	NR
Goyal et al. [46]	no TT	108	NR	NR	NR	NR	2.8 ± 2.1	NR
Guild et al. [47]	TT	110	124.8 ± 28.2	NR	383.4 ± 320.1	NR	NR	NR
Iorio et al. [48]	no TT	29	92.0 ± 11.0	NR	NR	NR	NR	2.9 ± 0.4
Jin et al. [49]	no TT	50	169.7 ± 17.3	9.7 ± 1.6	NR	38.7 ± 2.6	3.2 ± 1.1	NR
Kleinert et al. [50]	TT	80	115.0 ± 25.8	NR	408.0 ± 229.5	NR	1.8 ± 1.8	1.2 ± 1.9
Mjaaland et al. [51]	no TT	84	77.0 ± 21.0	9.5 ± 1.3	NR	NR	NR	NR
Mjaaland et al. [52]	no TT	84	NR	8.0 ± 1.2	NR	NR	NR	NR
Moerenhout et al. [53]	TT	28	59.9 ± 12.7	NR	NR	43.3 ± 8.4	NR	NR
Mortazavi et al. [54]	no TT	77	76.9 ± 12.9	NR	NR	NR	NR	NR
Nambiar et al. [55]	TT	23	NR	NR	NR	NR	NR	NR
Nistor et al. [56]	no TT	35	70.0 ± 1.3	12.2 ± 1.9	NR	37.0 ± 5.1	1.0 ± 1.3	1.0 ± 0.4
Parvizi et al. [57]	no TT	44	84.5 ± 14.5	NR	257.4 ± 201.7	NR	NR	NR
Perry et al. [58]	TT	25	NR	NR	NR	NR	3.9 ± 0.9	NR
Reichert et al. [59]	no TT	73	NR	NR	NR	38.6 ± 5.1	NR	NR
Restreppo et al. [60]	no TT	50	56.4 ± 14.5	10.1 ± 1.2	172.5 ± 201.7	NR	NR	NR
Rykov et al. [61]	no TT	23	71.0 ± 7.0	NR	325.7 ± 99.7	NR	NR	NR
Rykov et al. [62]	no TT	23	NR	NR	NR	47.0 ± 6.0	NR	NR
Schwartz et al. [63]	TT	48	74.6 ± 11.2	NR	359.7 ± 154.3	NR	NR	NR
Suarez et al. [64]	TT	61	92.3 ± 16.3	NR	469.6 ± 216.4	NR	NR	NR
Taunton et al. [65]	TT	27	NR	NR	NR	NR	NR	NR
Taunton et al. [66]	TT	52	70.0 ± 16.0	NR	NR	37.0 ± 5.0	2.0 ± 1.0	NR
Thaler et al. [67]	no TT	16	NR	NR	NR	NR	1.0 ± 0.3	NR
Vandeputte et al. [68]	no TT	104	48.7 ± 17.3	NR	NR	33.2 ± 5.3	NR	NR
Vles et al. [69]	no TT	60	NR	NR	370.0 ± 320.0	NR	NR	NR
Wang et al. [70]	no TT	50	66.8 ± 6.8	NR	NR	41.4 ± 4.3	3.3 ± 0.6	2.9 ± 0.6
Xiao et al. [71]	no TT	54	106.1 ± 47.6	NR	444.4 ± 486.8	39.7 ± 6.8	NR	NR
Zhang et al. [72]	no TT	58	82.2 ± 5.2	NR	NR	NR	NR	NR
Zhao et al. [73]	no TT	60	83.2 ± 4.6	9.1 ± 0.5	165.9 ± 42.6	40.3 ± 2.8	3.1 ± 0.8	2.1 ± 0.3
Zhao et al. [74]	no TT	28	NR	NR	NR	NR	8.0 ± 1.3	7.0 ± 0.4
Zhao et al. [75]	no TT	80	63.5 ± 11.5	NR	133.7 ± 21.1	NR	NR	NR
	Overall RCTs, N	Overall patients, N	Mean	Mean	Mean	Mean	Mean	Mean
TT	17	804	88.2	12.1	479.1	42.3	2.9	2.5
no TT	26	1454	85.2	9.9	393.2	40.6	3.2	3.2

RCT	VAS 3 days postoperatively (points)	VAS 2–6 weeks postoperatively (points)	VAS 2–3 months postoperatively (points)	VAS 6 months postoperatively (points)	VAS 12 months postoperatively (points)
	Mean, SD	Mean, SD	Mean, SD	Mean, SD	Mean, SD
Alvarez-Pinzon et al. [33]	NR	NR	NR	NR	NR
Barrett et al. [34]	NR	1.9 ± 1.2	1.3 ± 0.5	1.6 ± 1.5	1.6 ± 1.4
Barrett et al. [35]	NR	NR	NR	NR	NR
Bon et al. [36]	NR	NR	NR	NR	NR
Brismar et al. [37]	NR	NR	NR	NR	NR
Brun et al. [38]	NR	NR	NR	NR	NR
Cheng et al. [39]	NR	NR	NR	NR	NR
Cooper et al. [40]	NR	NR	NR	NR	NR
D'Arrigo et al. [41]	NR	NR	NR	NR	NR
De Anta-Diaz et al. [42]	NR	NR	NR	NR	NR
Fahs et al. [43]	NR	NR	NR	NR	NR
Fraval et al. [44]	NR	NR	NR	NR	NR
Fraval et al. [45]	NR	NR	NR	NR	NR
Goyal et al. [46]	NR	1.7 ± 1.9	NR	NR	NR
Guild et al. [47]	NR	NR	NR	NR	NR
Iorio et al. [48]	2.7 ± 0.6	NR	NR	NR	NR
Jin et al. [49]	2.2 ± 0.9	1.6 ± 0.5	1.4 ± 0.5	NR	NR
Kleinert et al. [50]	0.7 ± 1.2	NR	NR	NR	NR
Mjaaland et al. [51]	NR	NR	NR	NR	NR
Mjaaland E et al. [52]	NR	NR	NR	NR	NR
Moerenhout et al. [53]	NR	1.7 ± 2.0	1.0 ± 1.7	0.4 ± 0.8	0.3 ± 0.5
Mortazavi et al. [54]	NR	NR	NR	NR	NR
Nambiar et al. [55]	NR	NR	NR	NR	NR
Nistor et al. [56]	2.0 ± 0.6	1.2 ± 1.0	0.0 ± 0.7	NR	NR
Parvizi et al. [57]	NR	NR	NR	NR	NR
Perry et al. [58]	NR	0.3 ± 0.1	NR	NR	NR
Reichert et al. [59]	NR	6.9 ± 0.7	7.3 ± 0.8	7.3 ± 0.7	7.7 ± 0.6
Restreppo et al. [60]	NR	NR	NR	NR	NR
Rykov et al. [61]	NR	NR	NR	NR	NR
Rykov et al. [62]	NR	NR	NR	NR	NR
Schwartz et al. [63]	NR	0.2 ± 0.2	NR	1.0 ± 1.9	NR
Suarez et al. [64]	NR	NR	NR	NR	NR
Taunton et al. [65]	NR	NR	NR	NR	NR
Taunton et al. [66]	NR	NR	NR	NR	NR
Thaler et al. [67]	NR	NR	NR	NR	NR
Vandeputte et al. [68]	NR	NR	NR	NR	NR
Vles et al. [69]	NR	NR	NR	NR	NR
Wang et al. [70]	NR	NR	NR	NR	NR
Xiao et al. [71]	NR	2.0 ± 1.0	NR	0.4 ± 0.6	NR
Zhang et al. [72]	NR	NR	NR	NR	NR
Zhao et al. [73]	1.8 ± 0.4	NR	NR	NR	NR
Zhao et al. [74]	NR	NR	NR	NR	NR
Zhao et al. [75]	NR	NR	NR	NR	NR
	Mean	Mean	Mean	Mean	Mean
TT	0.7	1.0	1.2	1.0	1.0
No TT	2.2	2.7	2.9	3.9	7.7

RCT: randomized controlled trials; DAA: direct anterior approach; TT: traction table; THA: total hip arthroplasty; VAS: visual analog scale; SD: standard deviation; NR: not reported;

**Table 5:** Summary of the extracted data showing the mean values of the continuous outcome parameters and the event percentages of the dichotomous outcome parameters (Continuation of Table 4)

RCT	DAA-group (TT, no TT)	DAA THA patients	HHS 1–3 weeks postoperatively (points)	HHS 4–6 weeks postoperatively (points)	HHS 2–3 months postoperatively (points)	HHS 6 months postoperatively (points)	HHS 12 months postoperatively (points)	HHS 24 months postoperatively (points)	Hb 1 day postoperatively (points)	Hb 2 days postoperatively (points)	Hb 3 days postoperatively (points)
			Mean, SD	Mean, SD	Mean, SD	Mean, SD	Mean, SD	Mean, SD	Mean, SD	Mean, SD	Mean, SD
Alvarez-Pinzon et al. [33]	TT	25	NR	86.3 ± 15.0	NR	NR	NR	NR	NR	NR	NR
Barrett et al. [34]	TT	43	NR	89.5 ± 8.1	91.2 ± 9.7	95.8 ± 7.8	97.5 ± 5.7	NR	NR	NR	NR
Barrett et al. [35]	TT	43	NR	NR	NR	NR	NR	NR	NR	NR	NR
Bon et al. [36]	TT	50	69.5 ± 15.4	83.5 ± 13.4	90.0 ± 12.7	NR	NR	NR	NR	NR	NR
Brismar et al. [37]	no TT	50	NR	NR	NR	NR	NR	NR	NR	NR	NR
Brun et al. [38]	no TT	84	NR	NR	NR	NR	NR	NR	NR	NR	NR
Cheng et al. [39]	TT	35	NR	NR	NR	NR	NR	NR	NR	NR	NR
Cooper et al. [40]	no TT	60	NR	NR	NR	NR	NR	NR	NR	NR	NR
D'Arrigo et al. [41]	no TT	20	NR	88.3 ± 8.0	NR	NR	NR	NR	NR	NR	NR
De Anta-Diaz et al. [42]	no TT	50	NR	NR	94.6 ± 10.2	NR	96.2 ± 10.1	NR	NR	NR	NR
Fahs et al. [43]	TT	50	NR	NR	NR	NR	NR	NR	NR	NR	NR
Fraval et al. [44]	TT	51	NR	NR	NR	NR	NR	NR	NR	NR	NR
Fraval et al. [45]	TT	53	NR	NR	NR	NR	NR	NR	NR	NR	NR
Goyal et al. [46]	no TT	108	NR	75.0 ± 14.0	NR	NR	NR	NR	NR	NR	NR
Guild et al. [47]	TT	110	NR	87.4 ± 11.2	NR	NR	NR	NR	NR	NR	NR
Iorio et al. [48]	no TT	29	NR	NR	NR	NR	NR	NR	NR	NR	NR
Jin et al. [49]	no TT	50	82.1 ± 3.4	90.0 ± 2.9	92.5 ± 2.3	94.8 ± 2.5	95.0 ± 2.1	95.3 ± 1.8	NR	NR	NR
Kleinert et al. [50]	TT	80	NR	NR	88.0 ± 11.4	NR	NR	NR	6.2 ± 0.9	NR	NR
Mjaaland et al. [51]	no TT	84	NR	NR	NR	NR	NR	NR	6.9 ± 0.7	6.8 ± 0.8	6.8 ± 0.7
Mjaaland et al. [52]	no TT	84	NR	NR	NR	NR	NR	NR	NR	NR	NR
Moerenhout et al. [53]	TT	28	66.9 ± 17.1	76.7 ± 16.4	88.4 ± 11.8	90.1 ± 11.3	94.4 ± 8.0	89.4 ± 11.9	NR	NR	NR
Mortazavi et al. [54]	no TT	77	NR	NR	NR	NR	NR	NR	NR	NR	7.0 ± 0.9
Nambiar et al. [55]	TT	23	NR	NR	NR	NR	NR	NR	NR	NR	NR
Nistor et al. [56]	no TT	35	NR	NR	NR	NR	NR	NR	NR	NR	NR
Parvizi et al. [57]	no TT	44	NR	NR	NR	NR	NR	NR	NR	NR	NR
Perry al. [58]	TT	25	NR	NR	NR	NR	NR	NR	NR	NR	NR
Reichert et al. [59]	no TT	73	NR	81.6 ± 12.2	89.8 ± 9.3	90.3 ± 9.8	92.4 ± 8.6	NR	NR	NR	NR
Restreppo et al. [60]	no TT	50	NR	93.6 ± 5.7	NR	94.5 ± 5.7	94.7 ± 5.7	97.3 ± 1.7	6.7 ± 0,6	NR	NR
Rykov et al. [61]	no TT	23	NR	93.0 ± 10.9	NR	NR	NR	NR	7.8 ± 0.9	NR	NR
Rykov et al. [62]	no TT	23	NR	NR	NR	NR	98.1 ± 2.8	NR	NR	NR	NR
Schwartz et al. [63]	TT	48	NR	NR	NR	NR	NR	NR	NR	NR	NR
Suarez et al. [64]	TT	61	NR	NR	NR	NR	NR	NR	6.1 ± 0.7	5.9 ± 0.8	5.4 ± 0.6
Taunton et al. [65]	TT	27	97.0 ± 2.0	97.0 ± 2.0	NR	NR	98.0 ± 5.5	NR	NR	NR	NR
Taunton et al. [66]	TT	52	NR	NR	95.0 ± 6.0	NR	97.0 ± 4.0	NR	NR	NR	NR
Thaler et al. [67]	no TT	16	NR	NR	NR	NR	NR	100.0 ± 1.3	NR	NR	NR
Vandeputte et al. [68]	no TT	104	NR	NR	NR	NR	82.5 ± 19.5	NR	NR	NR	NR
Vles et al. [69]	no TT	60	NR	NR	NR	NR	NR	NR	7.3 ± 0.9	NR	6.8 ± 0.7
Wang et al. [70]	no TT	50	NR	NR	NR	NR	NR	NR	7.0 ± 0.6	6.6 ± 0.6	NR
Xiao et al. [71]	no TT	54	81.3 ± 8.2	94.1 ± 5.3	NR	96.6 ± 7.3	NR	NR	6.5 ± 1.1	NR	6.0 ± 1.0
Zhang et al. [72]	no TT	58	NR	80.0 ± 3.7	90.0 ± 3.8	NR	NR	NR	NR	NR	NR
Zhao et al. [73]	no TT	60	NR	NR	85.9 ± 17.4	92.2 ± 13.3	NR	NR	NR	NR	NR
Zhao et al. [74]	no TT	28	NR	70.0 ± 8.0	NR	82.0 ± 8.1	NR	NR	6.2 ± 0.8	5.6 ± 0.7	NR
Zhao et al. [75]	no TT	80	NR	NR	NR	NR	NR	NR	7.6 ± 0.8	6.4 ± 0.7	5.9 ± 0.8
	Overall RCTs, N	Overall patients, N	Mean	Mean	Mean	Mean	Mean	Mean	Mean	Mean	Mean
TT	17	804	77.8	86.7	90.5	93.0	96.7	89.4	6.2	5.9	5.4
no TT	26	1454	81.7	85.1	90.6	91.7	93.2	97.6	7.0	6.3	6.5

RCT	Overall complications	Dislocation	Infection	Periprosthetic fracture	DVT /PE	Haematoma	LFCN palsy	Reoperation
	N (%)	N (%)	N (%)	N (%)	N (%)	N (%)	N (%)	N (%)
Alvarez-Pinzon et al. [33]	0 (0.0)	0 (0.0)	0 (0.0)	0 (0.0)	0 (0.0)	0 (0.0)	0 (0.0)	0.0 (0.0)
Barrett et al. [34]	1 (2.3)	0 (0.0)	0 (0.0)	0 (0.0)	0 (0.0)	1 (2.3)	0 (0.0)	0 (0.0)
Barrett et al. [35]	1 (2.3)	1 (2.3)	0 (0.0)	0 (0.0)	0 (0.0)	0 (0.0)	0 (0.0)	0 (0.0)
Bon et al. [36]	10 (20.0)	1 (2.0)	0 (0.0)	0 (0.0)	1 (2.0)	0 (0.0)	8 (16.0)	0 (0.0)
Brismar et al. [37]	7 (14.0)	5 (10.0)	1 (10.0)	0 (0.0)	0 (0.0)	0 (0.0)	0 (0.0)	2 (4.0)
Brun et al. [38]	NR	NR	NR	NR	NR	NR	NR	NR
Cheng et al. [39]	3 (8.6)	1 (2.9)	0 (0.0)	2 (5.7)	0 (0.0)	0 (0.0)	0 (0.0)	1 (2.9)
Cooper et al. [40]	11 (18.3)	0 (0.0)	9 (15.0)	0 (0.0)	0 (0.0)	0 (0.0)	0 (0.0)	7 (11.7)
D'Arrigo et al. [41]	2 (10.0)	0 (0.0)	0 (0.0)	2 (10.0)	0 (0.0)	2 (10.0)	2 (10.0)	0 (0.0)
De Anta-Diaz et al. [42]	NR	NR	NR	NR	NR	NR	NR	NR
Fahs et al. [43]	1 (2.0)	0 (0.0)	0 (0.0)	0 (0.0)	0 (0.0)	0 (0.0)	1 (2.0)	0 (0.0)
Fraval et al. [44]	0 (0.0)	0 (0.0)	0 (0.0)	0 (0.0)	0 (0.0)	0 (0.0)	0 (0.0)	0 (0.0)
Fraval et al. [45]	0 (0.0)	0 (0.0)	0 (0.0)	0 (0.0)	0 (0.0)	0 (0.0)	0 (0.0)	0 (0.0)
Goyal et al. [46]	1 (0.9)	0 (0.0)	1 (0.9)	0 (0.0)	0 (0.0)	0 (0.0)	0 (0.0)	1 (0.9)
Guild et al. [47]	NR	NR	NR	NR	NR	NR	NR	NR
Iorio et al. [48]	2 (6.9)	0 (0.0)	0 (0.0)	0 (0.0)	0 (0.0)	0 (0.0)	2 (6.9)	0 (0.0)
Jin et al. [49]	12 (24.0)	0 (0.0)	0 (0.0)	0 (0.0)	0 (0.0)	0 (0.0)	12 (24.0)	0 (0.0)
Kleinert et al. [50]	3 (3.8)	2 (2.5)	0 (0.0)	0 (0.0)	0 (0.0)	0 (0.0)	0 (0.0)	1 (1.3)
Mjaaland et al. [51]	NR	NR	NR	NR	NR	NR	NR	NR
Mjaaland et al. [52]	3 (3.6)	0 (0.0)	0 (0.0)	1 (1.2)	1 (1.2)	0 (0.0)	1 (1.2)	1 (1.2)
Moerenhout et al. [53]	1 (3.6)	0 (0.0)	1 (3.6)	0 (0.0)	0 (0.0)	0 (0.0)	0 (0.0)	1 (3.6)
Mortazavi et al. [54]	2 (2.6)	0 (0.0)	2 (2.6)	0 (0.0)	0 (0.0)	0 (0.0)	0 (0.0)	1 (1.3)
Nambiar et al. [55]	9 (39.1)	0 (0.0)	1 (4.3)	0 (0.0)	0 (0.0)	0 (0.0)	8 (34.8)	1 (4.3)
Nistor et al. [56]	4 (11.4)	0 (0.0)	0 (0.0)	1 (2.9)	0 (0.0)	1 (2.9)	2 (2.9)	0 (0.0)
Parvizi et al. [57]	NR	NR	NR	NR	NR	NR	NR	NR
Perry et al. [58]	0 (0.0)	0 (0.0)	0 (0.0)	0 (0.0)	0 (0.0)	0 (0.0)	0 (0.0)	0 (0.0)
Reichert et al. [59]	4 (5.5)	0 (0.0)	0 (0.0)	0 (0.0)	0 (0.0)	0 (0.0)	3 (4.1)	1 (1.4)
Restreppo et al. [60]	0 (0.0)	0 (0.0)	0 (0.0)	0 (0.0)	0 (0.0)	0 (0.0)	0 (0.0)	0 (0.0)
Rykov et al. [61]	2 (8.7)	0 (0.0)	2 (8.7)	0 (0.0)	0 (0.0)	0 (0.0)	0 (0.0)	2 (8.7)
Rykov et al. [62]	5 (21.7)	10 (43.5)	2 (8.7)	0 (0.0)	0 (0.0)	0 (0.0)	1 (4.3)	12 (52.2)
Schwartz et al. [63]	0 (0.0)	0 (0.0)	0 (0.0)	0 (0.0)	0 (0.0)	0 (0.0)	0 (0.0)	0 (0.0)
Suarez et al. [64]	NR	NR	NR	NR	NR	NR	NR	NR
Taunton et al. [65]	2 (7.4)	0 (0.0)	0 (0.0)	2 (7.4)	0 (0.0)	0 (0.0)	0 (0.0)	2 (7.4)
Taunton et al. [66]	1 (1.9)	1 (1.9)	0 (0.0)	0 (0.0)	0 (0.0)	0 (0.0)	0 (0.0)	2 (3.8)
Thaler et al. [67]	NR	NR	NR	NR	NR	NR	NR	NR
Vandeputte et al. [68]	1 (9.6)	0 (0.0)	0 (0.0)	1 (9.6)	0 (0.0)	0 (0.0)	0 (0.0)	0 (0.0)
Vles et al. [69]	NR	NR	NR	NR	NR	NR	NR	NR
Wang et al. [70]	7 (14.0)	0 (0.0)	0 (0.0)	0 (0.0)	0 (0.0)	0 (0.0)	7 (14.0)	0 (0.0)
Xiao et al. [71]	3 (5.6)	2 (3.7)	0 (0.0)	1 (1.9)	0 (0.0)	0 (0.0)	0 (0.0)	0 (0.0)
Zhang et al. [72]	15 (25.9)	0 (0.0)	0 (0.0)	0 (0.0)	0 (0.0)	0 (0.0)	15 (25.9)	0 (0.0)
Zhao et al. [73]	1 (1.7)	0 (0.0)	0 (0.0)	1 (1.7)	0 (0.0)	0 (0.0)	0 (0.0)	0 (0.0)
Zhao et al. [74]	NR	NR	NR	NR	NR	NR	NR	NR
Zhao et al. [75]	0 (0.0)	0 (0.0)	0 (0.0)	0 (0.0)	0 (0.0)	0 (0.0)	0 (0.0)	0 (0.0)
	Event percentage	Event percentage	Event percentage	Event percentage	Event percentage	Event percentage	Event percentage	Event percentage
TT	5.1	0.9	0.3	0.6	0.2	0.2	2.7	1.3
No TT	7.5	1.6	1.6	0.6	0.1	0.3	4.1	2.4

RCT: randomized controlled trials; DAA: direct anterior approach; TT: traction table; THA: total hip arthroplasty; HHS: Harris Hip Score; Hb: hemoglobin; DVT: deep vein thrombosis; PE: pulmonary embolism; LFCN: lateral femoral cutaneous nerve; SD: standard deviation; NR: not reported

### Intraoperative blood loss

In an indirect comparison between DAA with TT and DAA without TT, data on 1850 patients were pooled from 17 RCTs (*p* < 0.01, Fig. 2, Tables 3, 4). DAA with TT had a 101.38 mL higher intraoperative blood loss compared with DAA without TT (SMD = 101.38, 95% CI 43.92 to 158.83).

### Hb 3 days postoperatively

In an indirect comparison between DAA with TT and DAA without TT, data on 764 patients were pooled from 6 RCTs (*p* = 0.05, Fig. 3, Tables 3, 5). DAA with TT had a 0.60 mmol/L lower Hb 3 days postoperatively compared with DAA without TT (SMD = − 0.60, 95% CI − 1.19 to − 0.00).

### Periprosthetic fracture

In an indirect comparison between DAA with TT and DAA without TT, data on 1300 patients were pooled from 12 RCTs (*p* = 0.03, Fig. 4, Tables 3, 5). DAA with TT had a 0.15 lower periprosthetic fracture rate compared with DAA without TT (OR 0.15, 95% CI 0.03 to 0.86).

### Sensitivity analysis

The sensitivity analysis led in very small changes in the results, indicating that the SD imputation performed does not significantly affect the results and that the subsequent findings are reliable. The results of the sensitivity analysis are presented in the supplement.

## Discussion

The main findings were that DAA with TT had higher intraoperative blood loss and lower Hb levels three days postoperatively. DAA on a standard operating table had a higher periprosthetic fracture rate. There were no other differences in outcomes between the two groups. By including RCTs and using only high-quality statistical methods, we believe this is the best available evidence on the use of TT in DAA.

There are no relevant primary studies directly comparing DAA on a standard operating table to DAA on a TT, apart from a few non-randomized studies [76–78]. However, the only systematic review that addresses the role of TT in DAA [14] has some severe limitations. In their 2020 systematic review, Sarraj et al. did not perform a classical meta-analysis of the extracted data that could reveal differences in the effect of both surgical techniques. Moreover, they included several studies of lower quality [14]. Furthermore, there is a meta-analysis on DAA with a different study focus that additionally examined the TT influence in a subgroup meta-analysis [8]. The severe limitation here is that there were only four primary studies included in this subgroup meta-analysis with an overall small sample size [8].

Intraoperative blood loss in THA through DAA with TT ranged from 133.7 to 690 mL with an average of 479.1 mL. Intraoperative blood loss in THA through DAA on a standard operating table ranged from 359.7 to 1344.0 mL with an average of 393.2 mL. DAA with TT had a 102.33 mL higher intraoperative blood loss compared with DAA on a standard operating table. The Hb 3 days postoperatively in THA through DAA with TT was 5.4 mmol/L. The Hb three days postoperatively in THA through DAA on a standard operating table ranged from 5.9 to 7.0 mmol/L with an average of 6.5 mL in THA through DAA on a standard operating table. DAA with TT had a 0.60 mmol/L lower Hb three days postoperatively compared with DAA on a standard operating table.

In high-quality studies on this topic, great importance is attached to the consideration of hidden blood loss. This can be estimated well using meaningful serum biomarkers such as Hb. When interpreting the results of this network meta-analysis, it must be emphasized immediately that the outcome parameters Hb one day and Hb two days postoperatively did not show any significant differences. Furthermore, the postoperative drainage volume could not be taken into account in the RCTs due to a lack of primary data or a lack of practical application of postoperative drainage systems.

Information on intraoperative blood loss in DAA with TT was collected from 8 RCTs [33, 34, 44, 45, 47, 50, 63, 64] with overall 471 patients. The results of the individual RCTs do not show any major outliers and appear to be rather uniform (133.7 – 690.0 mL). Information on intraoperative blood loss in DAA without TT was collected from nine RCTs [37, 41, 57, 60, 61, 69, 71, 73, 75] with overall 441 patients. When analyzing the individual RCTs, the excessively high blood loss in the RCT by D'Arrigo et al. [41] is immediately noticeable. Apart from this RCT, the other 8 RCTs [37, 57, 60, 61, 69, 71, 73, 75] do not show any significant outliers (range 359.7–444.4 mL). The mean blood loss would be significantly lower if the RCT by D'Arrigo et al. [41] is omitted. A closer look at the RCT by D’Arrigo et al. [41] also reveals no explanation for the high mean blood loss. However, it is noticeable that the blood loss in the control group of this RCT, which corresponds to the common comparator group of our network meta-analysis, also appears to be excessively high. The RCT by D’Arrigo et al. [41] distorts the blood loss results to the disadvantage of the DAA without TT group. Omitting the distorting RCT by D'Arrigo et al. [41] would show an even clearer and larger difference between DAA with TT and DAA without TT than the difference that was found in the present network meta-analysis.

Information on Hb three days postoperatively in DAA with TT was collected from one sinlge RCT [64], which must be highlighted as a shortcoming in the reliability of the results. However, this RCT [64] provided information on 61 THA patients, which is not a moderate sample size. Information on Hb 3 days postoperatively in DAA without TT was collected from five RCTs [51, 54, 69, 71, 75] with overall 355 patients. The results of the individual RCTs do not show any major outliers and appear to be rather uniform (range: 5.9 – 7.0 mmol/L).

There is no indication in the literature as to what blood loss difference represents a minimal clinically important difference. Nevertheless, the observed difference of approximately 100 m/L appears to be meaningful. The exposure of the surgical site in DAA with TT and DAA on a standard operating table is quite different despite the identical surgical approach, but due to the different surgical technique. Whether this leads to a different exposure of potentially haemorrhaging vessels with more difficult haemostasis in DAA with TT, we can only speculate at present. This result is interesting and should be investigated further in new studies, comparing DAA with TT with DAA on a standard table with a focus on the blood loss. The other analyzed parameters of surgical, radiological and functional outcomes showed no significant differences.

The periprosthetic fracture rate was 0.63% in THA through DAA with TT and 0.64% in THA through DAA on a standard operating table. DAA with TT had a 0.15 lower periprosthetic fracture OR compared with DAA without TT. A total of 15 RCTs [33–36, 39, 43–45, 50, 53, 55, 58, 63, 65, 66] with overall 633 patients reported information on periprosthetic fracture rate in DAA with TT. Of these 633 patient cases, only four cases (0.63%) had periprosthetic fractures. These four cases were reported in two RCTs [39, 65] with overall 62 patients. Cheng et al. [39] reported two periprosthetic fractures in their RCT. The first was an intraoperative femoral perforation during femoral broaching. It was treated with protected weight bearing for six weeks. The second was an avulsion fracture of the greater trochanter, which was treated conservatively. Taunton et al. reported two cases of intraoperative fractures of the calcar in their RCT [65]. They were treated with intraoperative cerclage wiring.

A total of 18 RCTs [37, 40, 41, 46, 48, 49, 52, 54, 56, 59–62, 68, 70–73, 75] with overall 1088 patients reported information on periprosthetic fracture rate in DAA on standard operating table. Of these 1,088 patient cases, only seven cases (0.64%) resulted in periprosthetic fractures. These seven cases were reported in 6 RCTs [41, 52, 56, 68, 71, 73] with a total of 357 patients. In their RCT [41], D’Arrigo et al. reported one avulsion fracture of greater trochanter and one proximal femoral fracture. Mjaaland et al. reported in their RCT [52] an avulsion fracture of the greater trochanter, which was fixed with a cable wire during the primary operation. In their RCT [56], Nistor et al. reported an avulsion fracture of the greater trochanter, which did not require fixation. The same complication was observed in the RCTs by Vandeputte et al. [68], Xiao et al. [71], and Zhao et al. [73].

When interpreting the periprosthetic fracture results, the moderate number of cases is striking, which calls into question the reliability of the results, but cannot invalidate them. The results are statistically significant. One possible explanation for the higher rate of greater trochanter avulsion fractures in DAA on a standard operating table is the need to lever with the retractor in order to obtain an overview of the surgical site. This leverage effect on the greater trochanter is not necessary in DAA with TT, as exposure of the surgical site is achieved by traction and rotation movements with the foot holder. A possible solution for DAA on a standard operating table to reduce the risk of periprosthetic fractures may be to reduce the leverage of the retractor on the greater trochanter by improving the release.

It is known that the femoral cutaneous nerve (LFCN) palsy is a typical complication of DAA due to the nature of surgical approach. Our meta-data on LFCN palsy rate were collected from 34 RCTs [33–37, 39–41, 43–46, 48–50, 52–56, 58–63, 65, 66, 68, 70–73, 75], which reported a total of 62 LFCN palsy events in 1,721 THA patients. Here, it is important to recognize from the present network meta-analysis that the use of TT in DAA has no effect on the LFCN palsy rate. The other complication rates analyzed and the overall complication rate also showed no significant differences.

The interpretation of the results is very important for our daily orthopaedic practice. The difference of approximately 100 ml less intraoperative blood loss with DAATHA on a standard operating table does not appear to justify a change in surgical technique. There are enough known measures such as tranexamic acid application, heat preservation etc. that can minimize blood loss. However, the potentially higher blood loss should be considered by surgeons and proponents of DAA THA with TT. The higher rate of periprosthetic fractures in DAA on a standard operating table, and more specifically of avulsion fracture pf the greater trochanteric, probably due to the leverage provided by the retractor, is a very interesting and valuable finding. This should definitely be investigated further. If the meta-data of the present network meta-analysis is confirmed, it would provide a solid argument for the use of the TT.

Several limitations apply to this network meta-analysis: (1) Due to the lack of RCTs that directly compare DAA THA with TT with DAA THA on a standard operating table, an indirect comparison of both techniques was performed. (2) Due to insufficient data, some outcome parameters have a low number of DAA THA patient cases. (3) As usual for similar studies, there are also possible confounding factors that could distort the results in some way (e.g. the surgeon operating skills, bone cement use, different implants types). (4) For some of the analyzed outcome parameters, the heterogeneity and publication bias of the included RCTs call into question the reliability of the results.

## Conclusion

Based on our findings and taking into account the study limitations, we recommend that particular attention be paid to the risk of periprosthetic fracture in DAA on a standard operating table and blood loss in DAA with TT. Reducing the leverage of the retractor on the greater trochanter by improving the release may be a possible solution. Since numerous other surgical, radiological, functional outcome parameters and other complication rates studied showed no significant difference between DAA on a standard operating table and DAA with TT, no recommendation for a change in surgical technique seems justified.

### Supplementary Information

Supplementary file 1 

## Data Availability

Raw data extraction sheet is available in supplement.

## Abbreviations

CI: Confidence interval
DAA: Direct anterior approach
DVT: Deep vein thrombosis
HHS: Harris Hip Score
ITT: Intention to treat
JBI: Joanna Briggs Institute
LFCN: Lateral femoral cutaneous nerve
SMD: Standardized mean difference
OR: Odds ratio
PE: Pulmonary embolism
PP: Per protocol
PRISMA: Preferred Reporting Items for Systematic Reviews and Meta-Analyses
RCTs: Randomized controlled studies
RoB: Risk of bias
THA: Total hip arthroplasty
TT: Traction table
SD: Standard deviation
