# Supplementary figures and images for: Indirect comparisons of traction table versus standard table in total hip arthroplasty through direct anterior approach: a systematic review and frequentist network meta-analysis
Source: J Orthop Surg Res. 2024 Jun 29;19:384. doi: 10.1186/s13018-024-04852-3 (PMC11218227; doi:10.1186/s13018-024-04852-3)

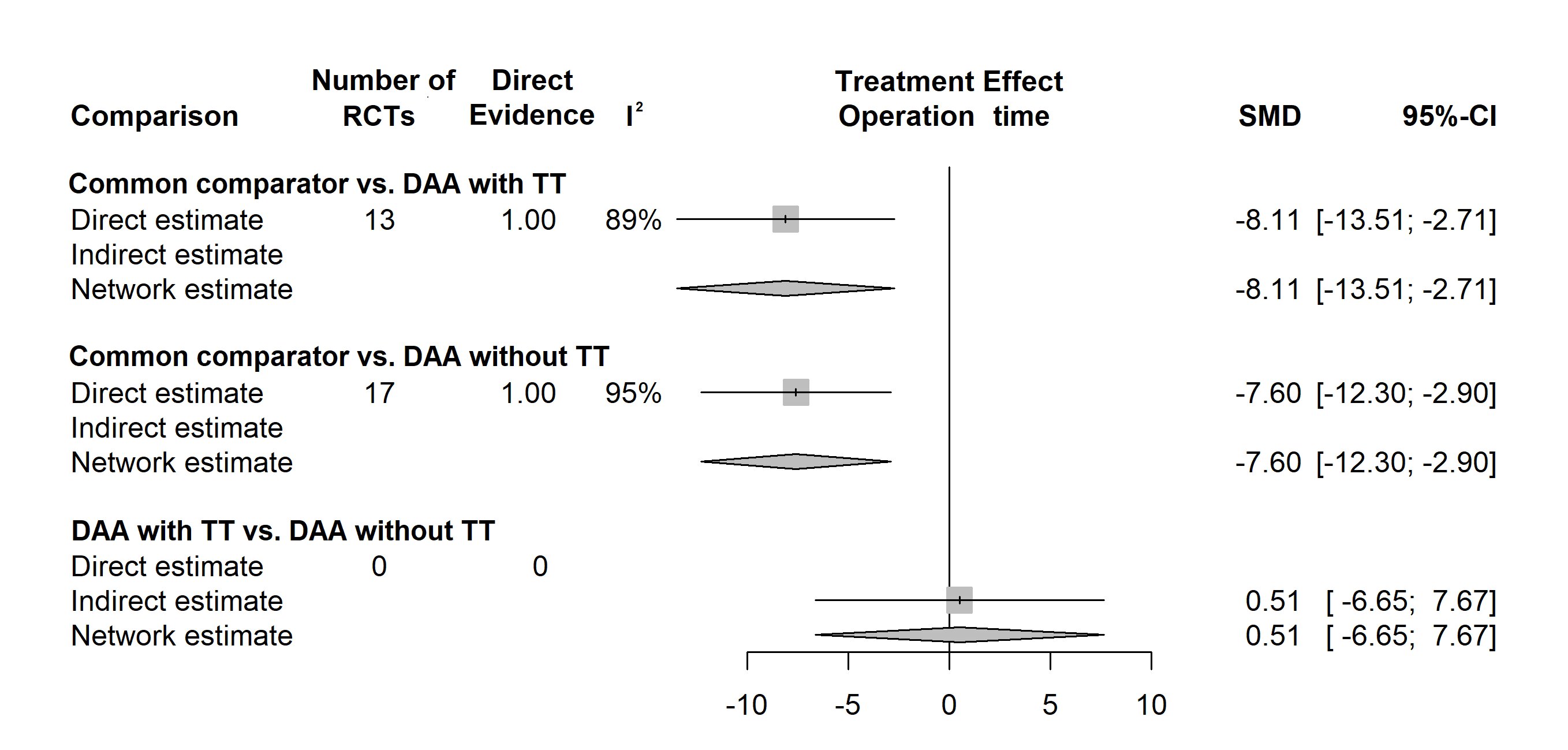

Supplement: Supplementary file 1 [file 13018_2024_4852_MOESM1_ESM.zip › Supplementary/Supplemental Figure 1 - Forest plot Operation Time .jpg]

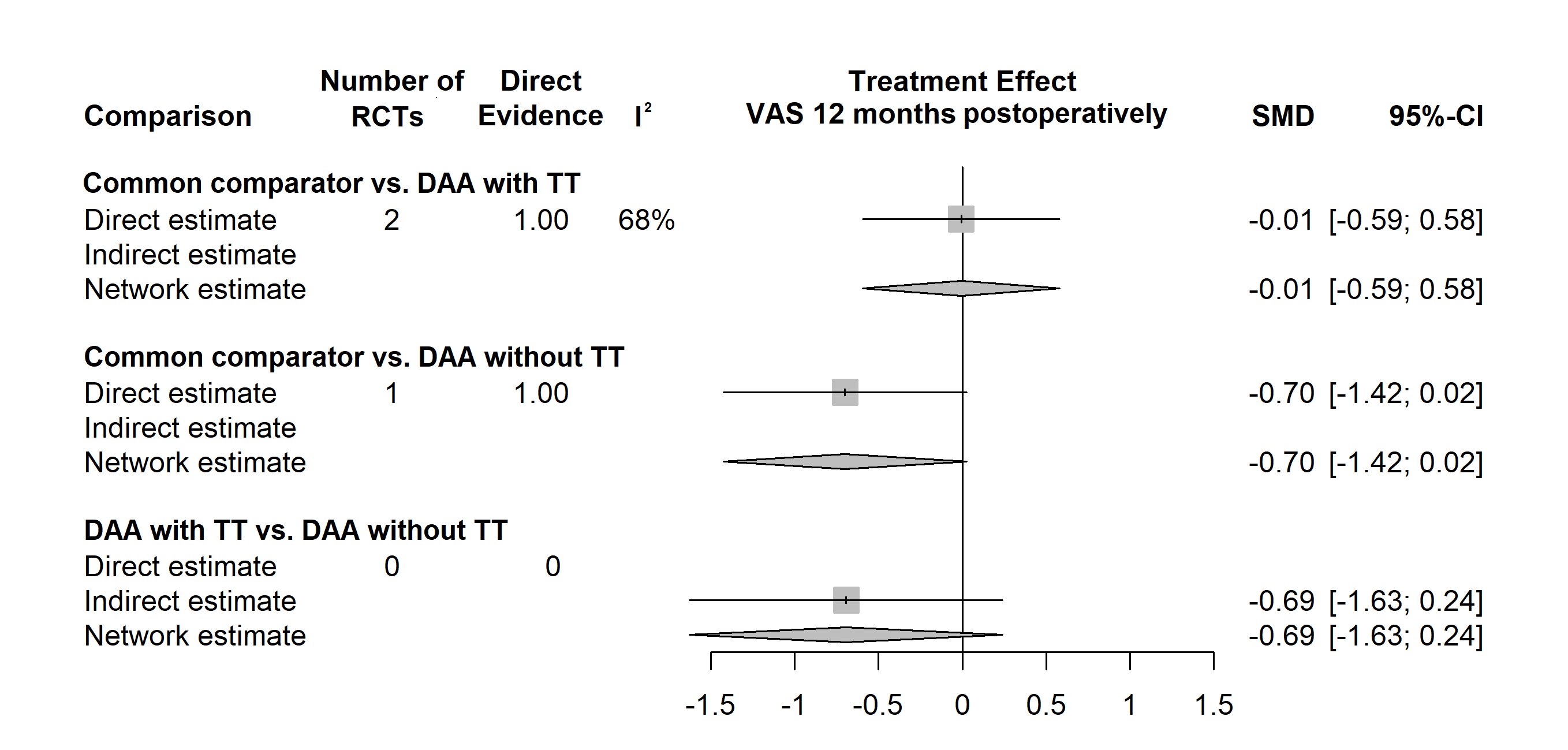

Supplement: Supplementary file 1 [file 13018_2024_4852_MOESM1_ESM.zip › Supplementary/Supplemental Figure 10 - Forest plot VAS 12 months.jpg]

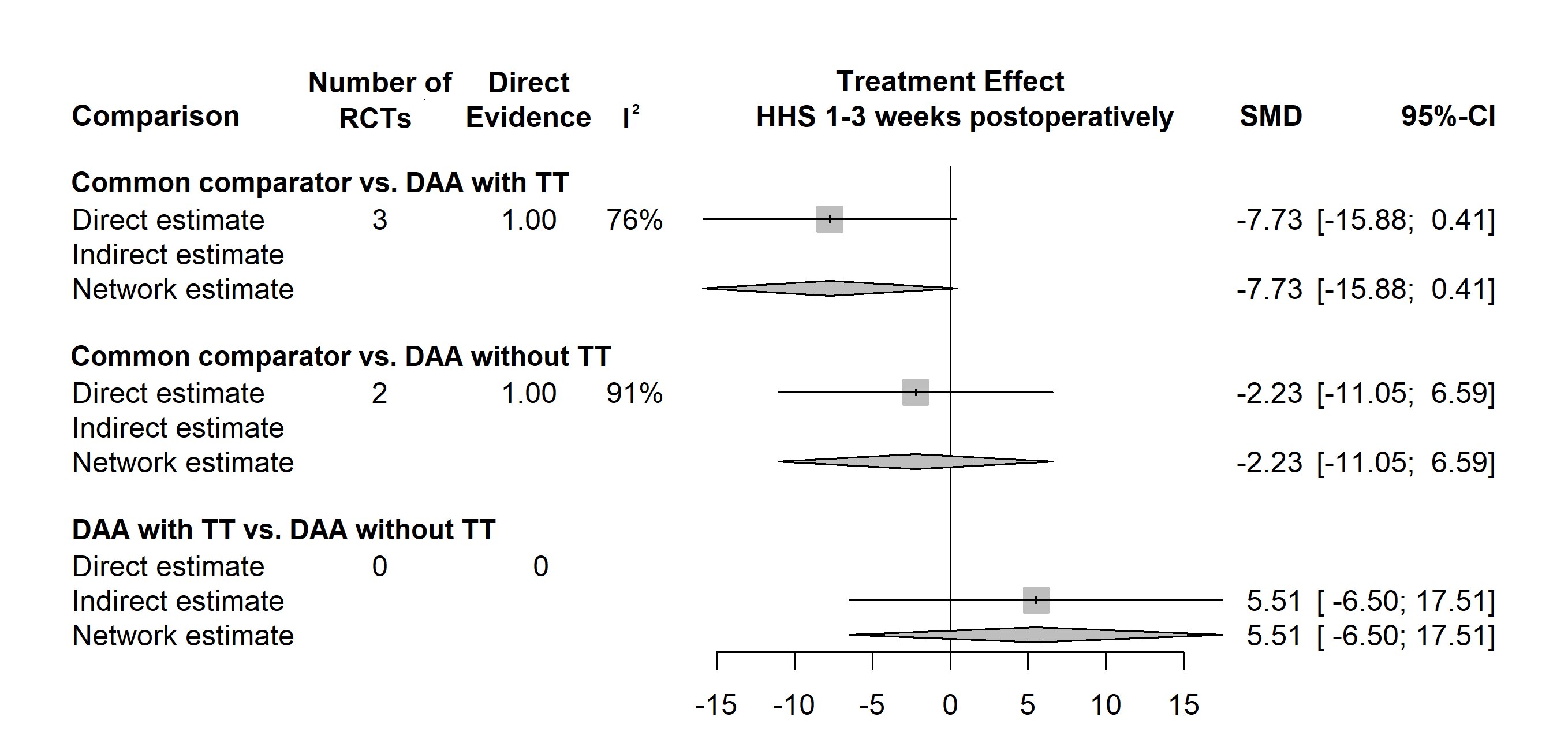

Supplement: Supplementary file 1 [file 13018_2024_4852_MOESM1_ESM.zip › Supplementary/Supplemental Figure 11 - Forest plot HHS 1-3 weeks.jpg]

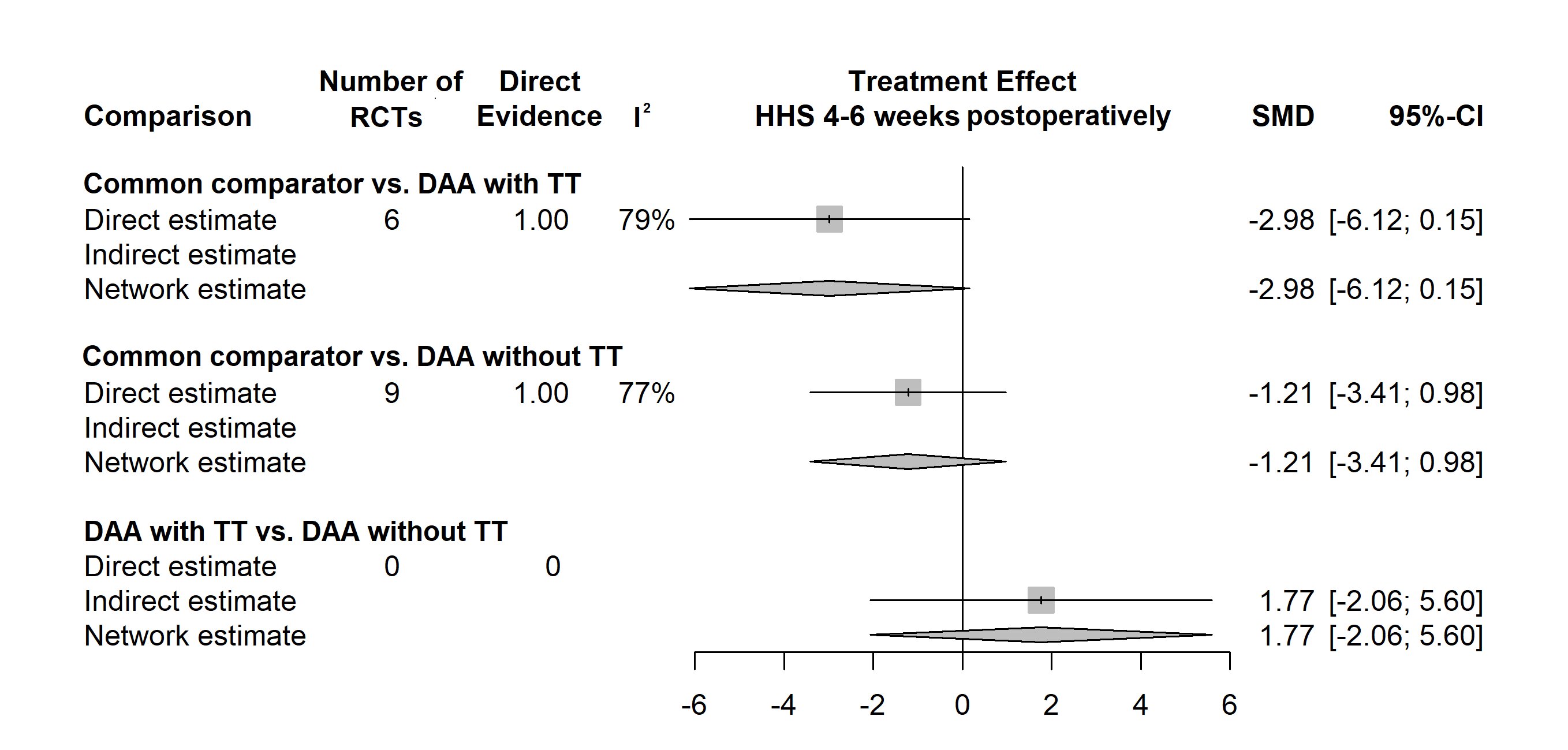

Supplement: Supplementary file 1 [file 13018_2024_4852_MOESM1_ESM.zip › Supplementary/Supplemental Figure 12 - Forest plot HHS 4-6 weeks.jpg]

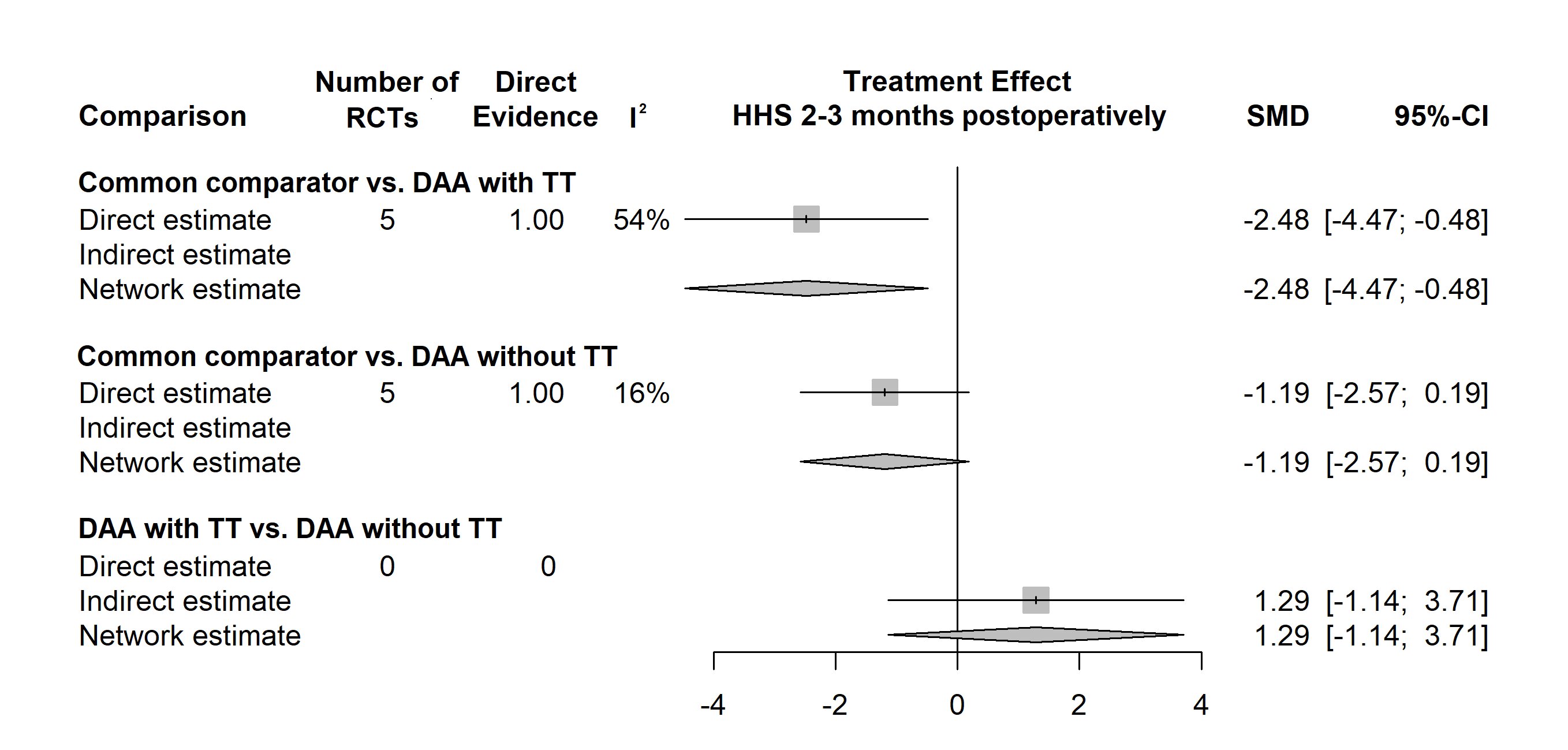

Supplement: Supplementary file 1 [file 13018_2024_4852_MOESM1_ESM.zip › Supplementary/Supplemental Figure 13 - Forest plot HHS 2-3 months.jpg]

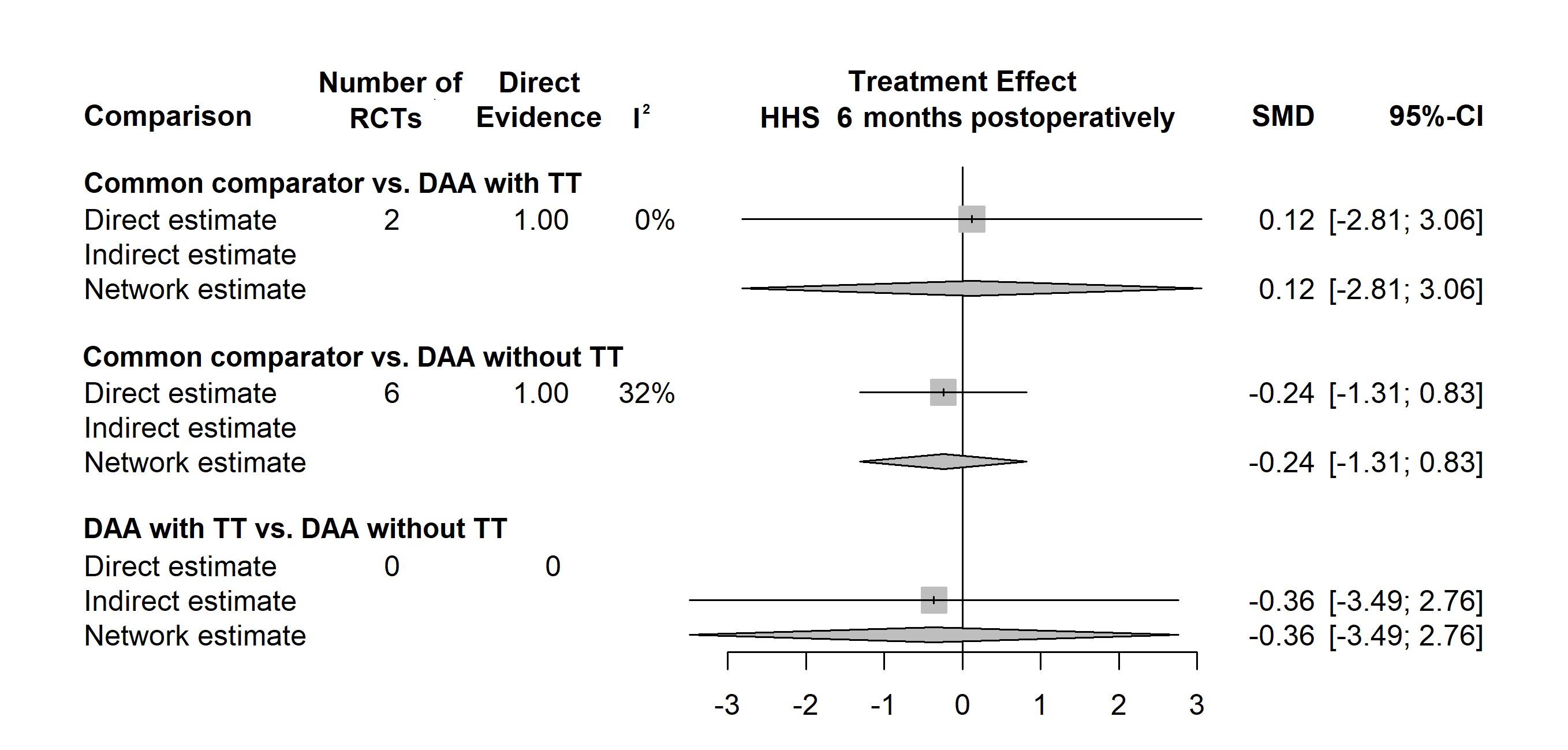

Supplement: Supplementary file 1 [file 13018_2024_4852_MOESM1_ESM.zip › Supplementary/Supplemental Figure 14 - Forest plot HHS 6 Months.jpg]

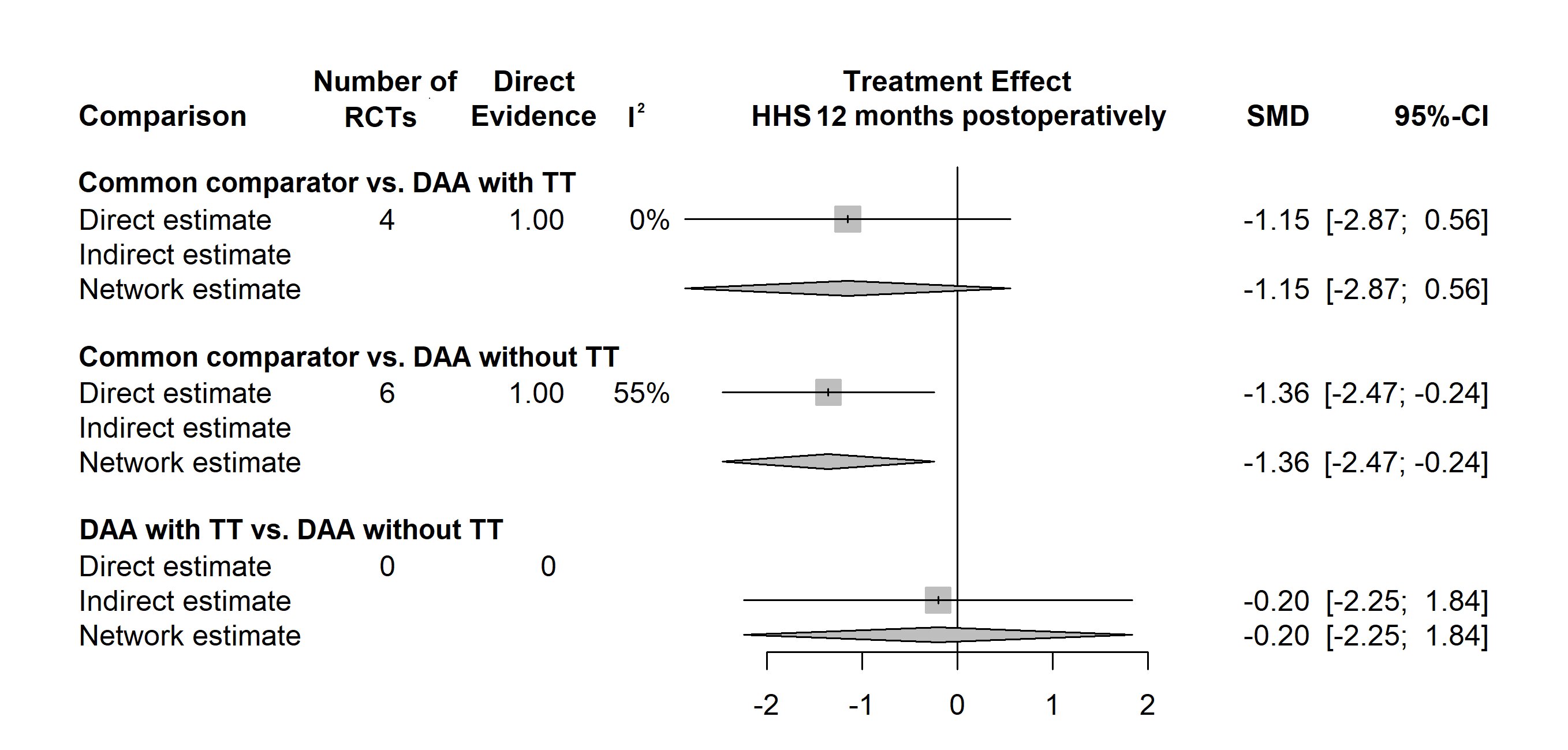

Supplement: Supplementary file 1 [file 13018_2024_4852_MOESM1_ESM.zip › Supplementary/Supplemental Figure 15 - Forest plot HHS 12 Months.jpg]

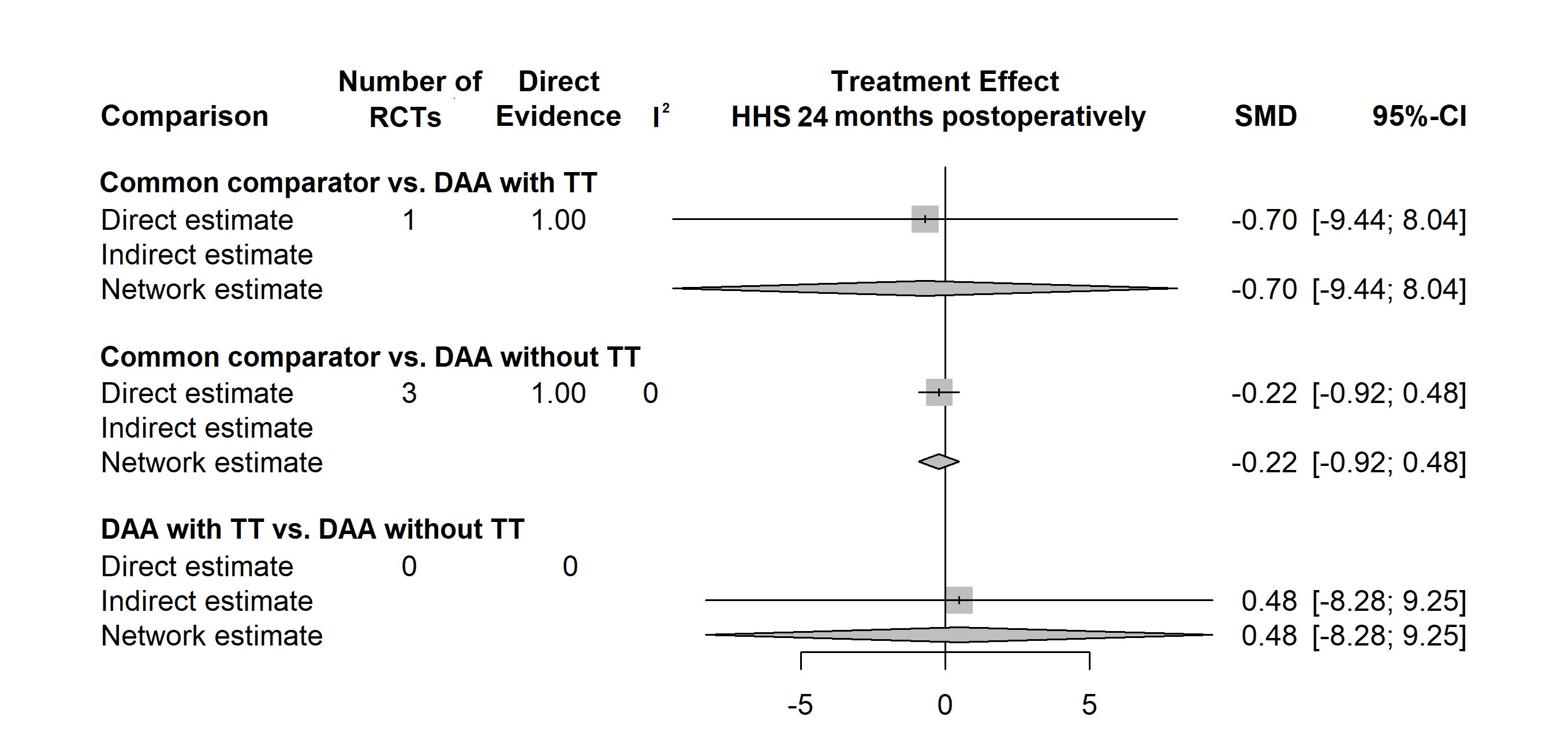

Supplement: Supplementary file 1 [file 13018_2024_4852_MOESM1_ESM.zip › Supplementary/Supplemental Figure 16 - Forest plot HHS 24 Months.jpg]

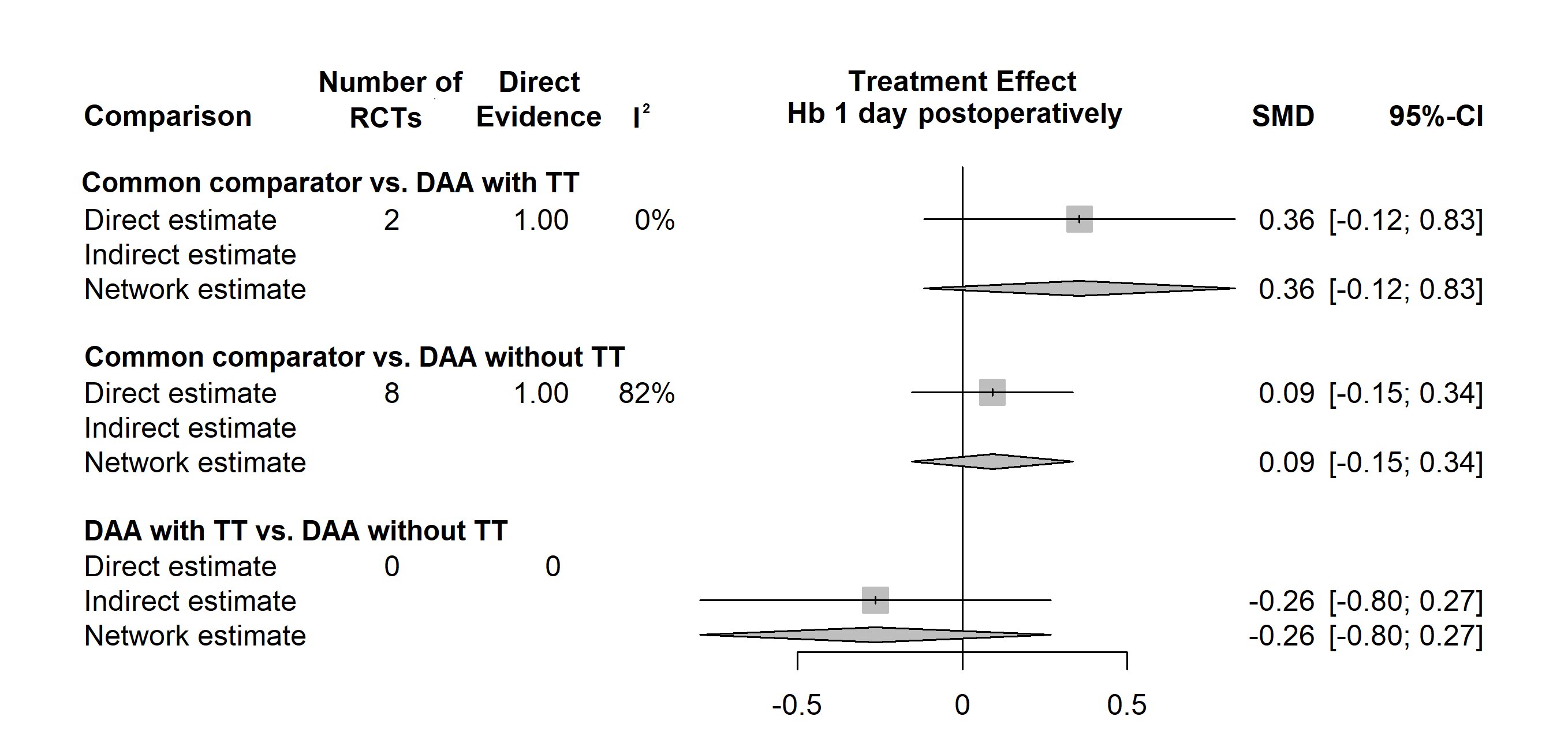

Supplement: Supplementary file 1 [file 13018_2024_4852_MOESM1_ESM.zip › Supplementary/Supplemental Figure 17 - Forest plot Hb 1 day.jpg]

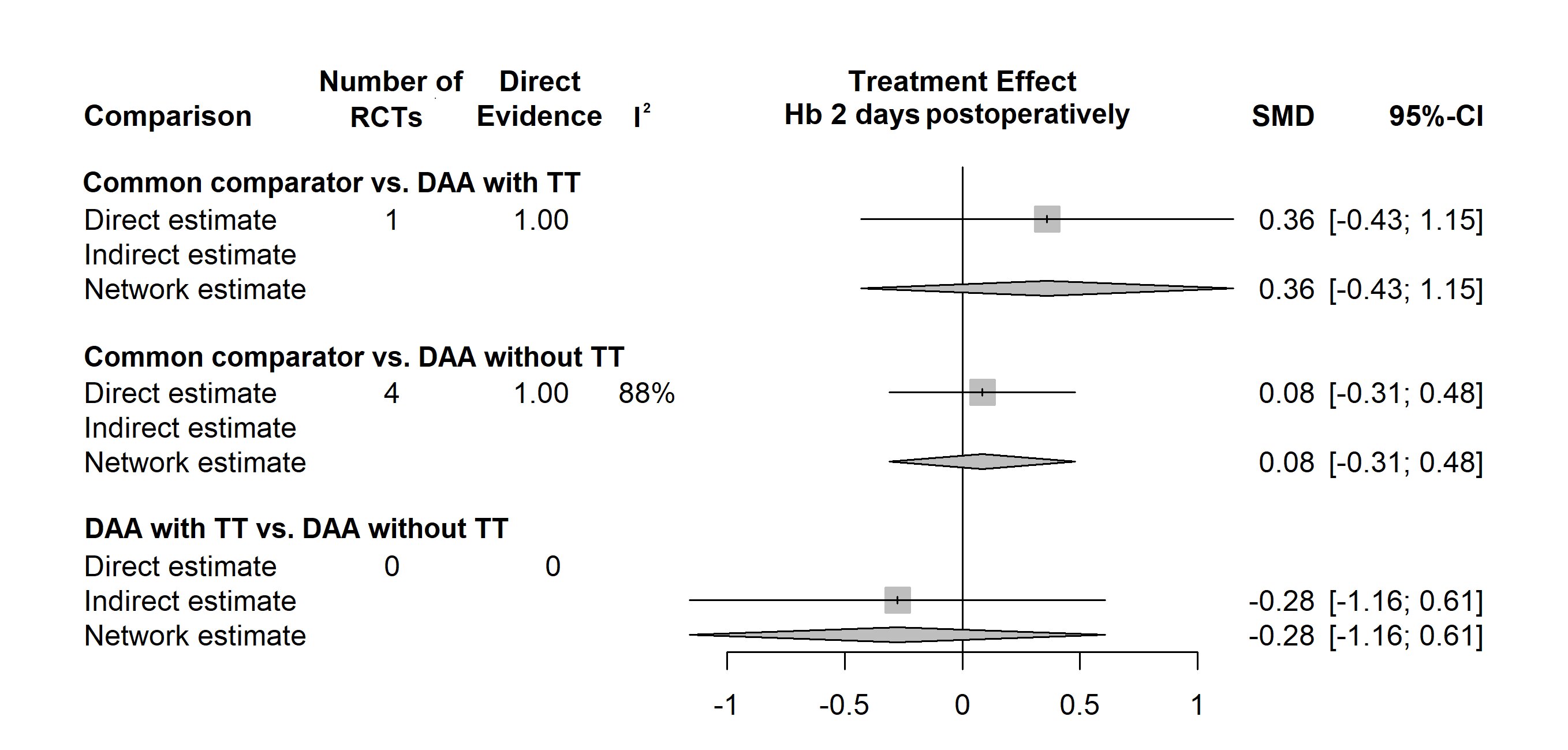

Supplement: Supplementary file 1 [file 13018_2024_4852_MOESM1_ESM.zip › Supplementary/Supplemental Figure 18 - Forest plot Hb 2 days.jpg]

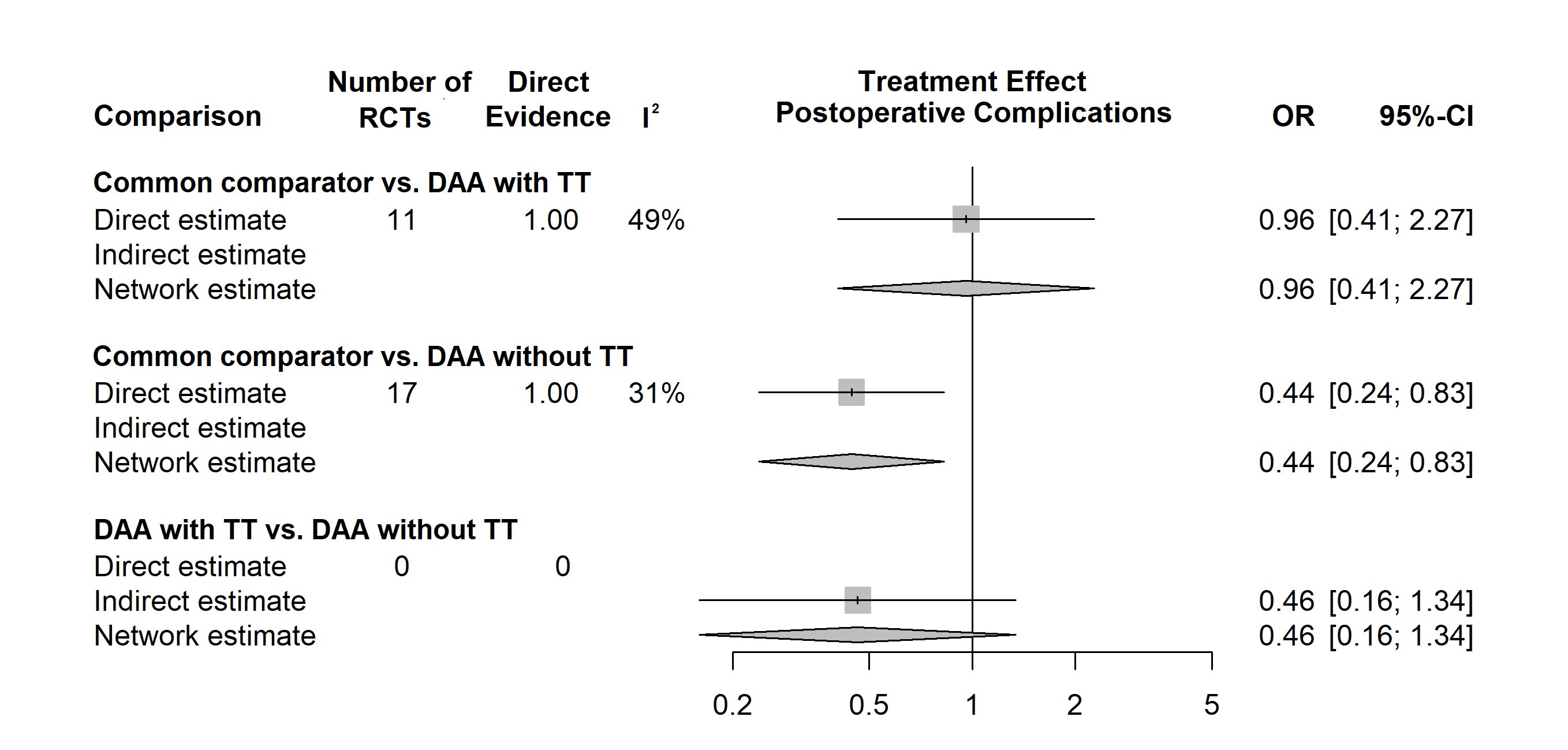

Supplement: Supplementary file 1 [file 13018_2024_4852_MOESM1_ESM.zip › Supplementary/Supplemental Figure 19 - Forest plot Postoperative Complications.jpg]

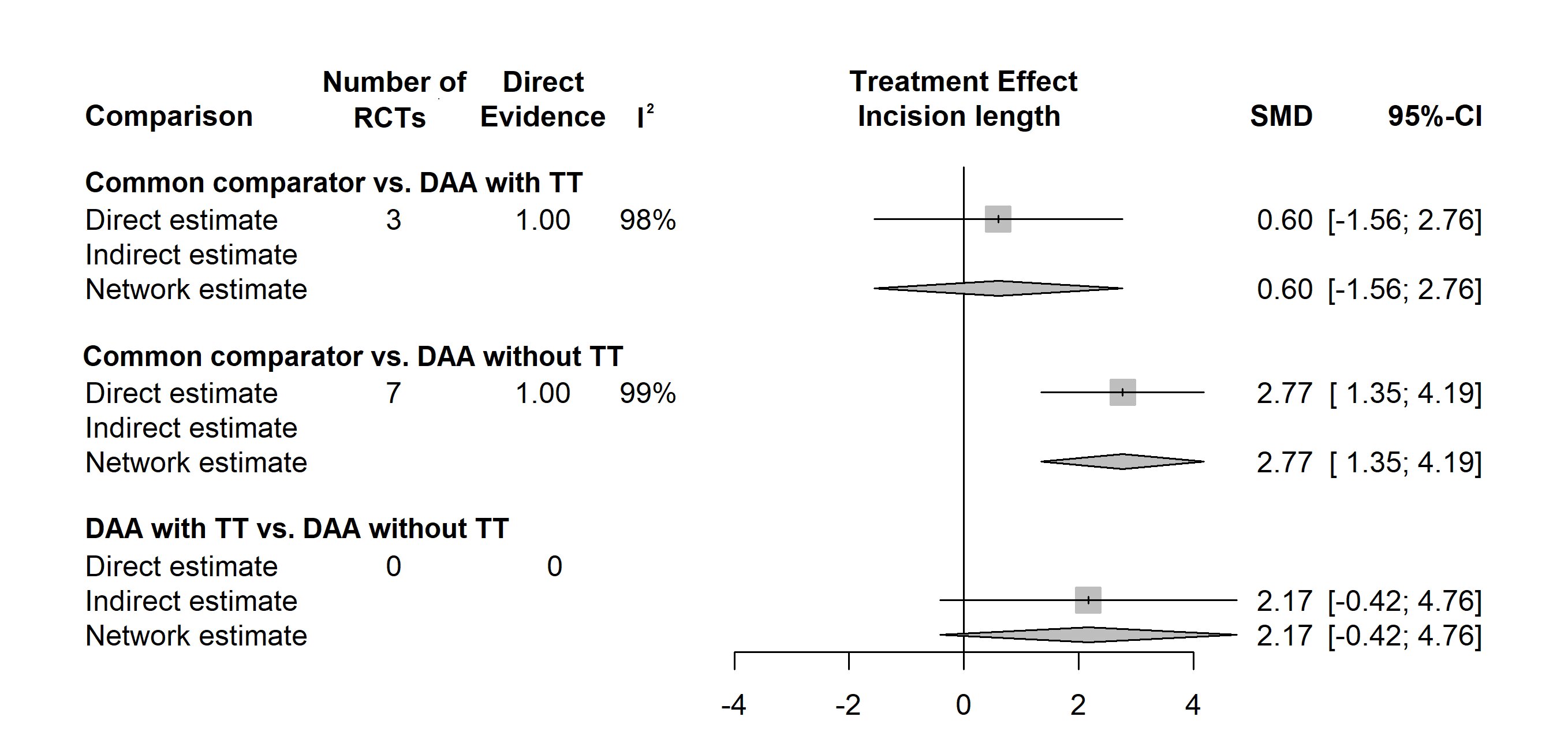

Supplement: Supplementary file 1 [file 13018_2024_4852_MOESM1_ESM.zip › Supplementary/Supplemental Figure 2 - Forest plot Incision length.jpg]

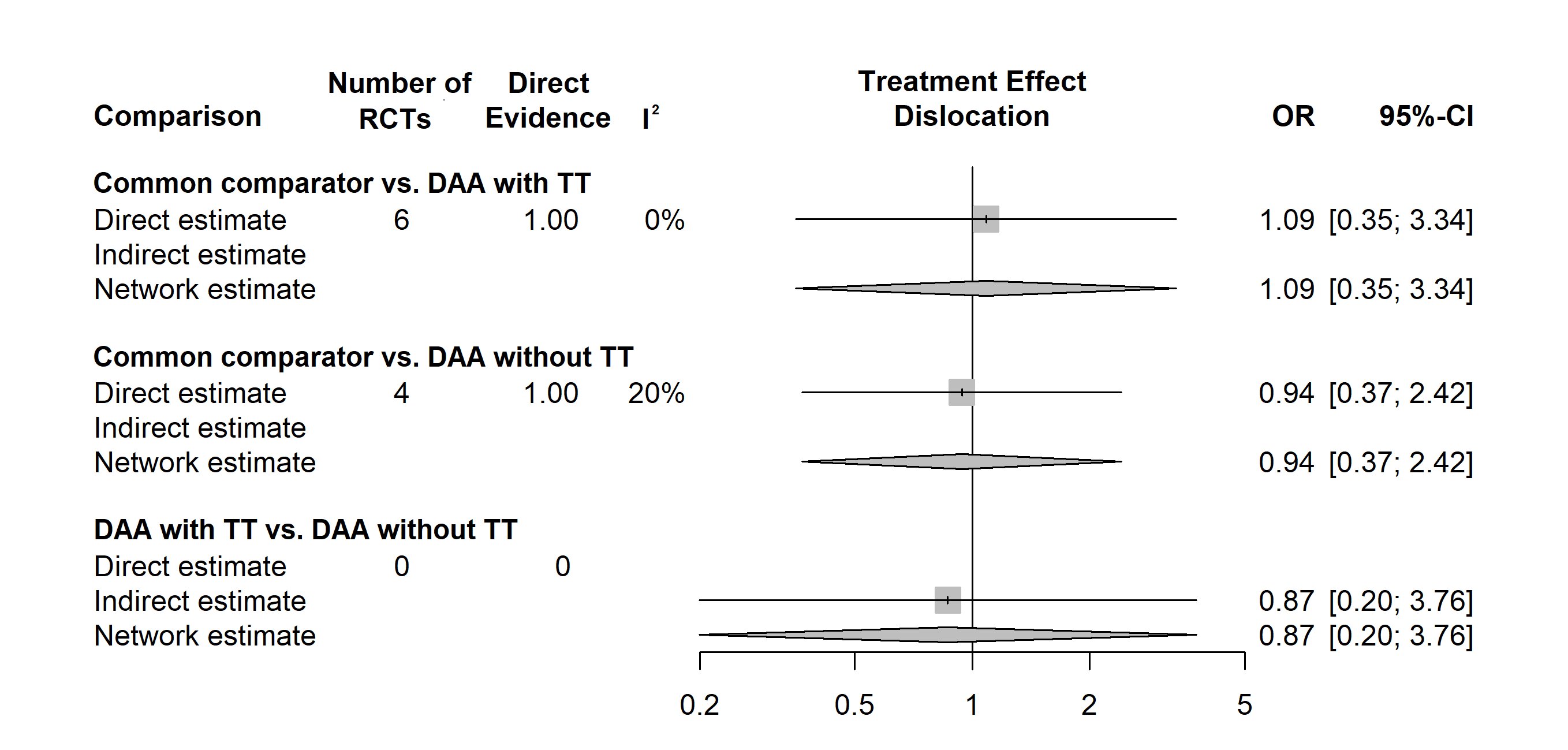

Supplement: Supplementary file 1 [file 13018_2024_4852_MOESM1_ESM.zip › Supplementary/Supplemental Figure 20 - Forest plot Dislocation.jpg]

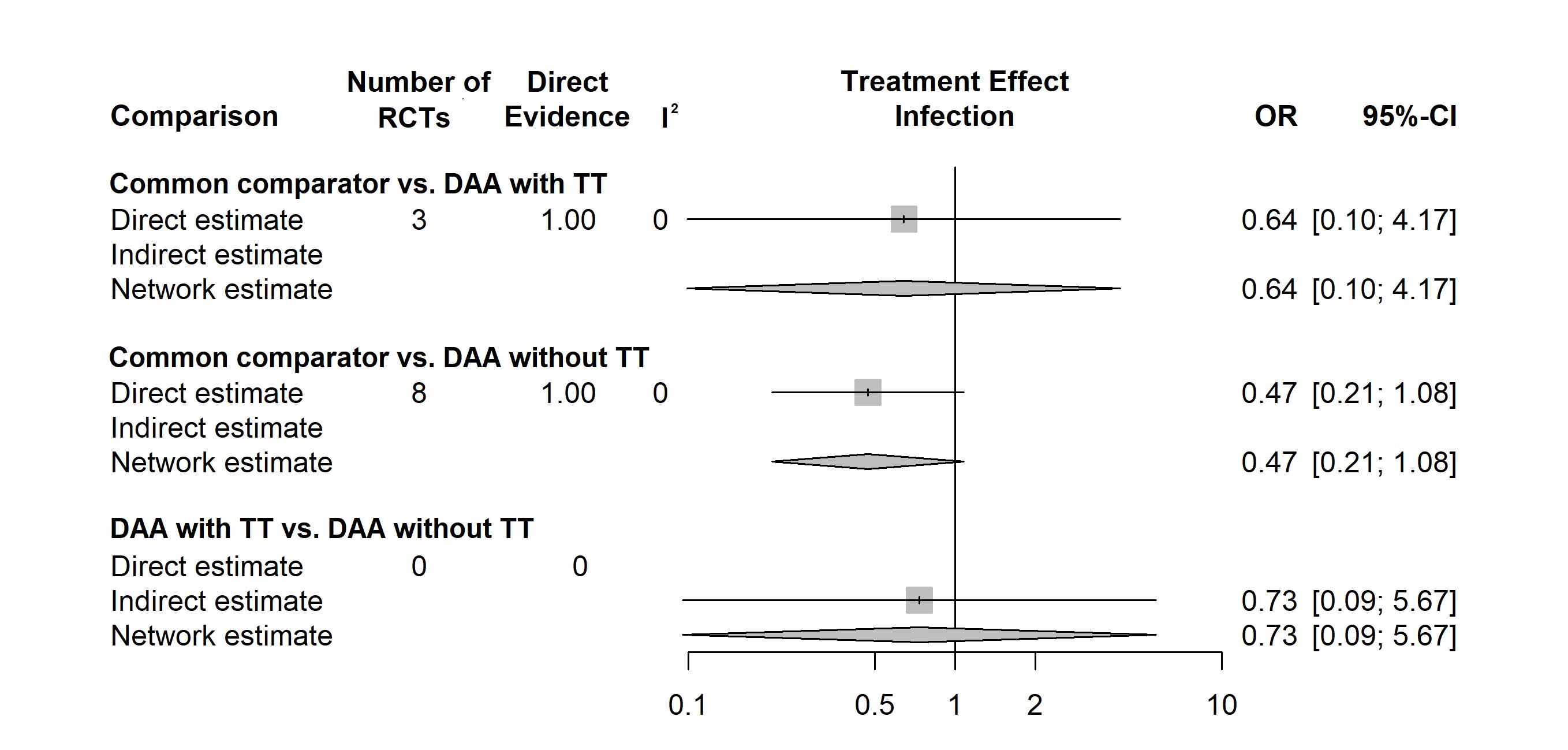

Supplement: Supplementary file 1 [file 13018_2024_4852_MOESM1_ESM.zip › Supplementary/Supplemental Figure 21 - Forest plot Infection.jpg]

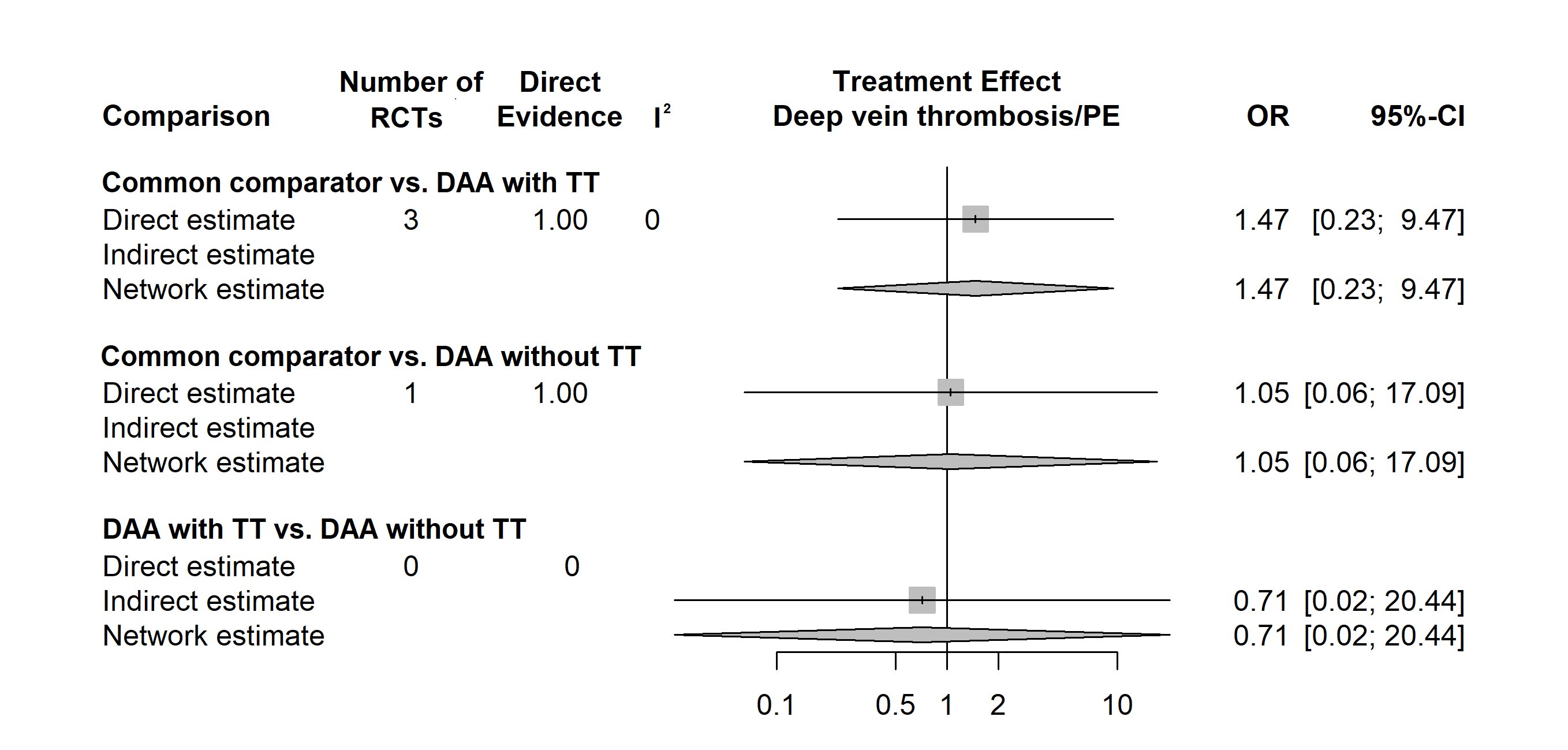

Supplement: Supplementary file 1 [file 13018_2024_4852_MOESM1_ESM.zip › Supplementary/Supplemental Figure 22 - Forest plot DVT PE.jpg]

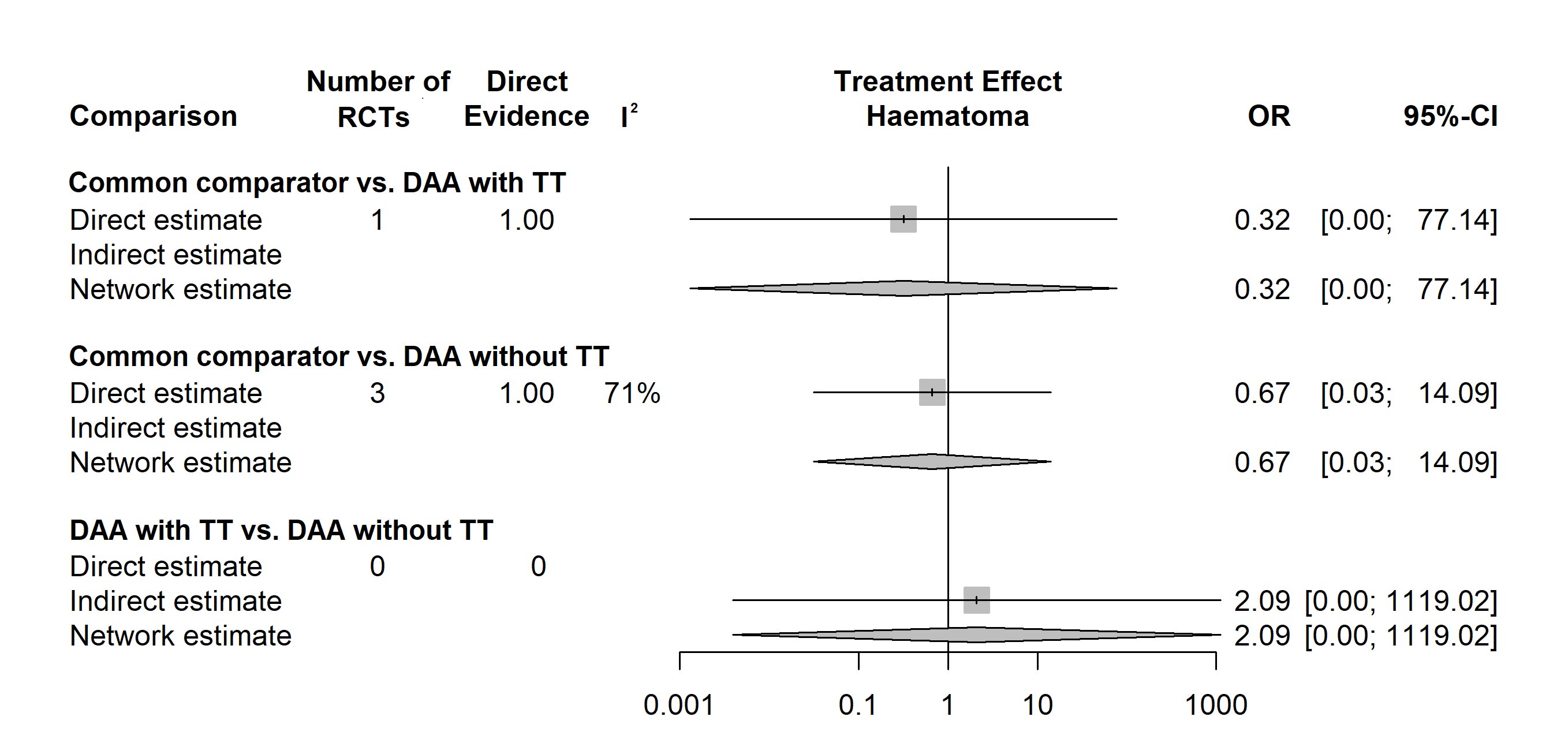

Supplement: Supplementary file 1 [file 13018_2024_4852_MOESM1_ESM.zip › Supplementary/Supplemental Figure 23 - Forest plot Haematoma.jpg]

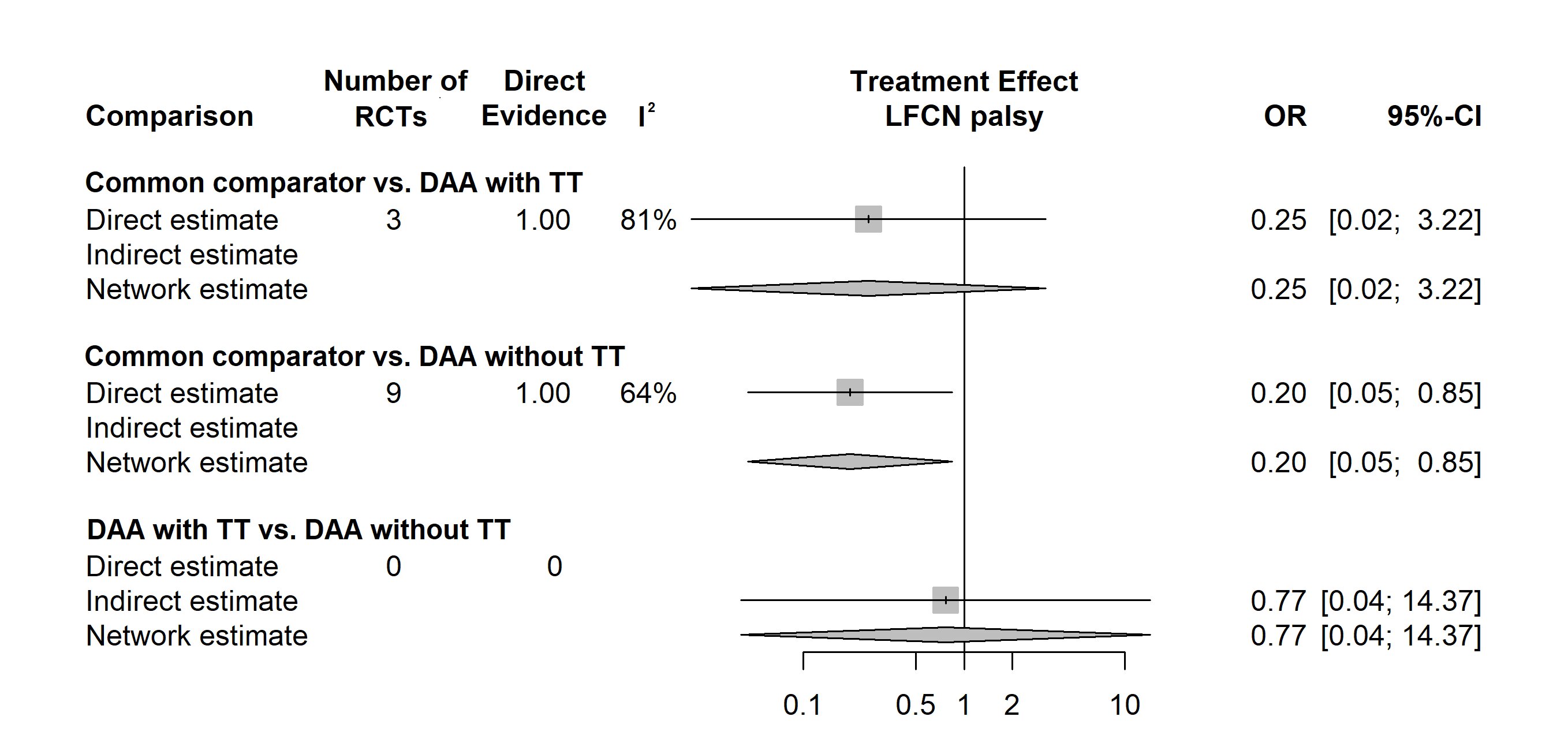

Supplement: Supplementary file 1 [file 13018_2024_4852_MOESM1_ESM.zip › Supplementary/Supplemental Figure 24 - Forest plot LFCN palsy.jpg]

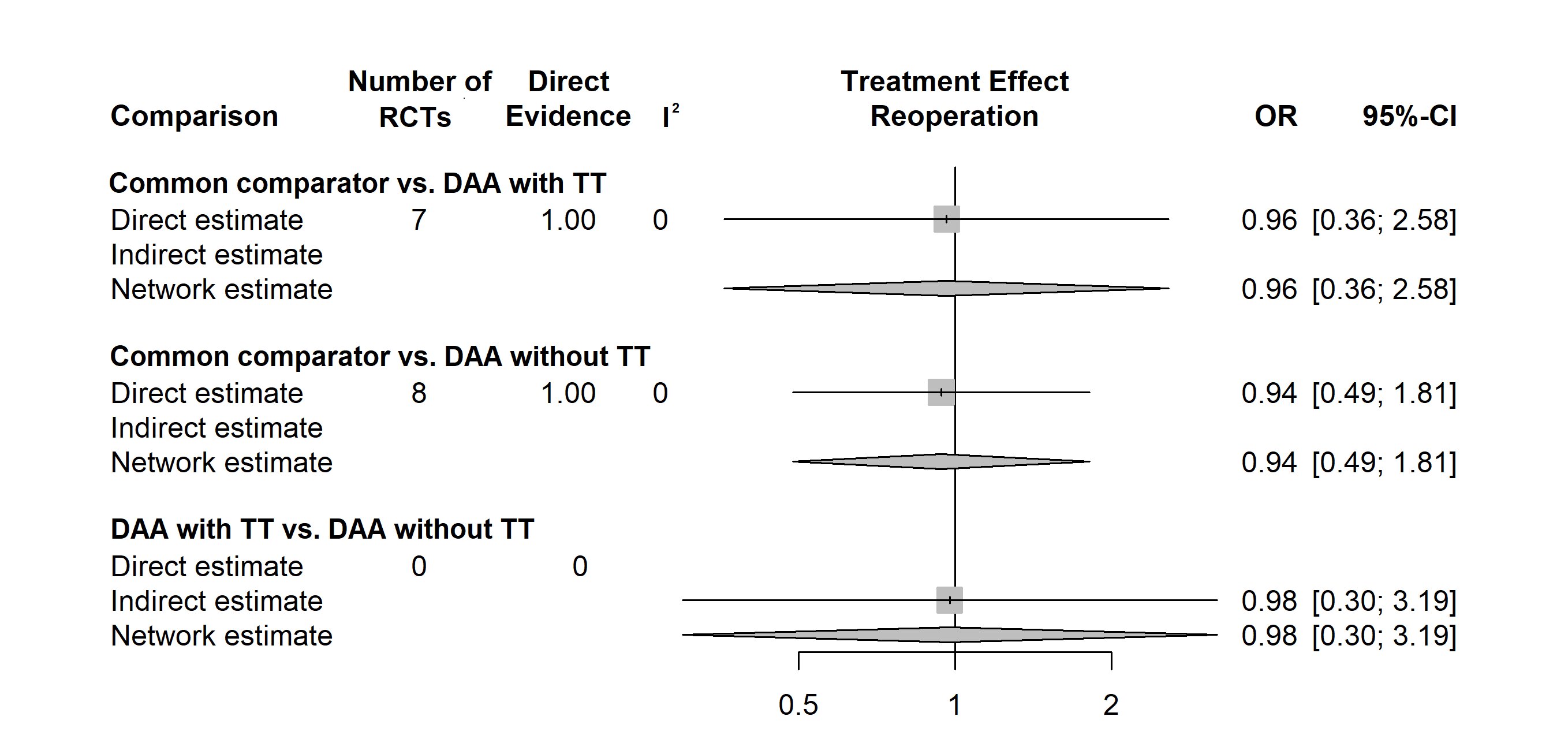

Supplement: Supplementary file 1 [file 13018_2024_4852_MOESM1_ESM.zip › Supplementary/Supplemental Figure 25 - Forest plot Reoperation.jpg]

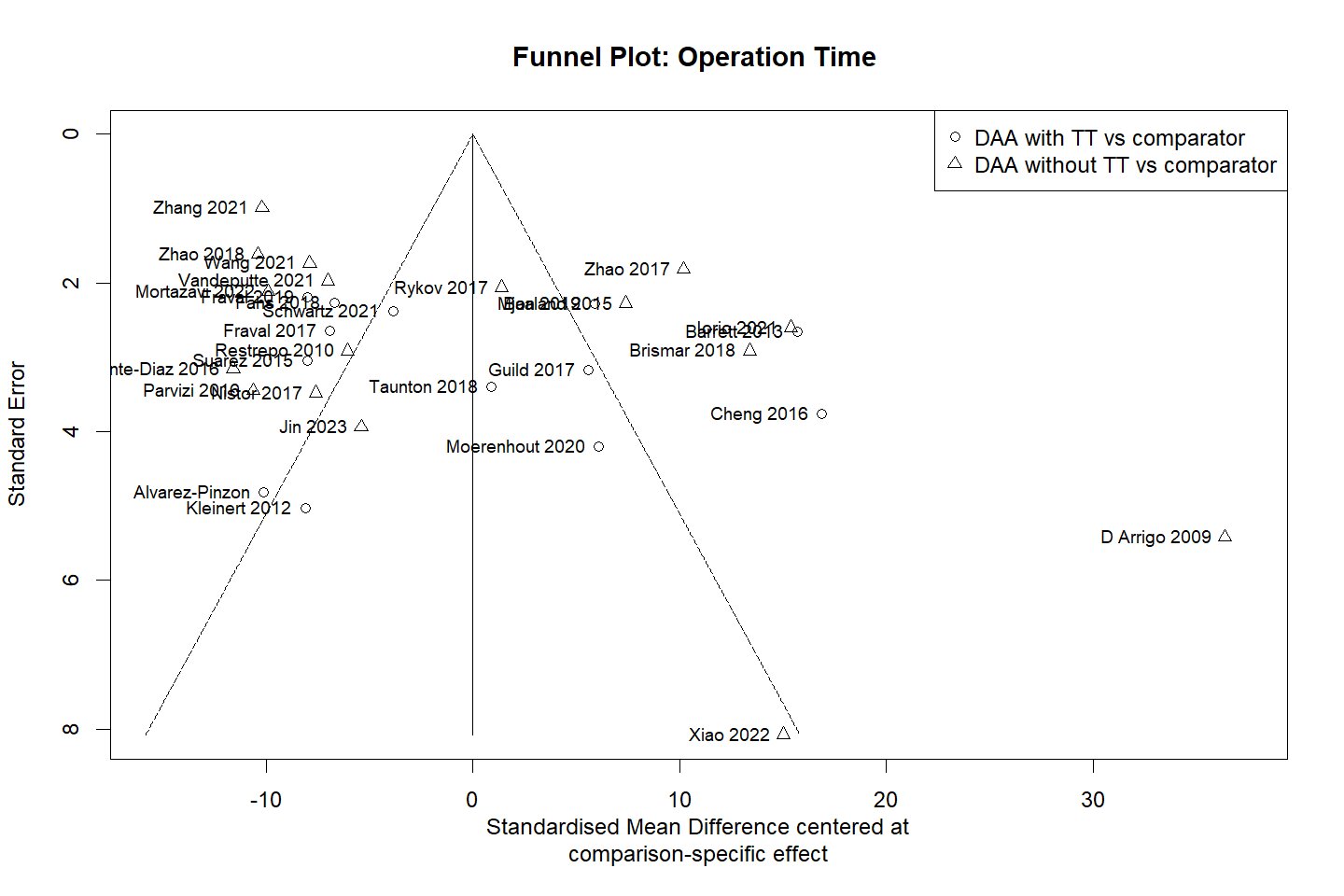

Supplement: Supplementary file 1 [file 13018_2024_4852_MOESM1_ESM.zip › Supplementary/Supplemental Figure 26 - Funnel plot Operation time.jpg]

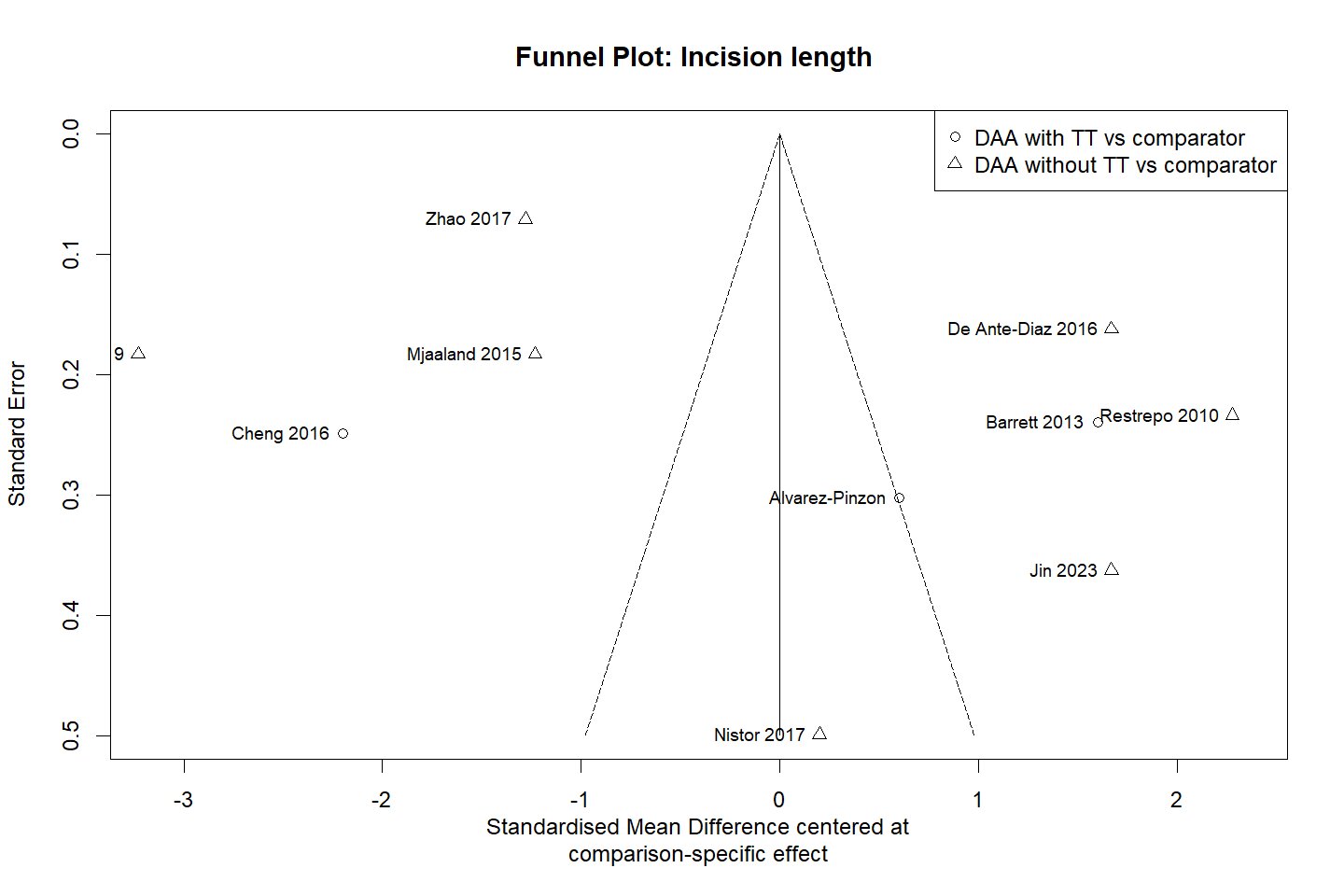

Supplement: Supplementary file 1 [file 13018_2024_4852_MOESM1_ESM.zip › Supplementary/Supplemental Figure 27 - Funnel plot Incision length.jpg]

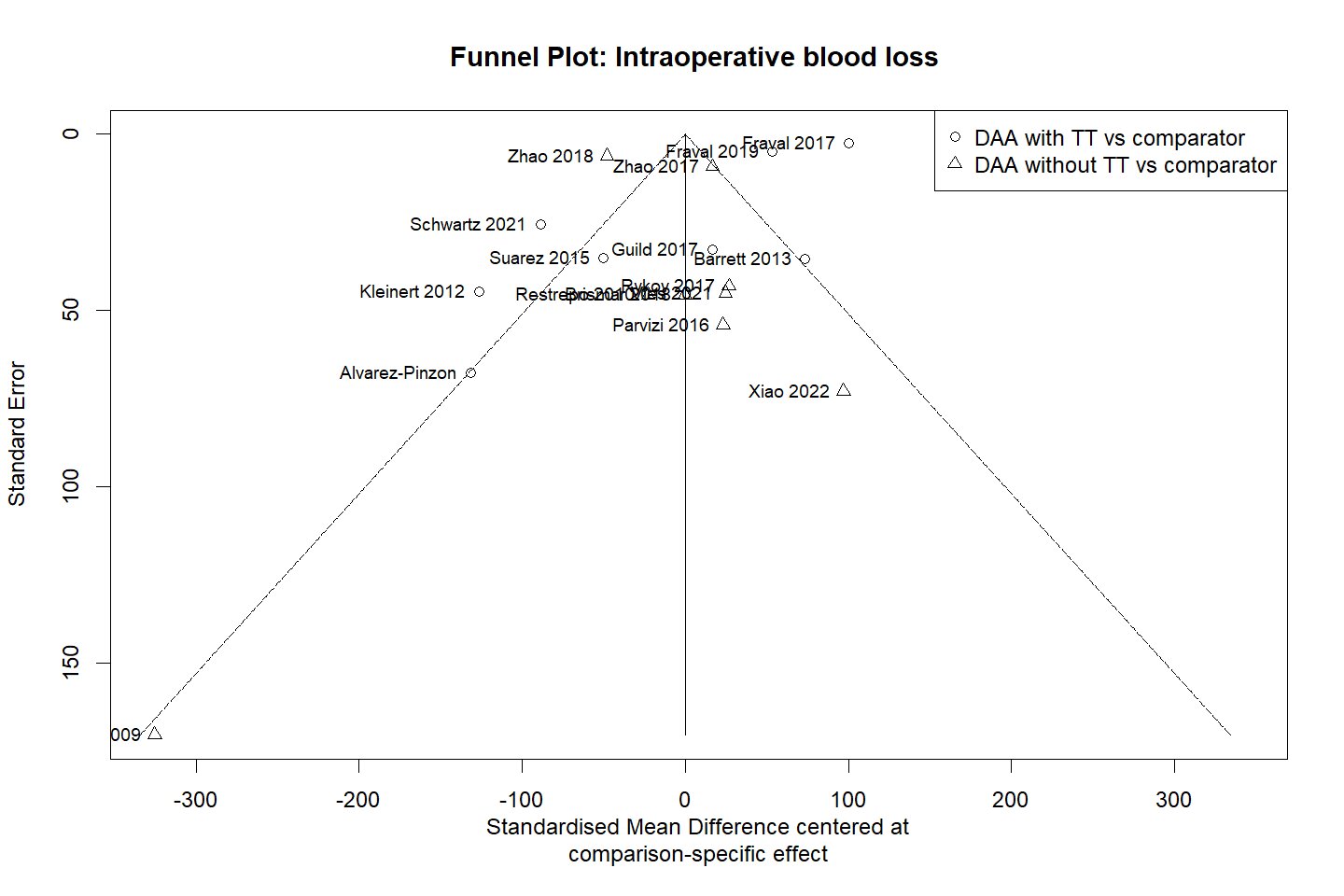

Supplement: Supplementary file 1 [file 13018_2024_4852_MOESM1_ESM.zip › Supplementary/Supplemental Figure 28 - Funnel plot Intraoperative blood loss.jpg]

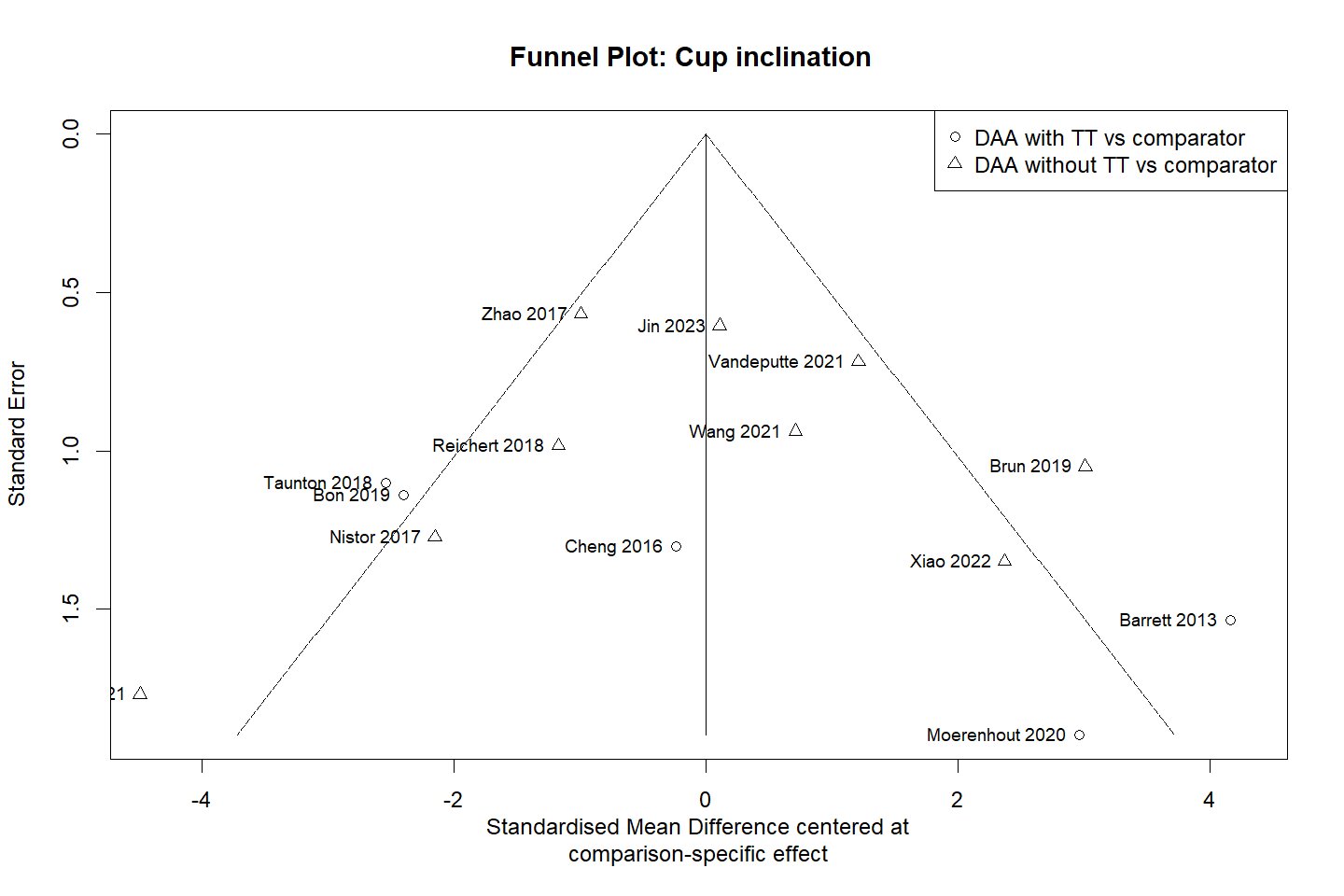

Supplement: Supplementary file 1 [file 13018_2024_4852_MOESM1_ESM.zip › Supplementary/Supplemental Figure 29 - Funnel plot Acetabular cup inclination.jpg]

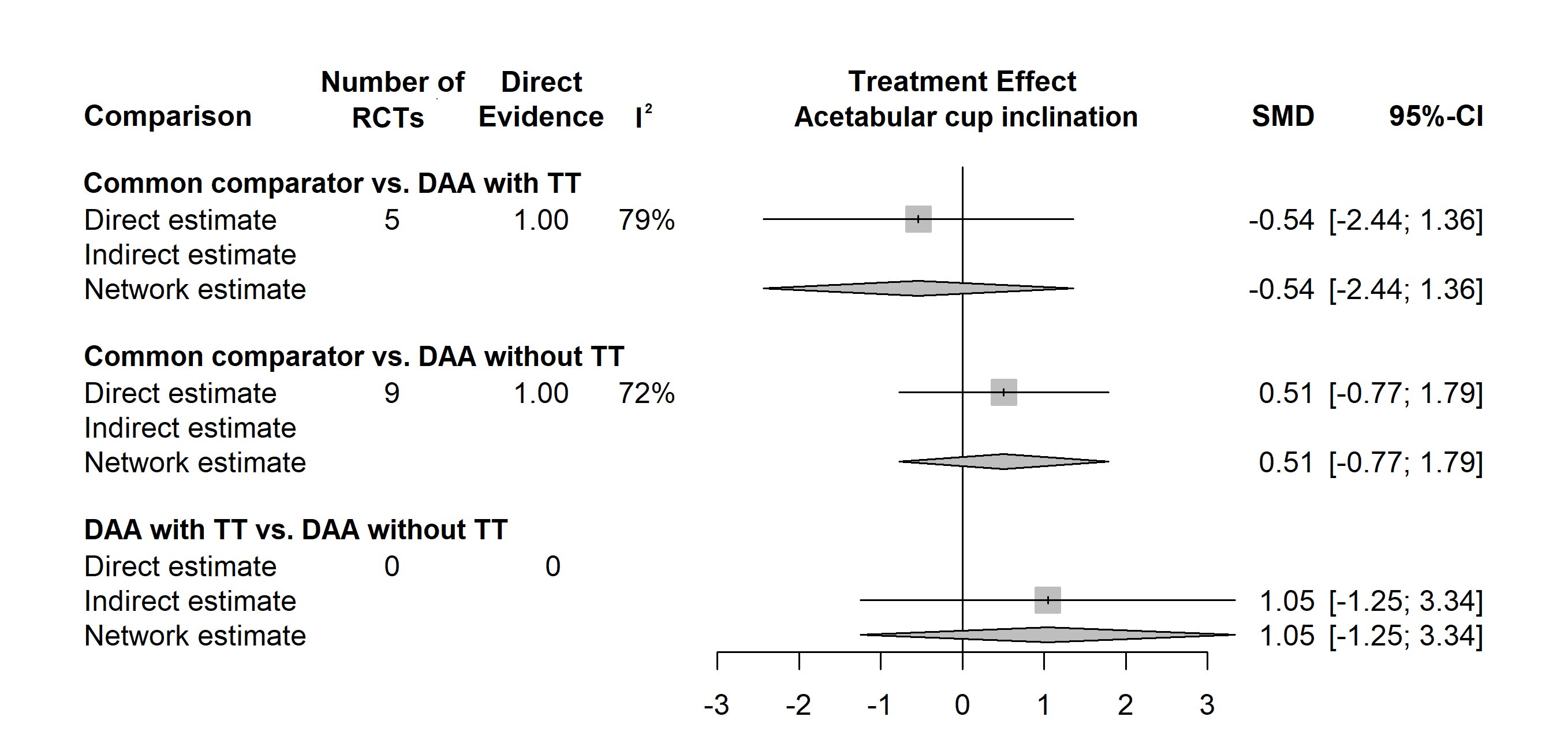

Supplement: Supplementary file 1 [file 13018_2024_4852_MOESM1_ESM.zip › Supplementary/Supplemental Figure 3 - Forest plot Acetabular cup inclination.jpg]

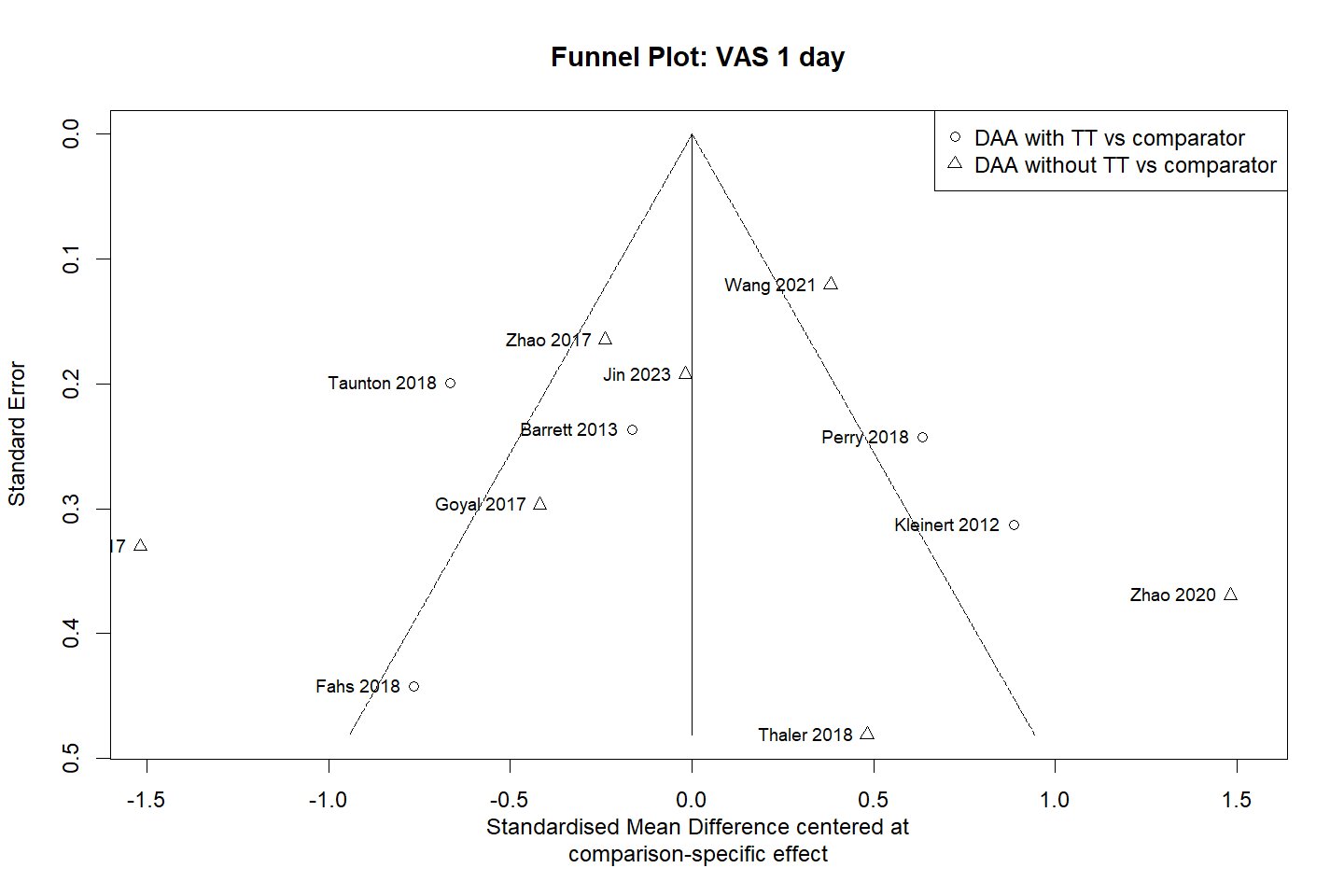

Supplement: Supplementary file 1 [file 13018_2024_4852_MOESM1_ESM.zip › Supplementary/Supplemental Figure 30 - Funnel plot VAS 1 day.jpg]

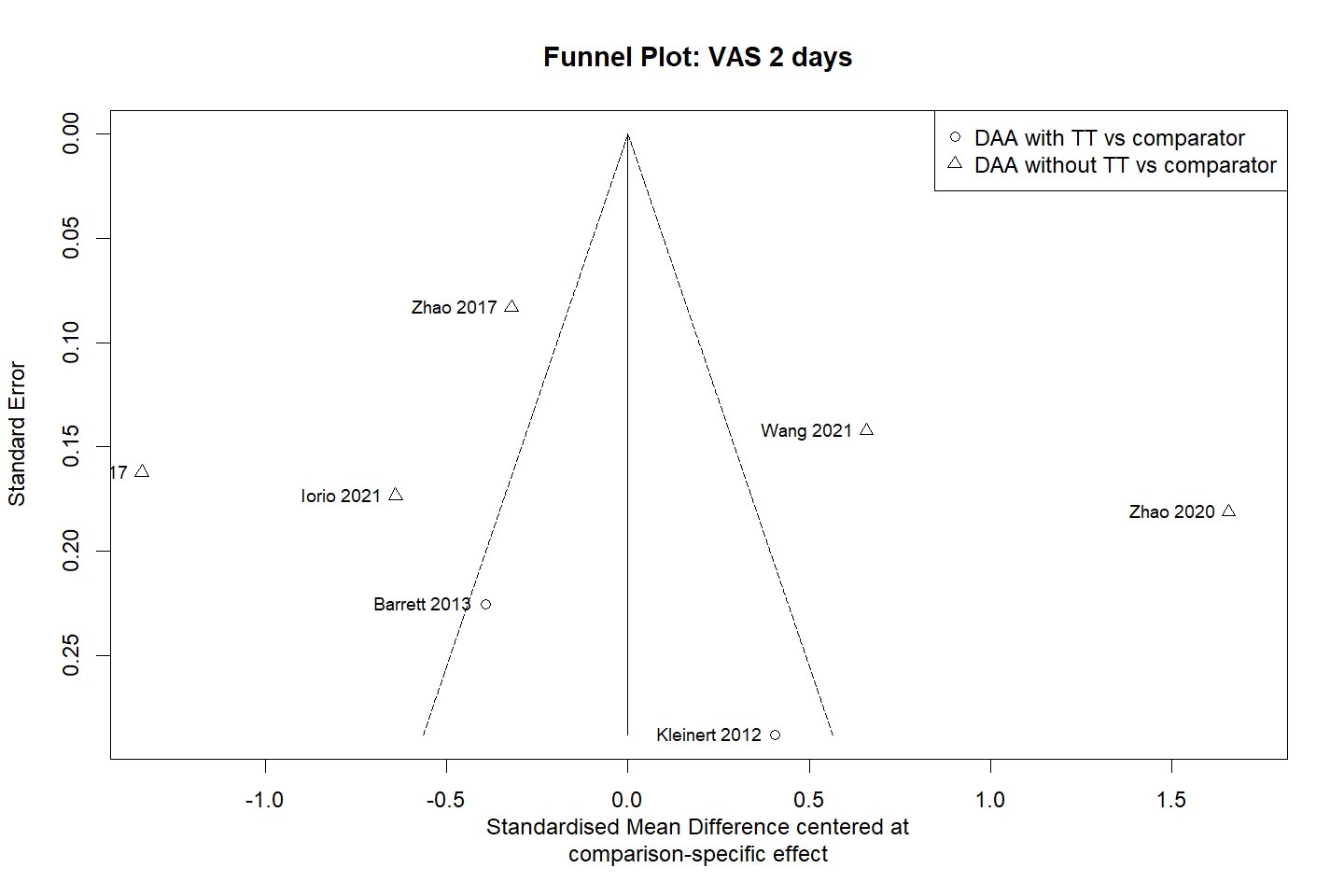

Supplement: Supplementary file 1 [file 13018_2024_4852_MOESM1_ESM.zip › Supplementary/Supplemental Figure 31 - Funnel plot VAS 2 days.jpg]

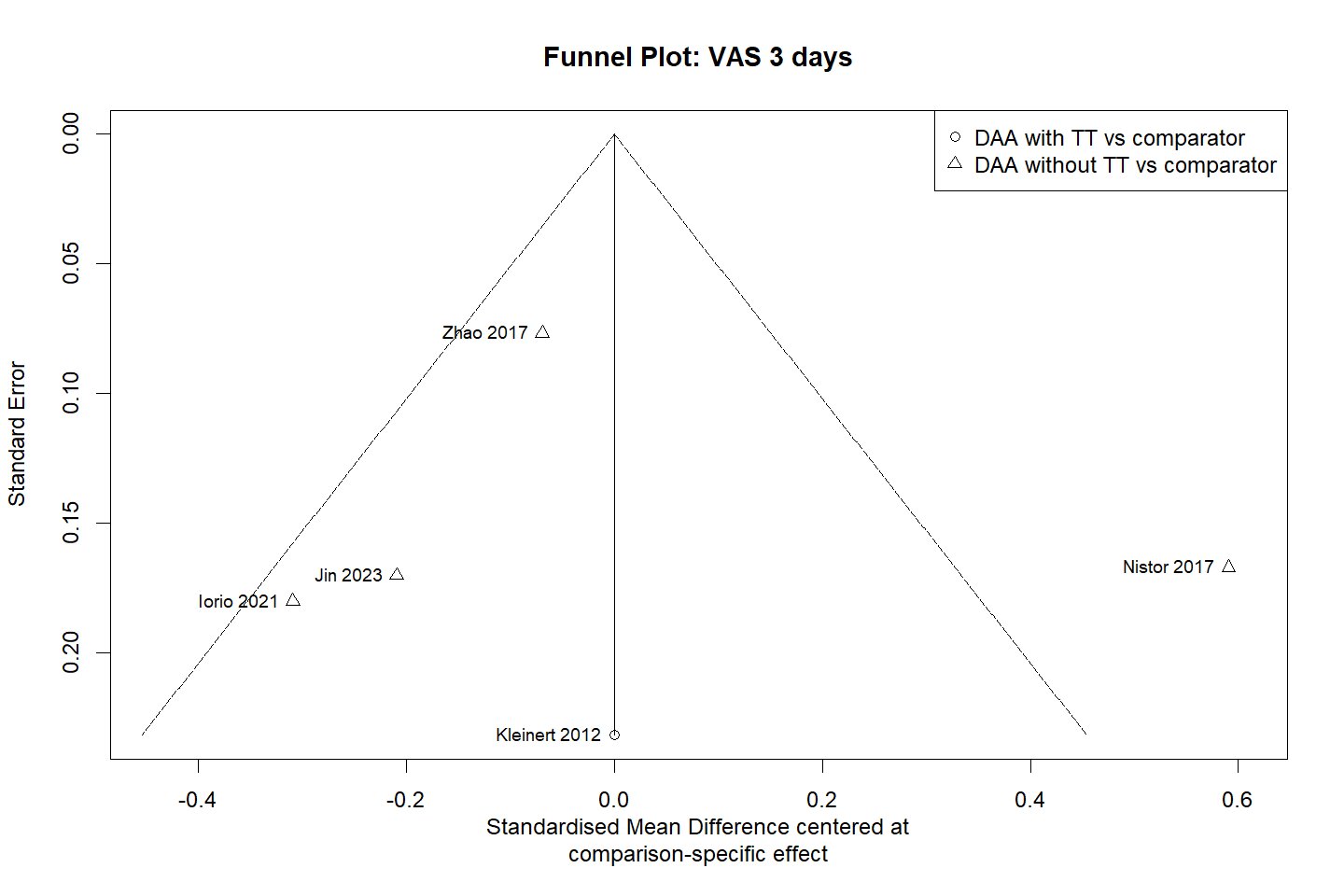

Supplement: Supplementary file 1 [file 13018_2024_4852_MOESM1_ESM.zip › Supplementary/Supplemental Figure 32 - Funnel plot VAS 3 days.jpg]

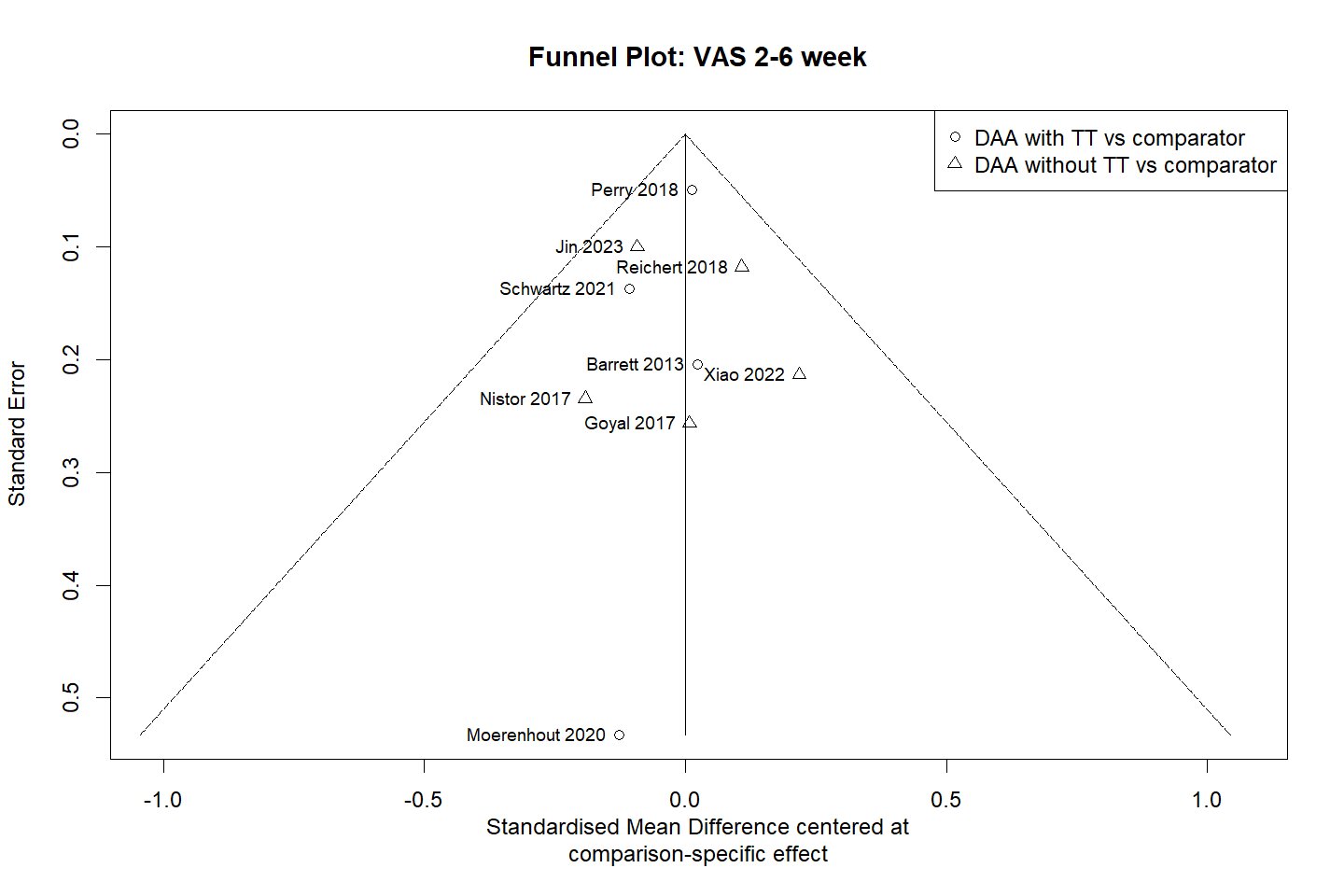

Supplement: Supplementary file 1 [file 13018_2024_4852_MOESM1_ESM.zip › Supplementary/Supplemental Figure 33 - Funnel plot VAS 2-6 week.jpg]

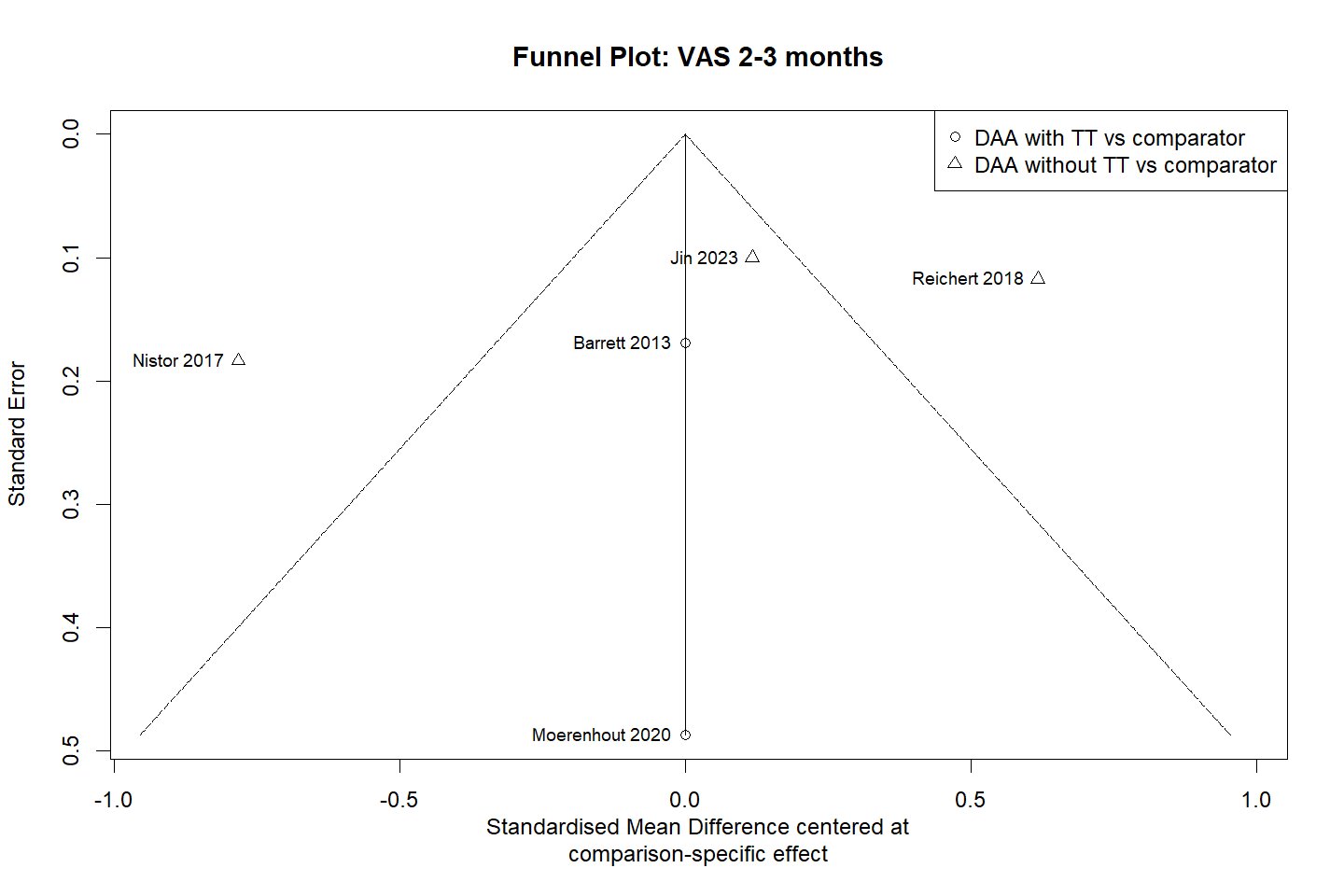

Supplement: Supplementary file 1 [file 13018_2024_4852_MOESM1_ESM.zip › Supplementary/Supplemental Figure 34 - Funnel plot VAS 2-3 months.jpg]

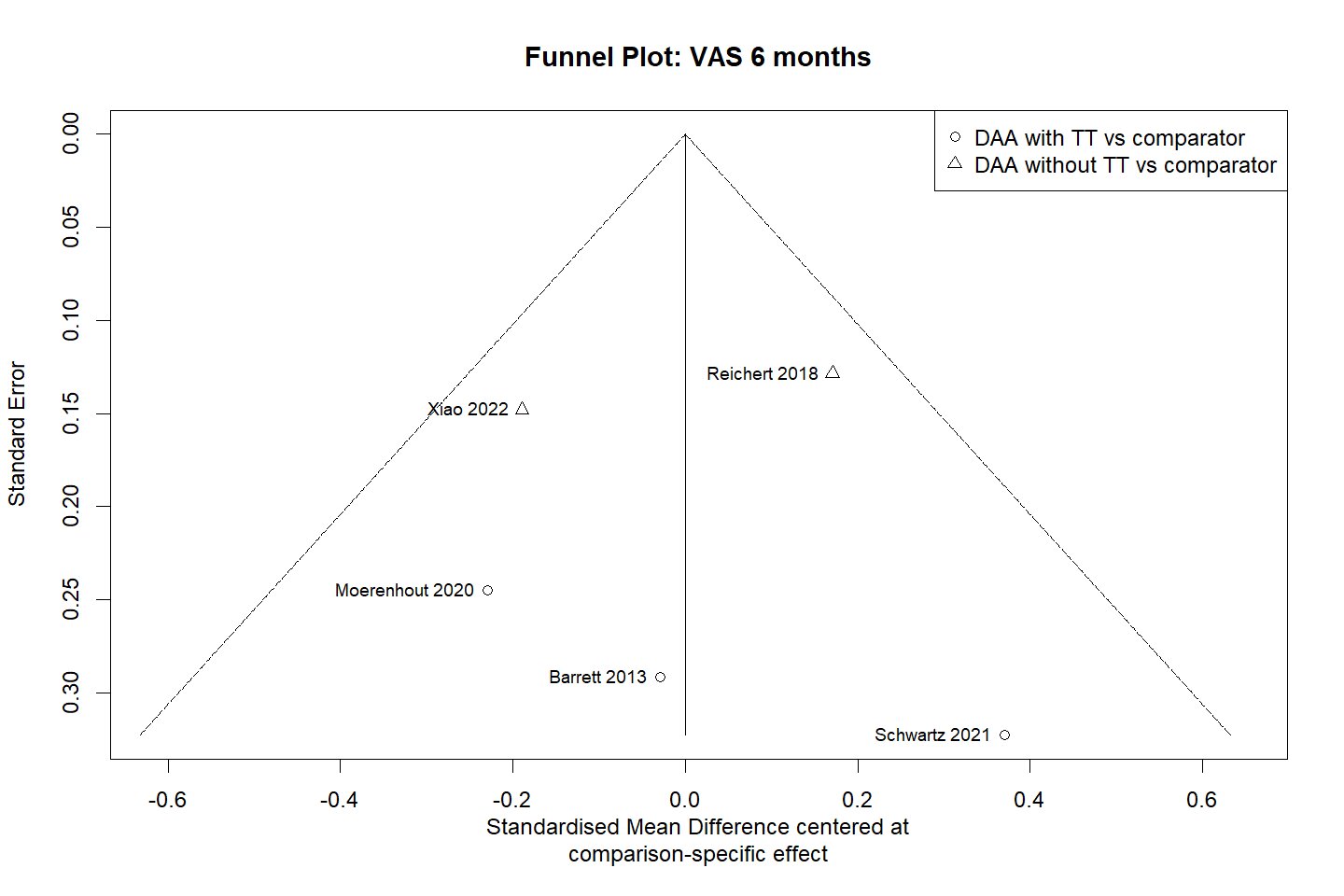

Supplement: Supplementary file 1 [file 13018_2024_4852_MOESM1_ESM.zip › Supplementary/Supplemental Figure 35 - Funnel plot VAS 6 months.jpg]

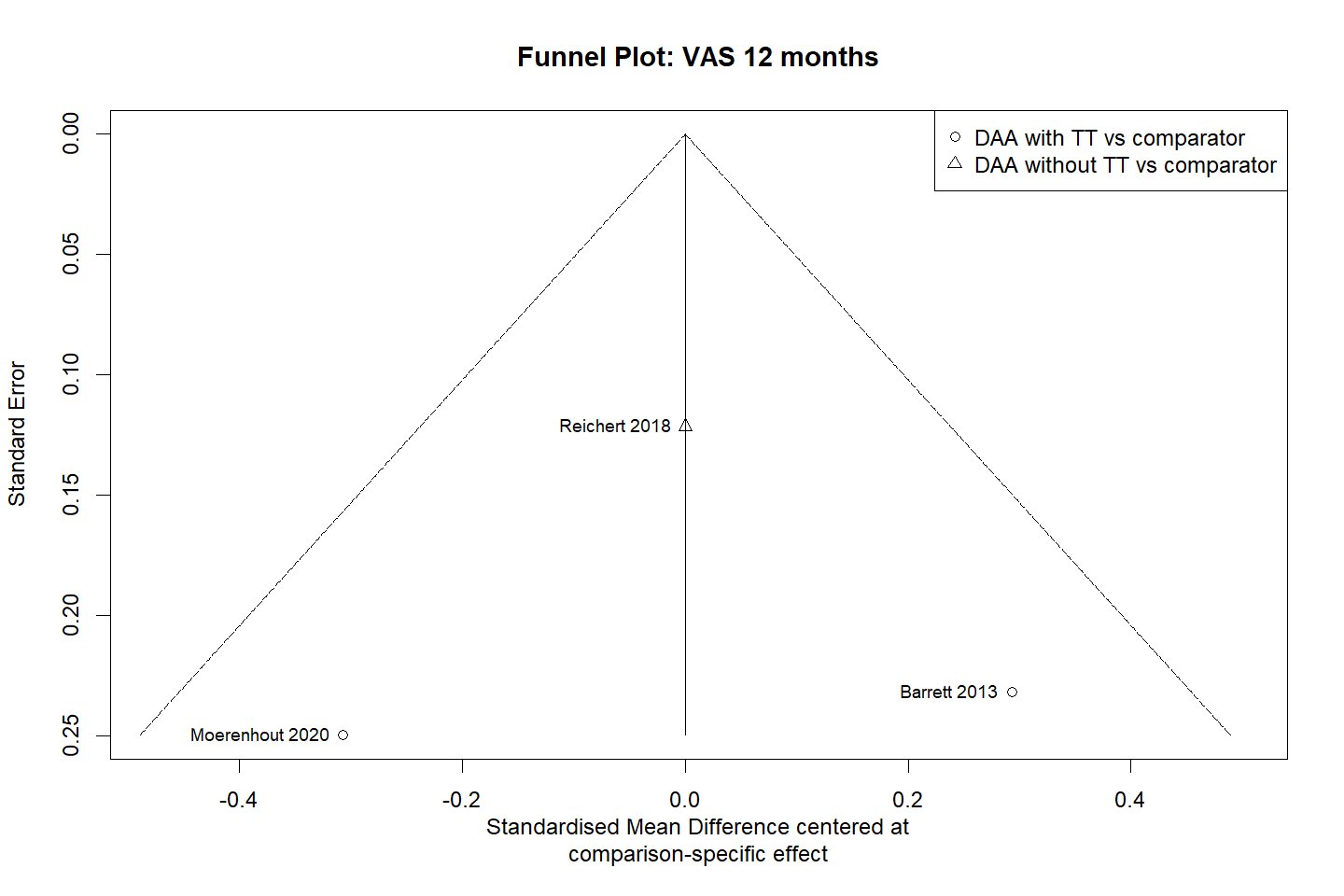

Supplement: Supplementary file 1 [file 13018_2024_4852_MOESM1_ESM.zip › Supplementary/Supplemental Figure 36 - Funnel plot VAS 12 months.jpg]

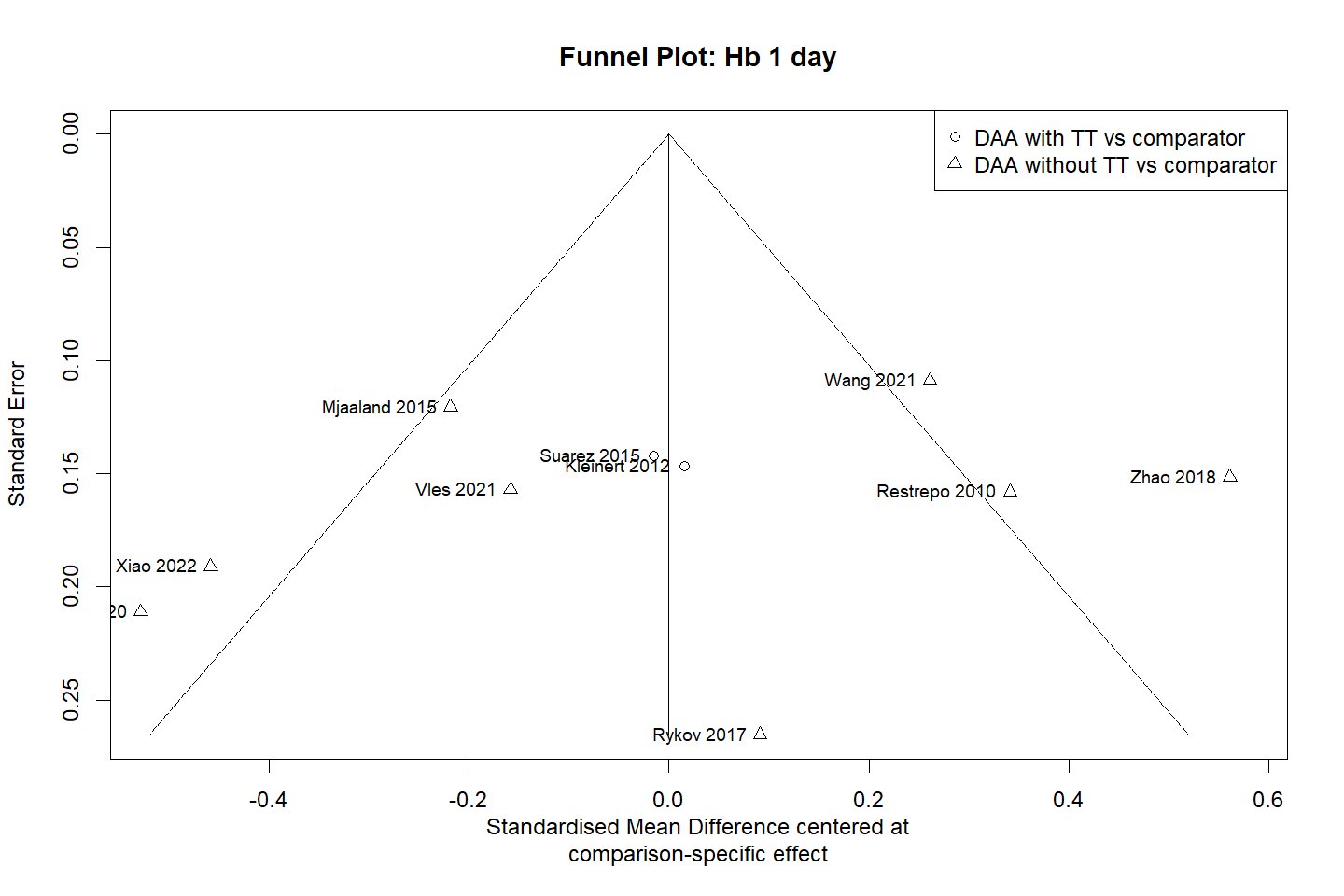

Supplement: Supplementary file 1 [file 13018_2024_4852_MOESM1_ESM.zip › Supplementary/Supplemental Figure 37 - Funnel plot Hb 1 day.jpg]

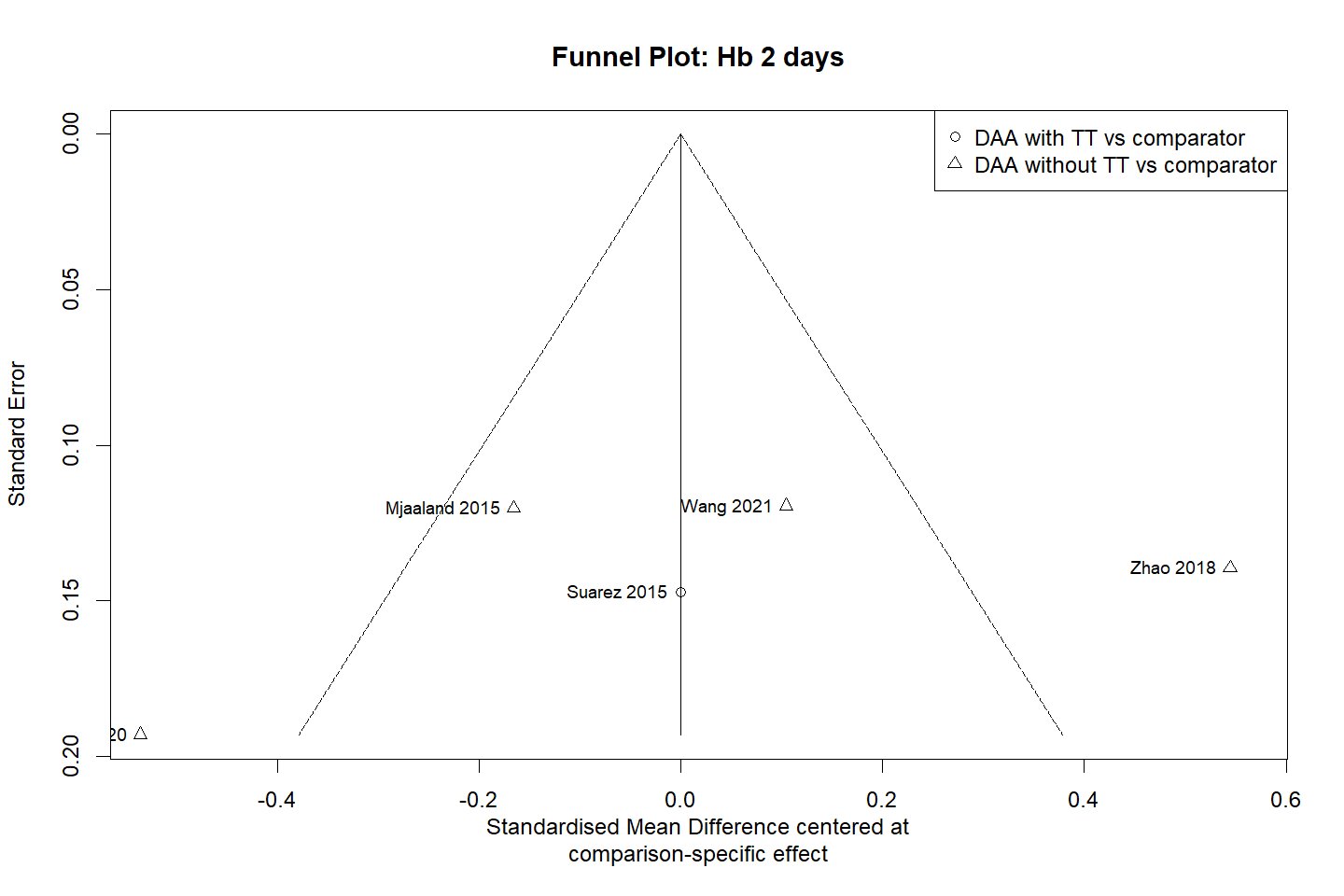

Supplement: Supplementary file 1 [file 13018_2024_4852_MOESM1_ESM.zip › Supplementary/Supplemental Figure 38 - Funnel plot Hb 2 days.jpg]

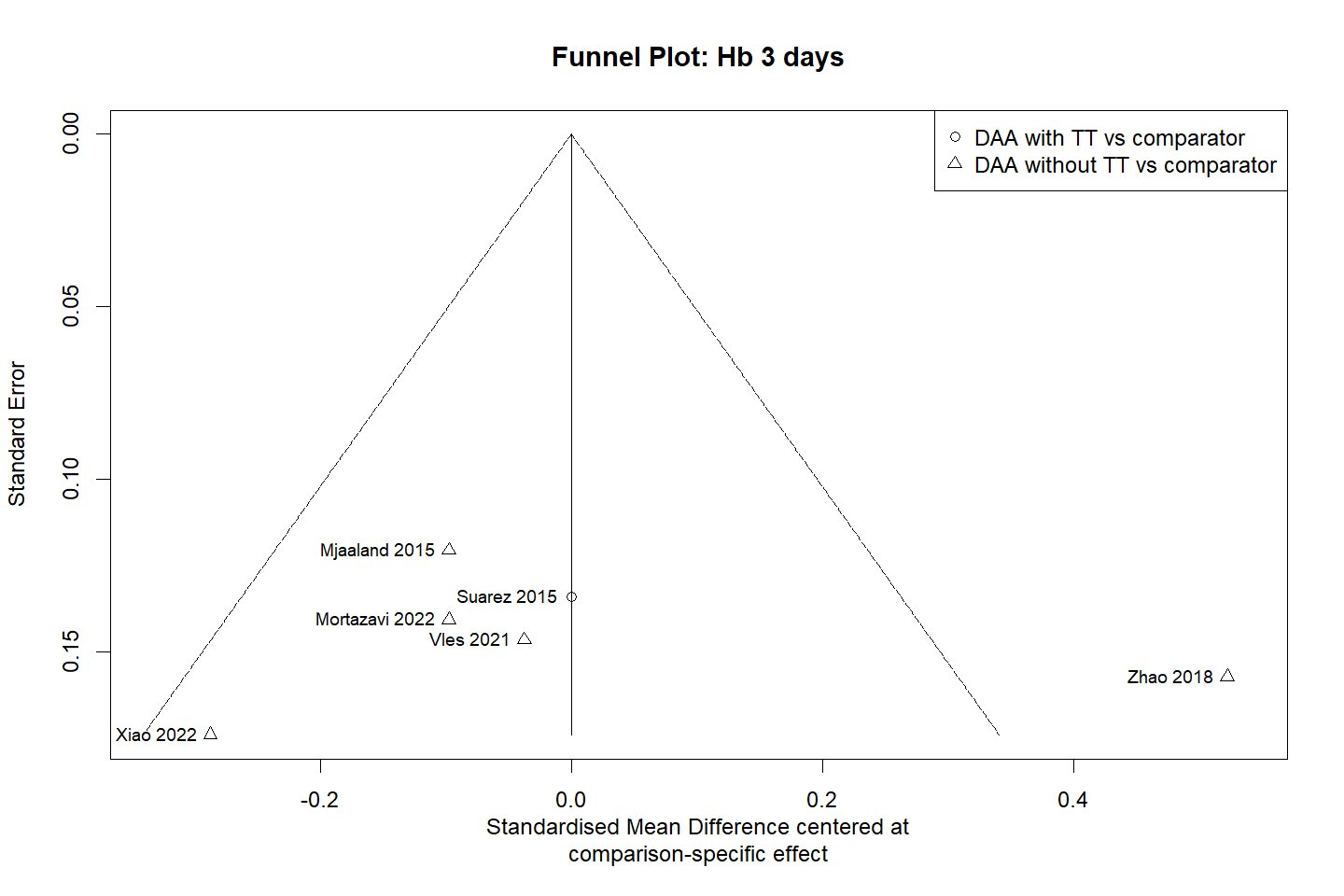

Supplement: Supplementary file 1 [file 13018_2024_4852_MOESM1_ESM.zip › Supplementary/Supplemental Figure 39 - Funnel plot Hb 3 days.jpg]

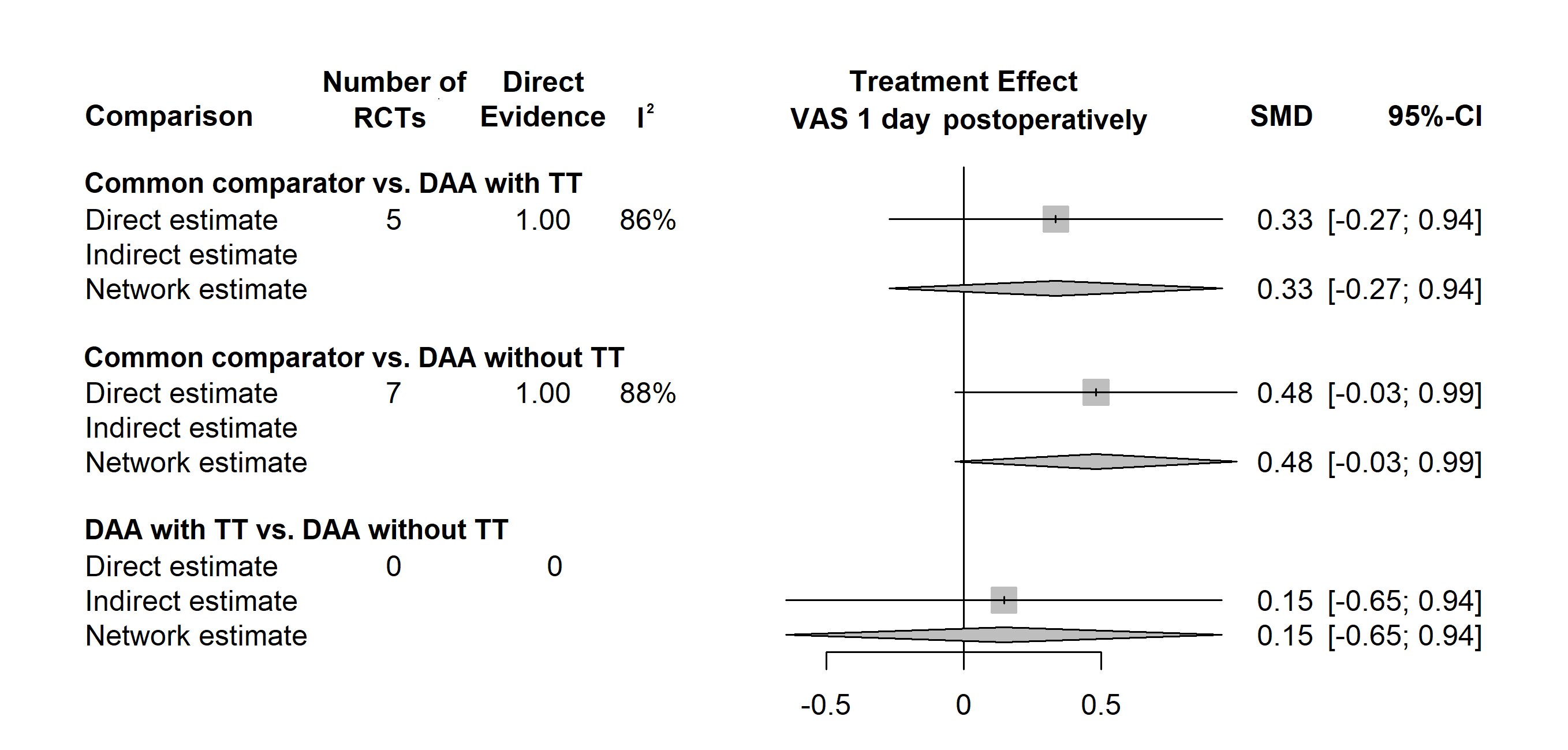

Supplement: Supplementary file 1 [file 13018_2024_4852_MOESM1_ESM.zip › Supplementary/Supplemental Figure 4 - Forest plot VAS 1 day.jpg]

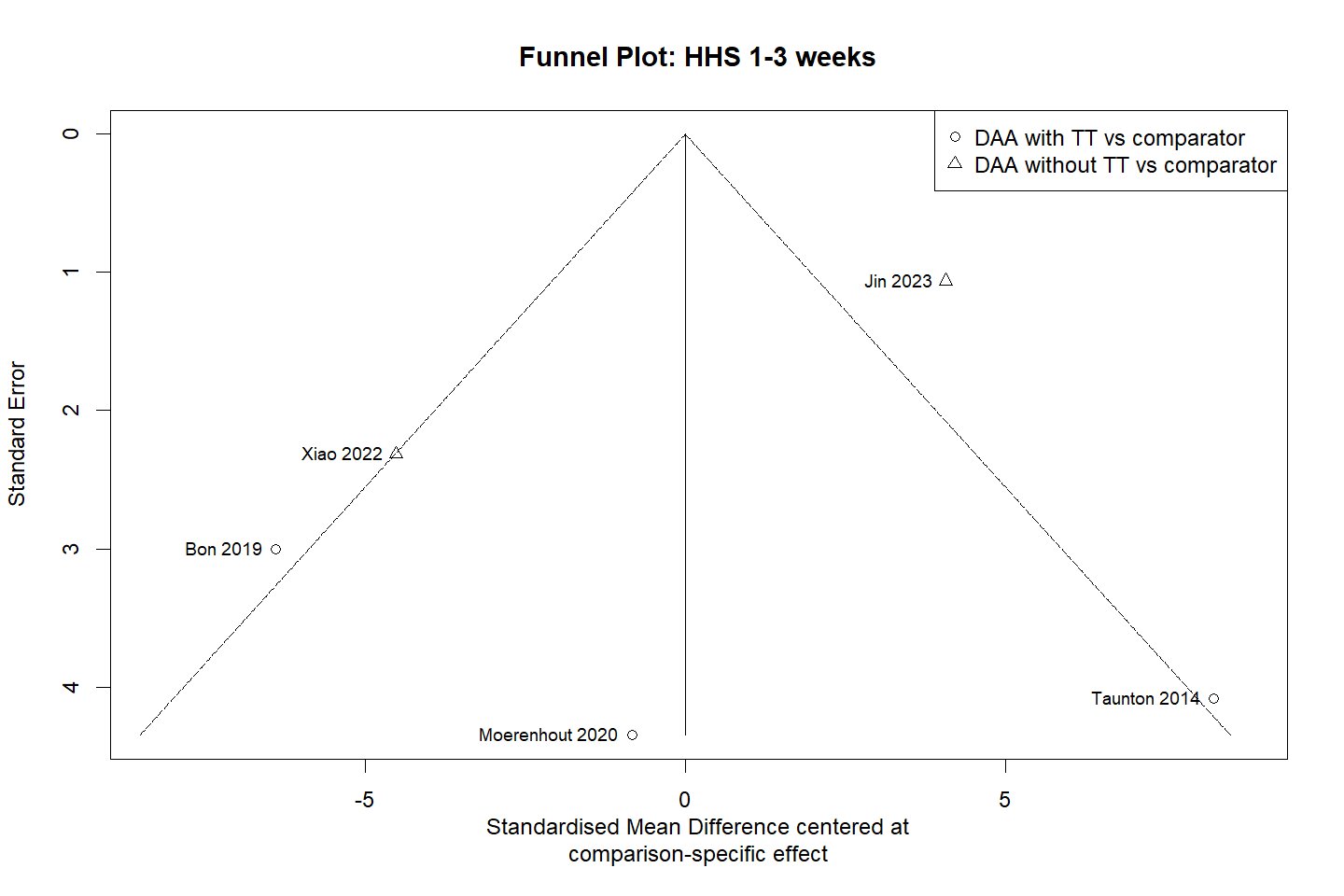

Supplement: Supplementary file 1 [file 13018_2024_4852_MOESM1_ESM.zip › Supplementary/Supplemental Figure 40 - Funnel plot HHS 1-3 weeks.jpg]

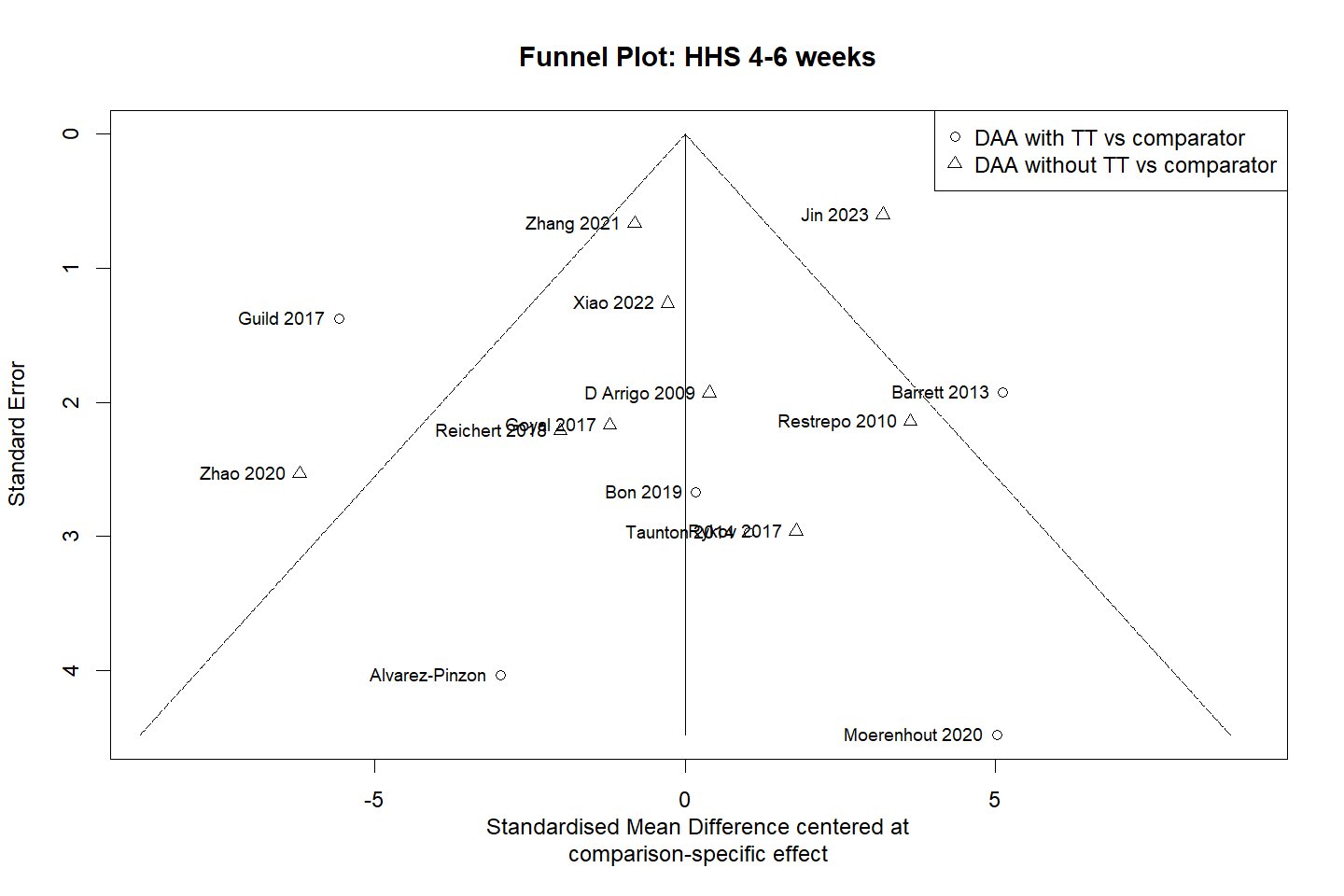

Supplement: Supplementary file 1 [file 13018_2024_4852_MOESM1_ESM.zip › Supplementary/Supplemental Figure 41 - Funnel plot HHS 4-6 weeks.jpg]

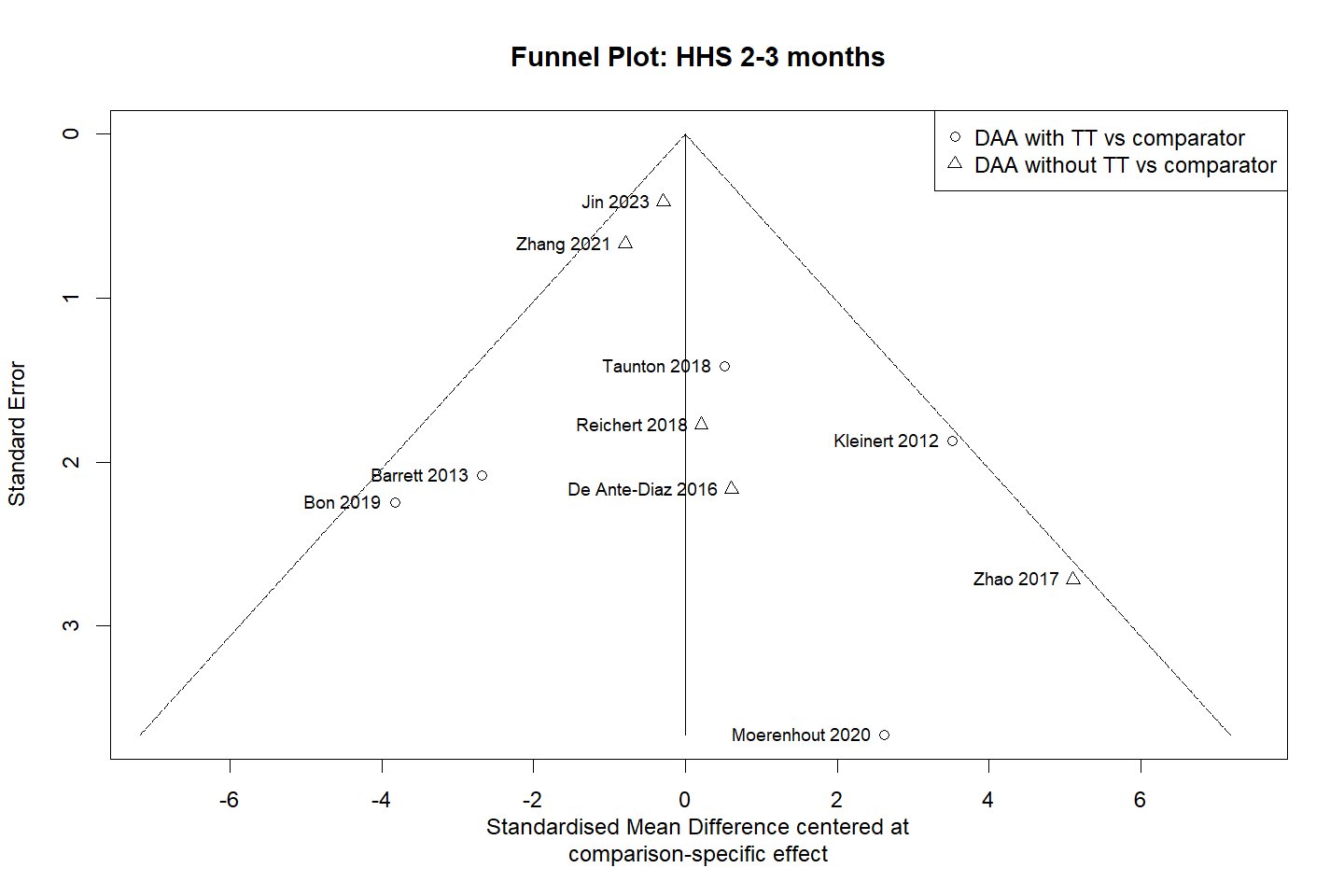

Supplement: Supplementary file 1 [file 13018_2024_4852_MOESM1_ESM.zip › Supplementary/Supplemental Figure 42 - Funnel plot HHS 2-3 months.jpg]

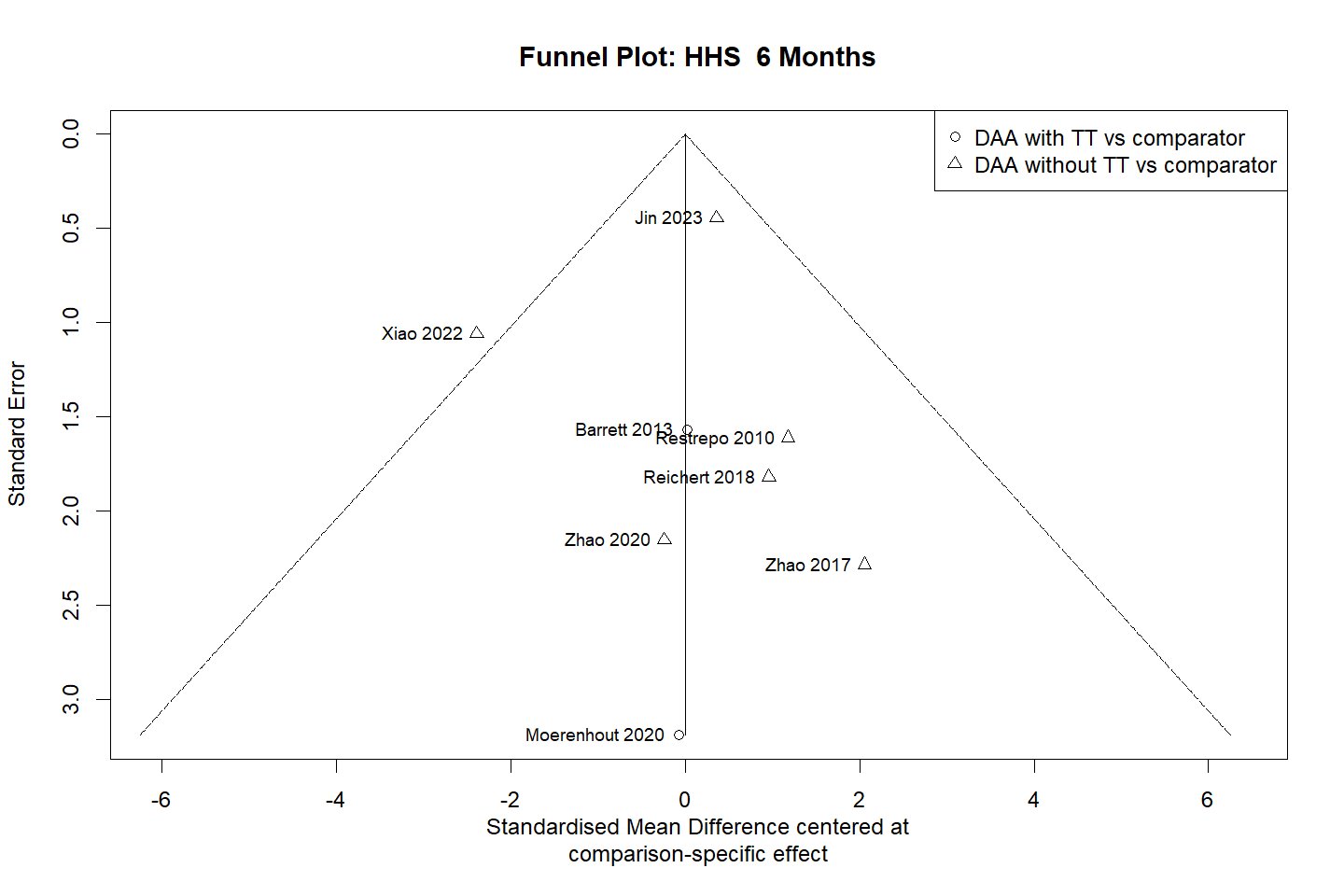

Supplement: Supplementary file 1 [file 13018_2024_4852_MOESM1_ESM.zip › Supplementary/Supplemental Figure 43 - Funnel plot HHS 6 months.jpg]

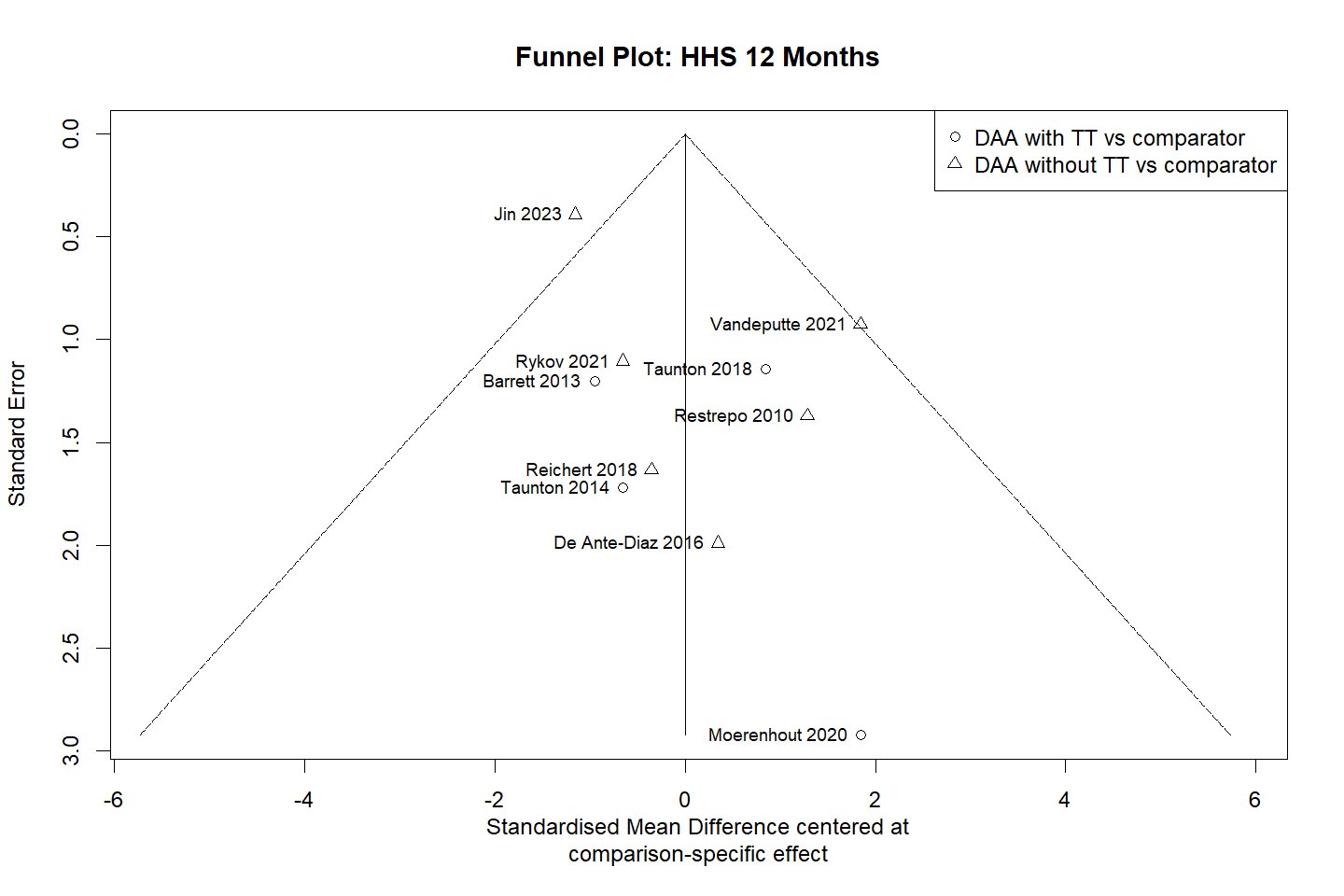

Supplement: Supplementary file 1 [file 13018_2024_4852_MOESM1_ESM.zip › Supplementary/Supplemental Figure 44 - Funnel plot HHS 12 months.jpg]

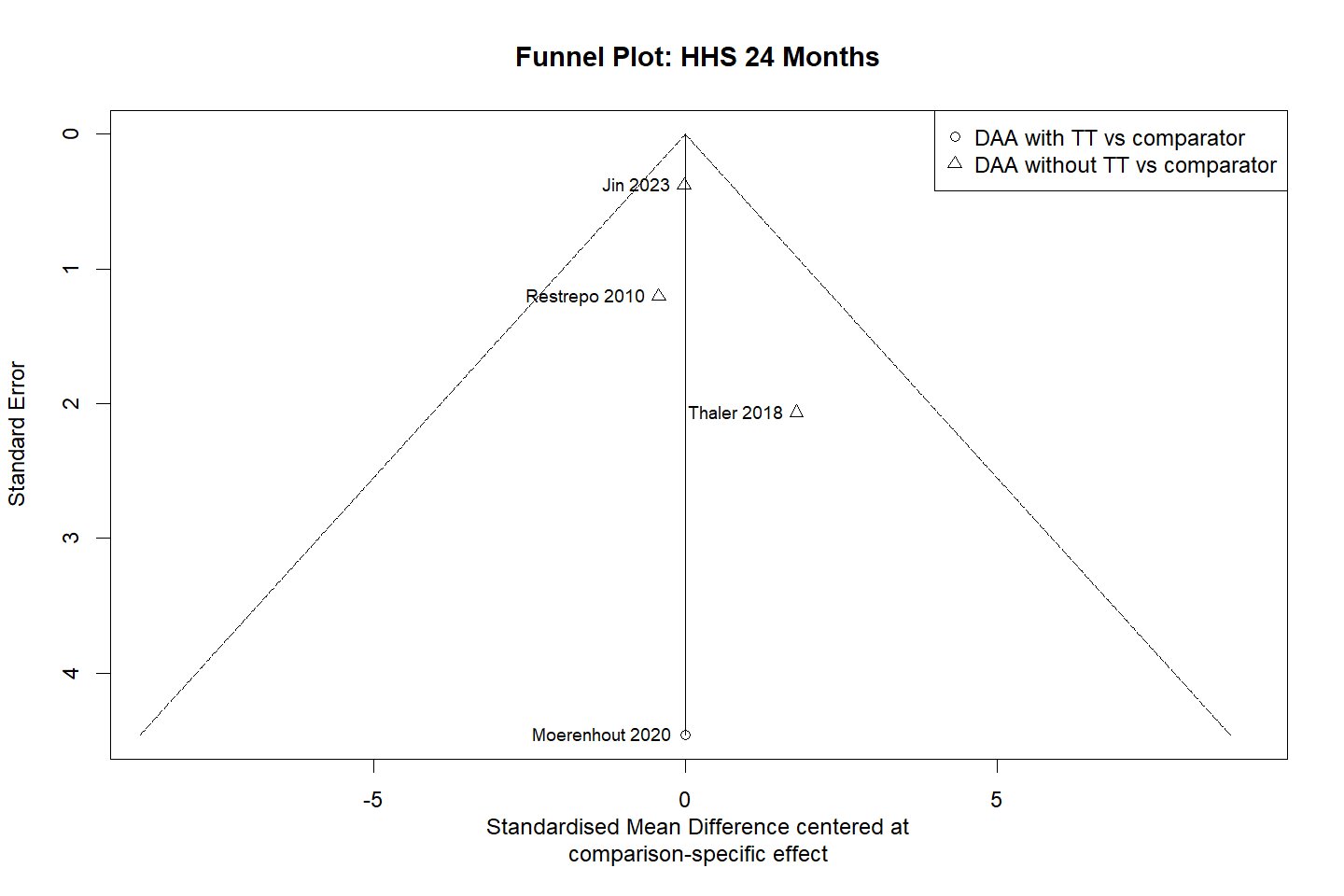

Supplement: Supplementary file 1 [file 13018_2024_4852_MOESM1_ESM.zip › Supplementary/Supplemental Figure 45 - Funnel plot HHS 24 months.jpg]

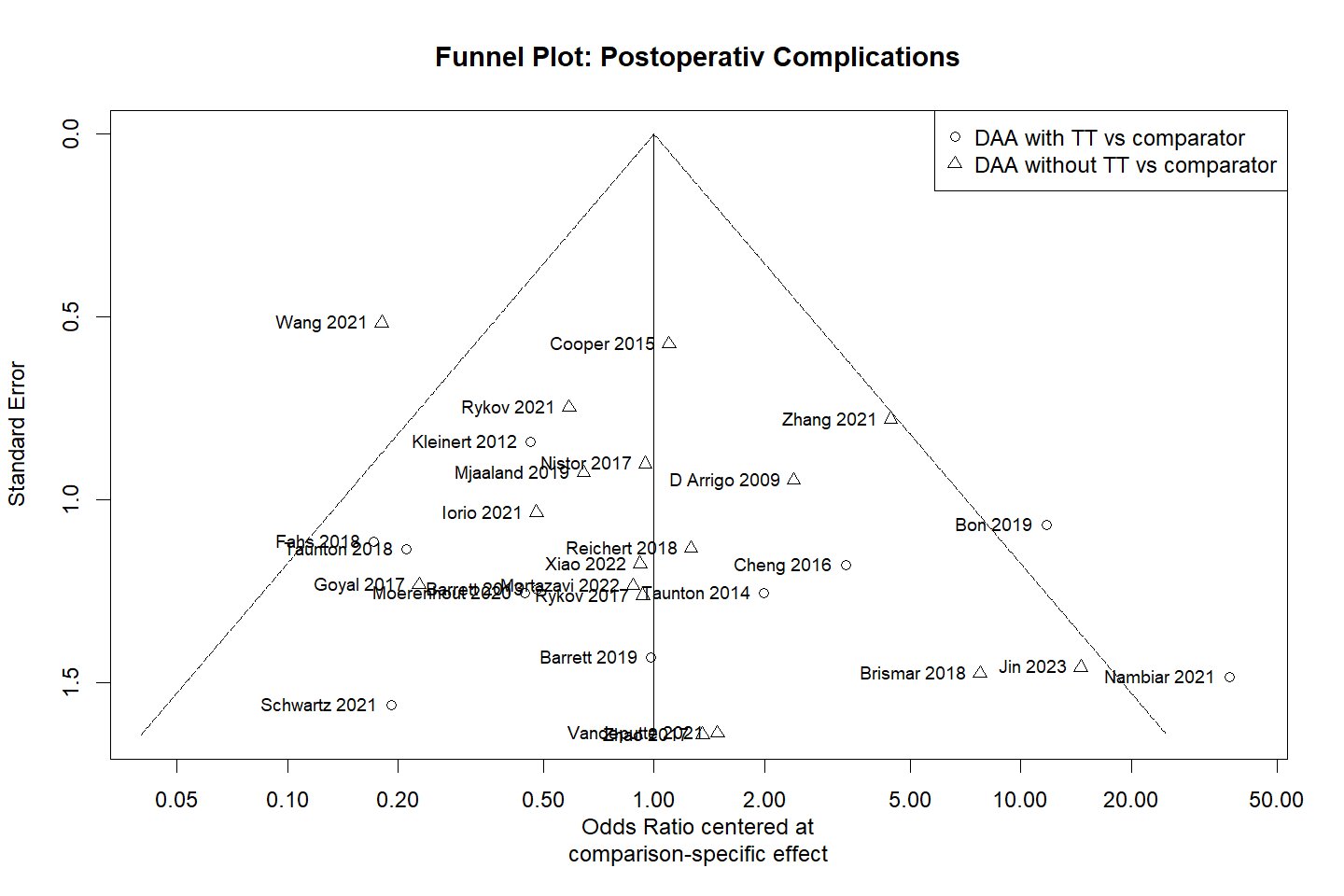

Supplement: Supplementary file 1 [file 13018_2024_4852_MOESM1_ESM.zip › Supplementary/Supplemental Figure 46 - Funnel plot Postoperative complications.jpg]

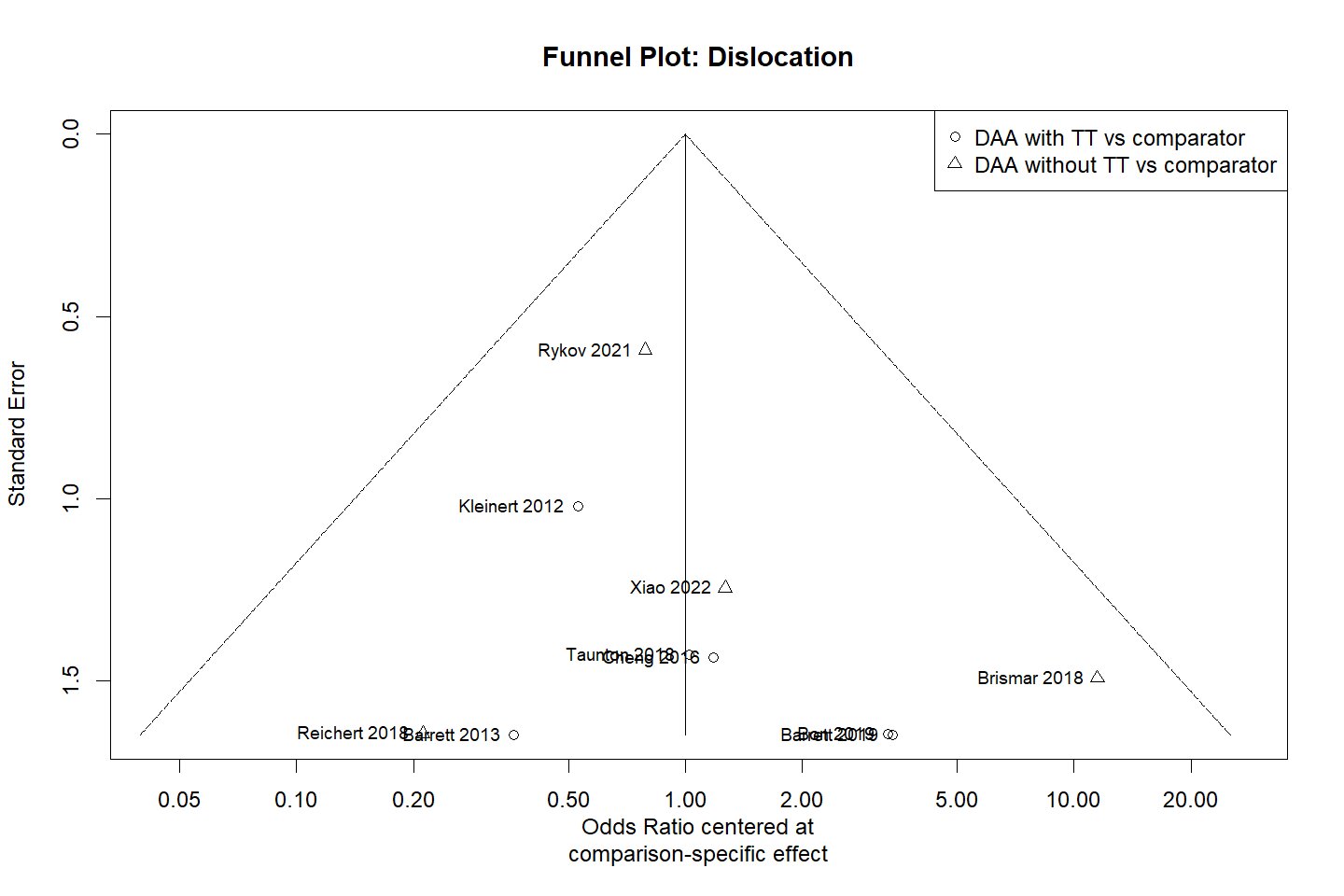

Supplement: Supplementary file 1 [file 13018_2024_4852_MOESM1_ESM.zip › Supplementary/Supplemental Figure 47 - Funnel plot Dislocation.jpg]

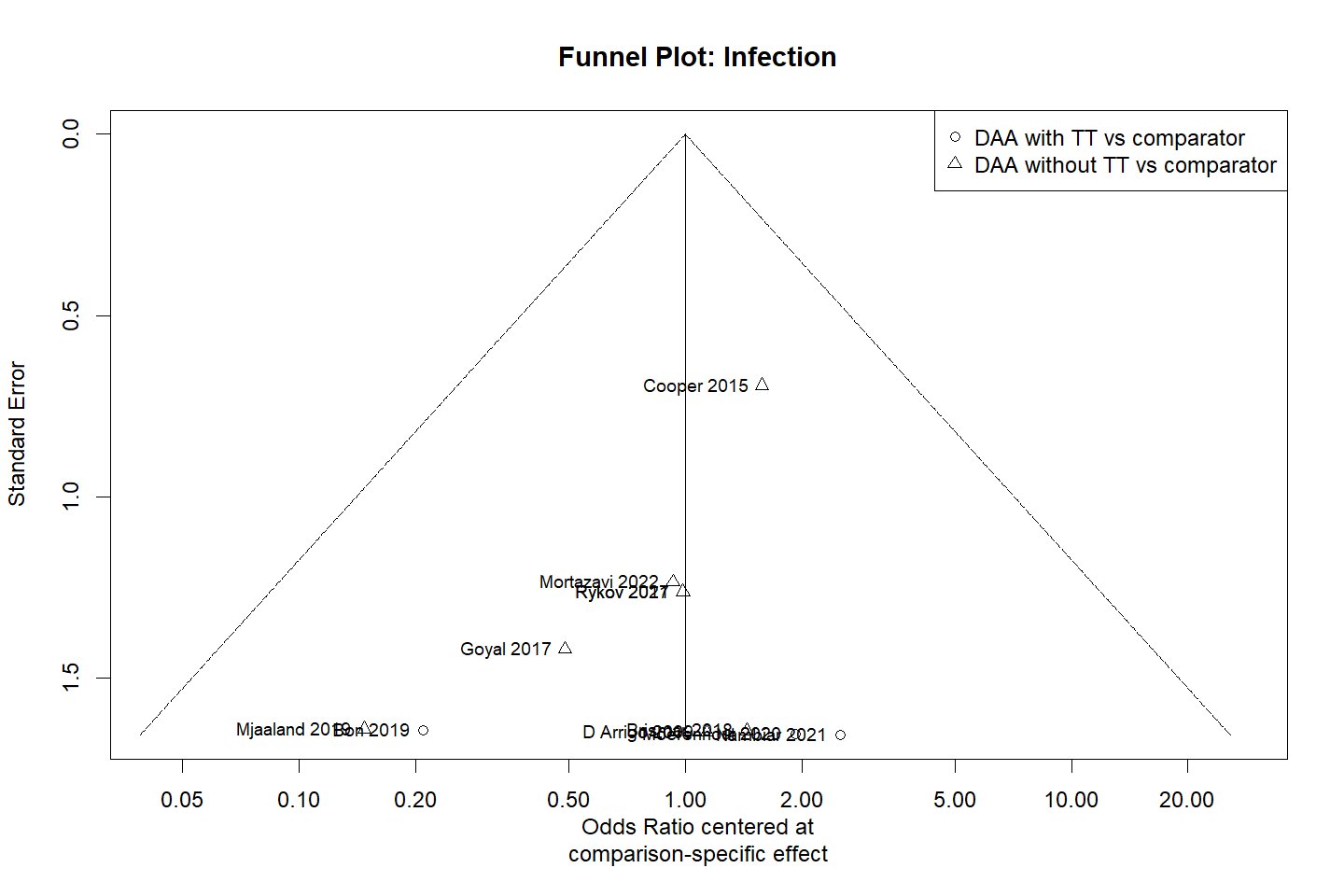

Supplement: Supplementary file 1 [file 13018_2024_4852_MOESM1_ESM.zip › Supplementary/Supplemental Figure 48 - Funnel plot Infection.jpg]

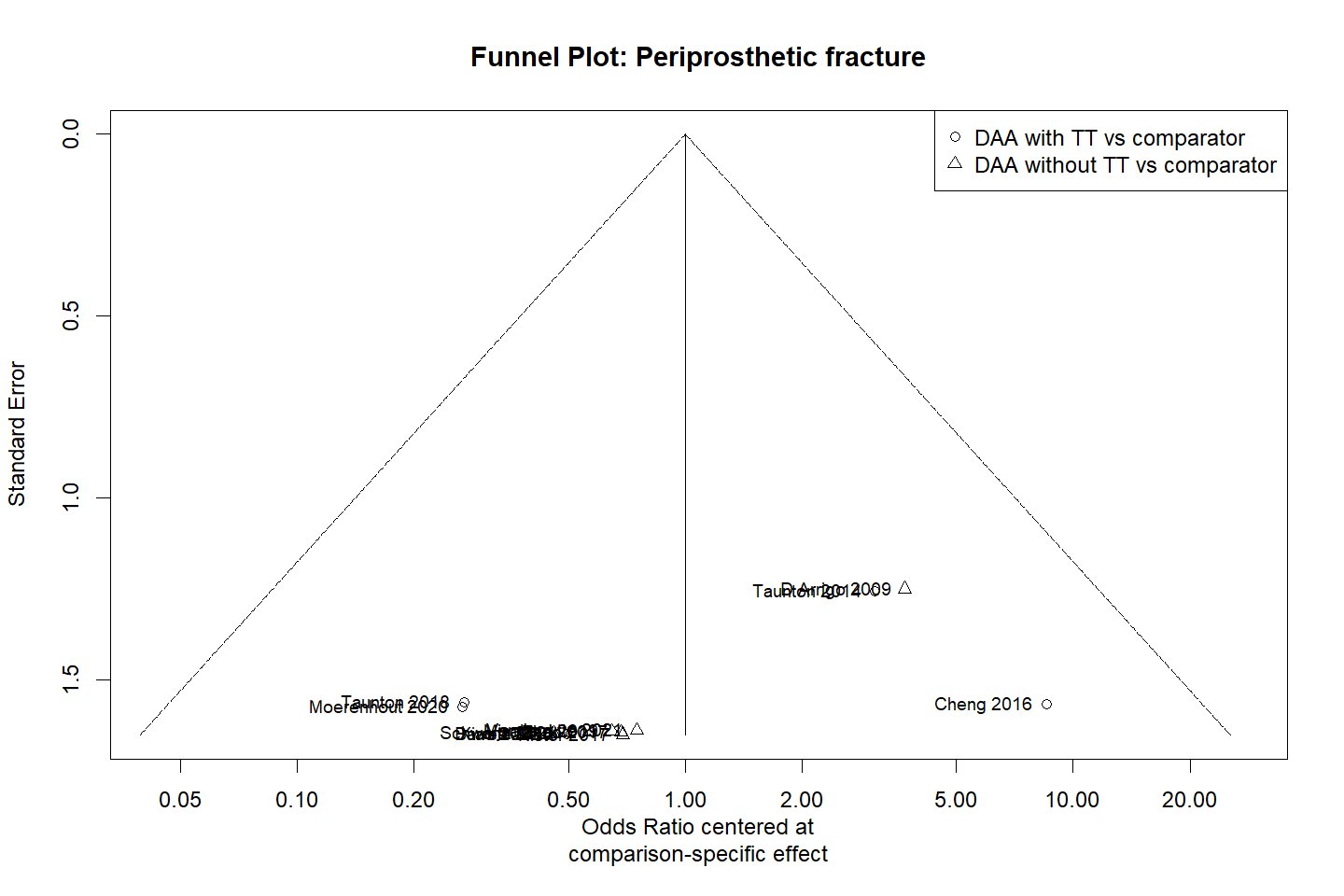

Supplement: Supplementary file 1 [file 13018_2024_4852_MOESM1_ESM.zip › Supplementary/Supplemental Figure 49 - Funnel plot Periprosthetic fracture.jpg]

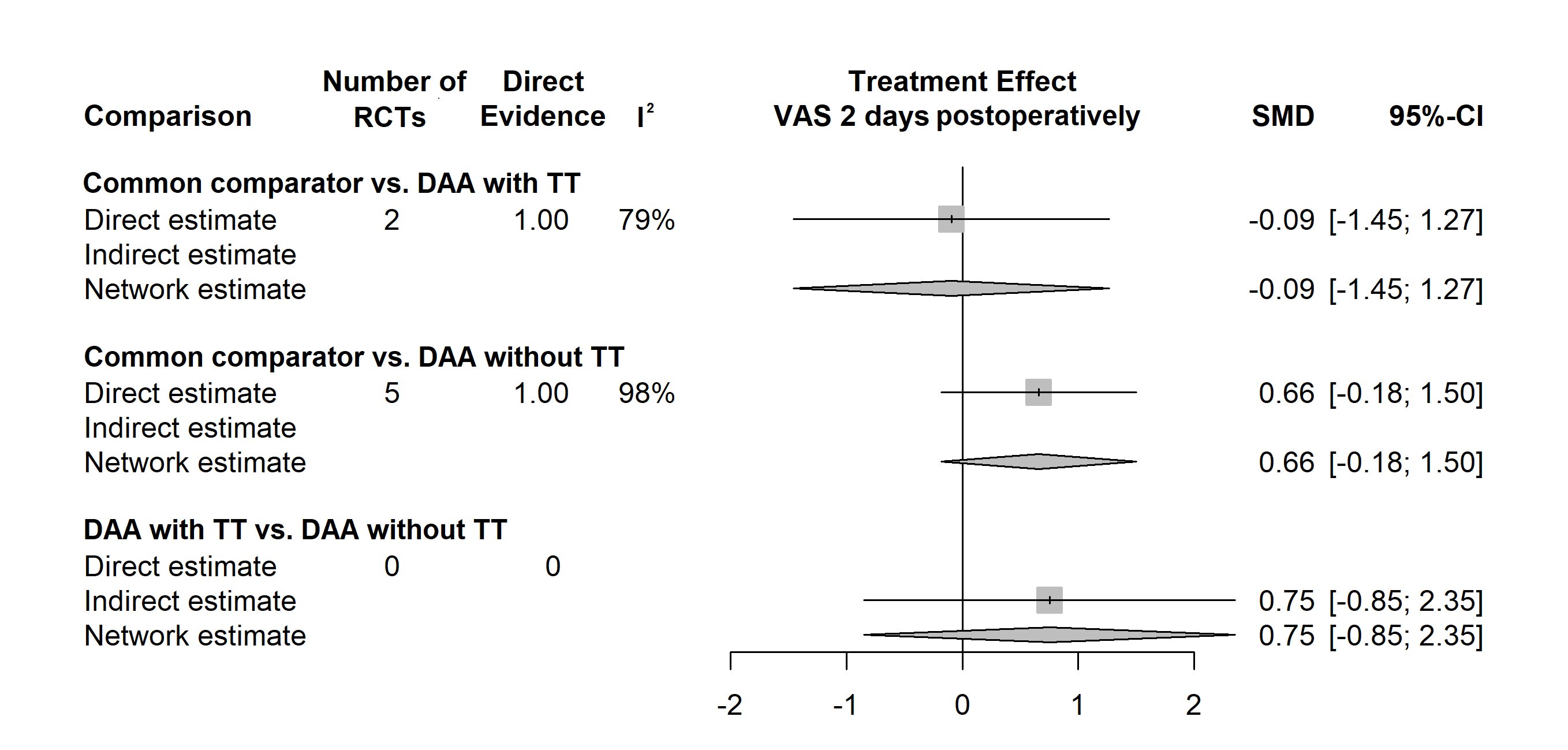

Supplement: Supplementary file 1 [file 13018_2024_4852_MOESM1_ESM.zip › Supplementary/Supplemental Figure 5 - Forest plot VAS 2 days.jpg]

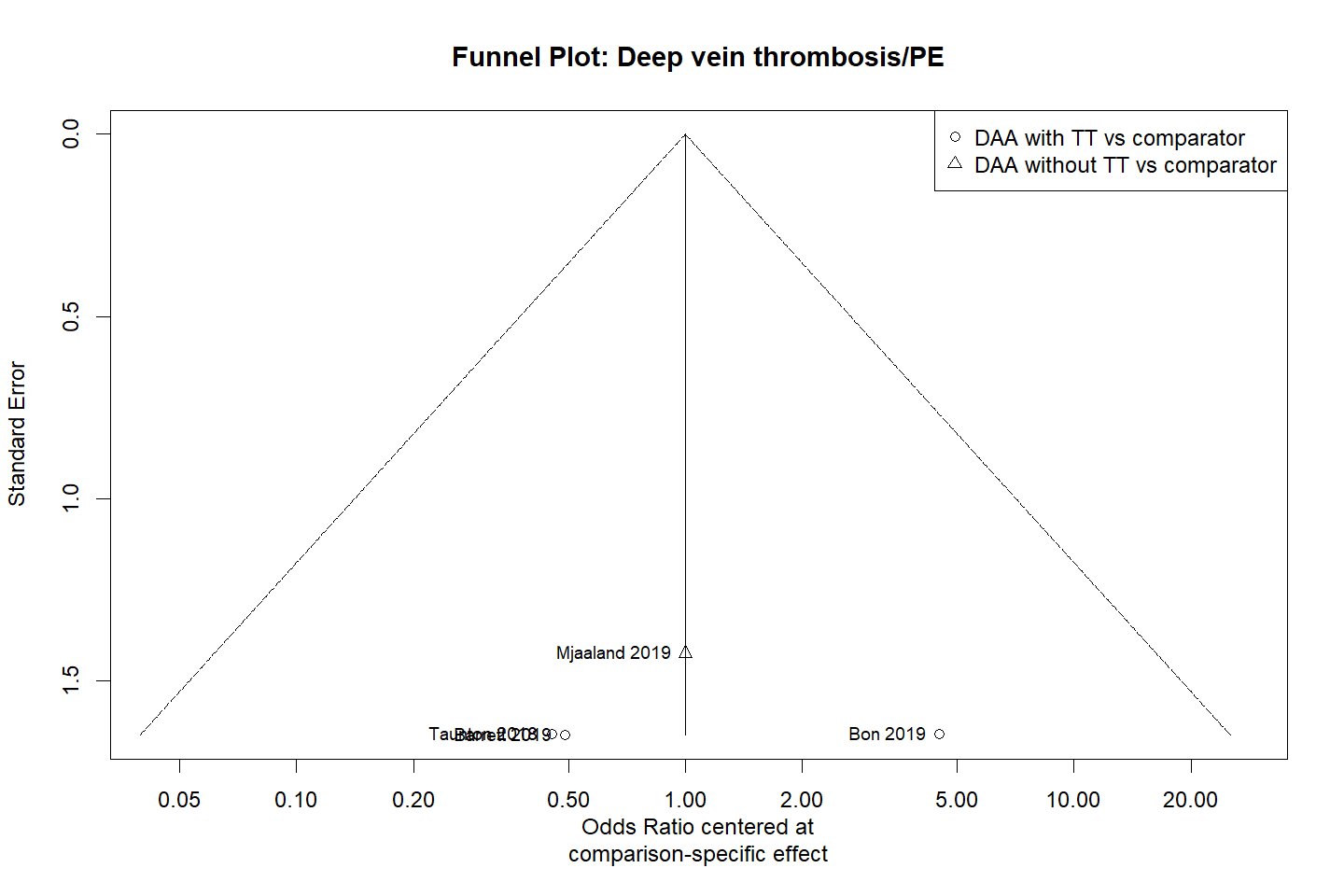

Supplement: Supplementary file 1 [file 13018_2024_4852_MOESM1_ESM.zip › Supplementary/Supplemental Figure 50 - Funnel plot DVT_PE.jpg]

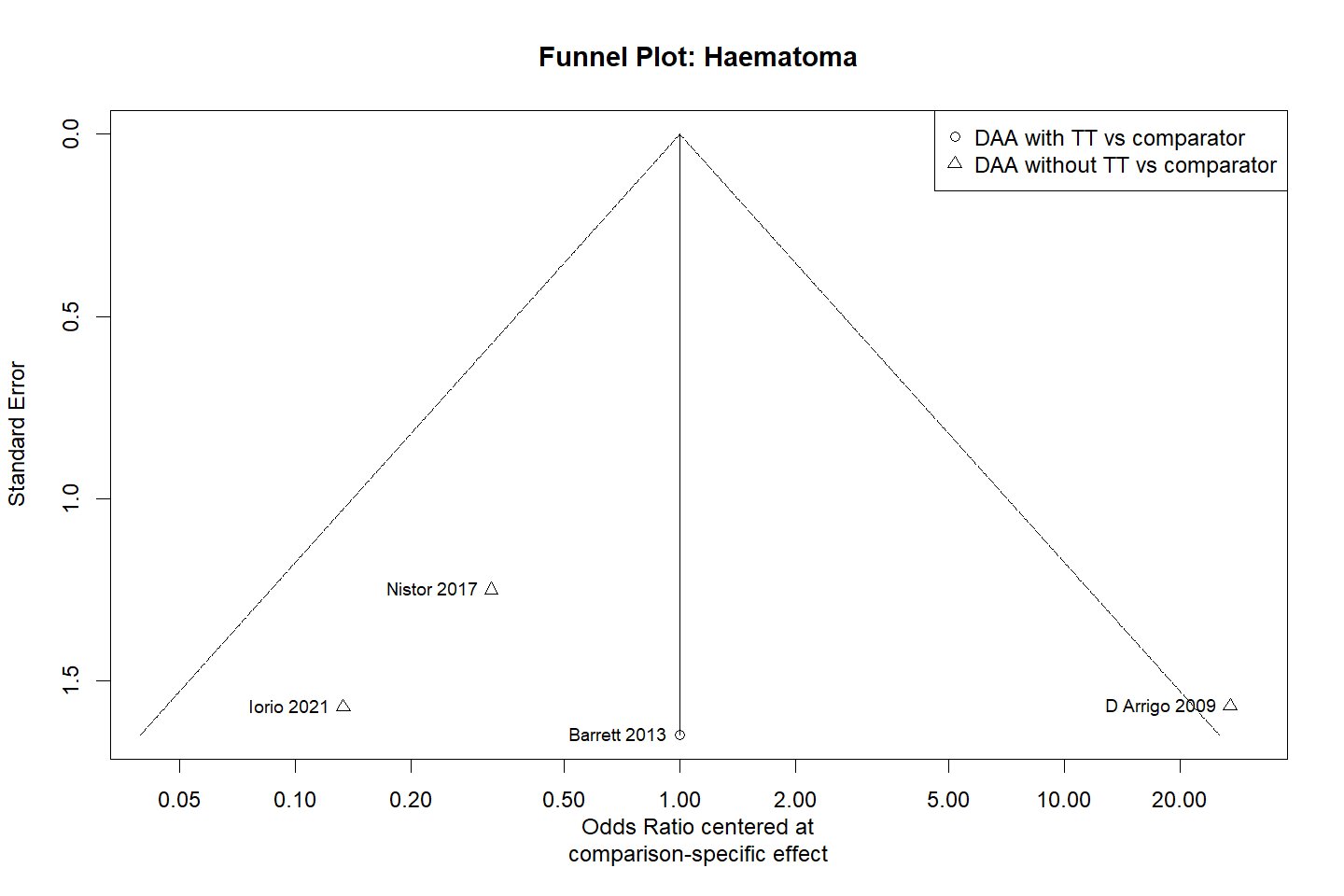

Supplement: Supplementary file 1 [file 13018_2024_4852_MOESM1_ESM.zip › Supplementary/Supplemental Figure 51 - Funnel plot Haematoma.jpg]

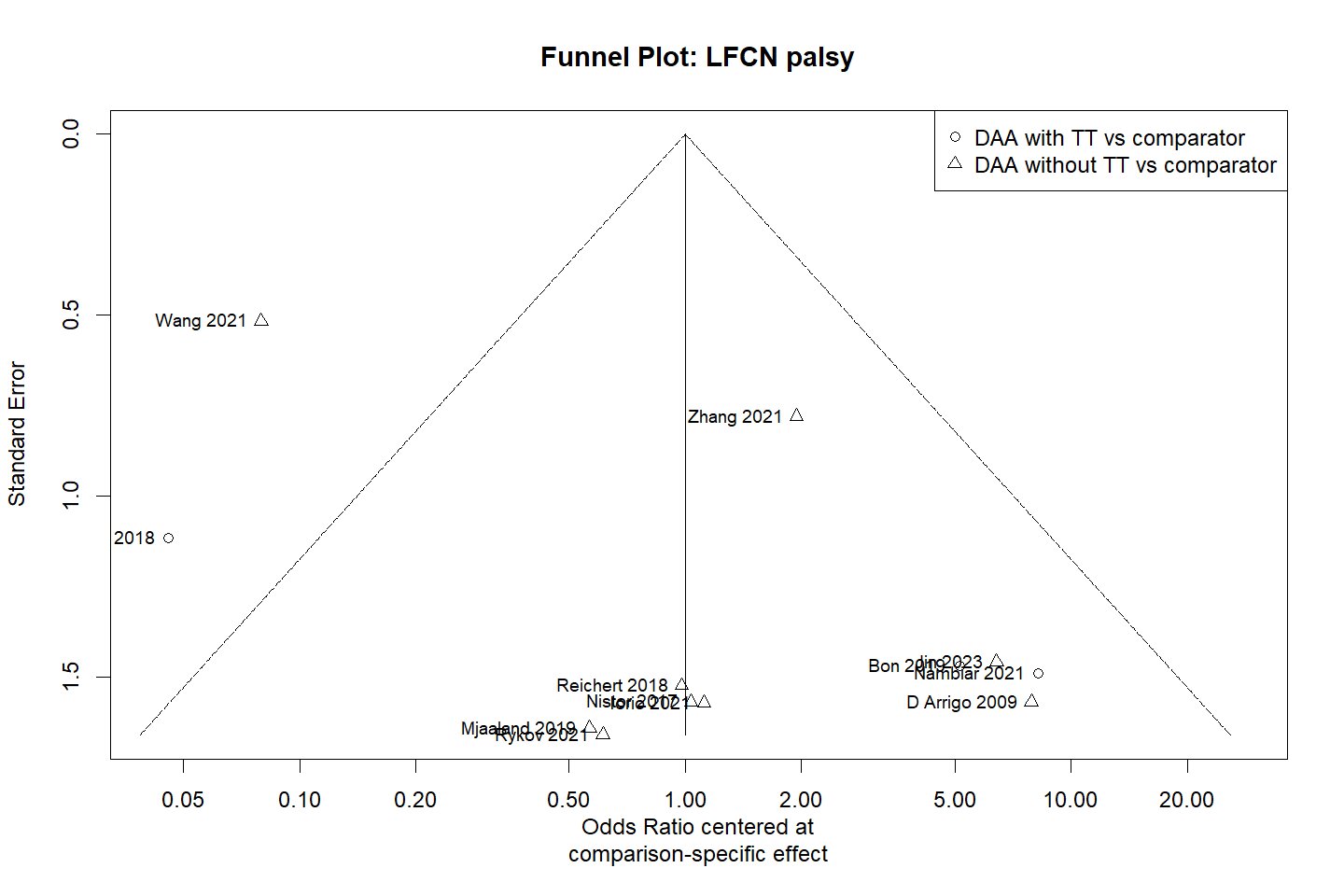

Supplement: Supplementary file 1 [file 13018_2024_4852_MOESM1_ESM.zip › Supplementary/Supplemental Figure 52 - Funnel plot LFCN palsy.jpg]

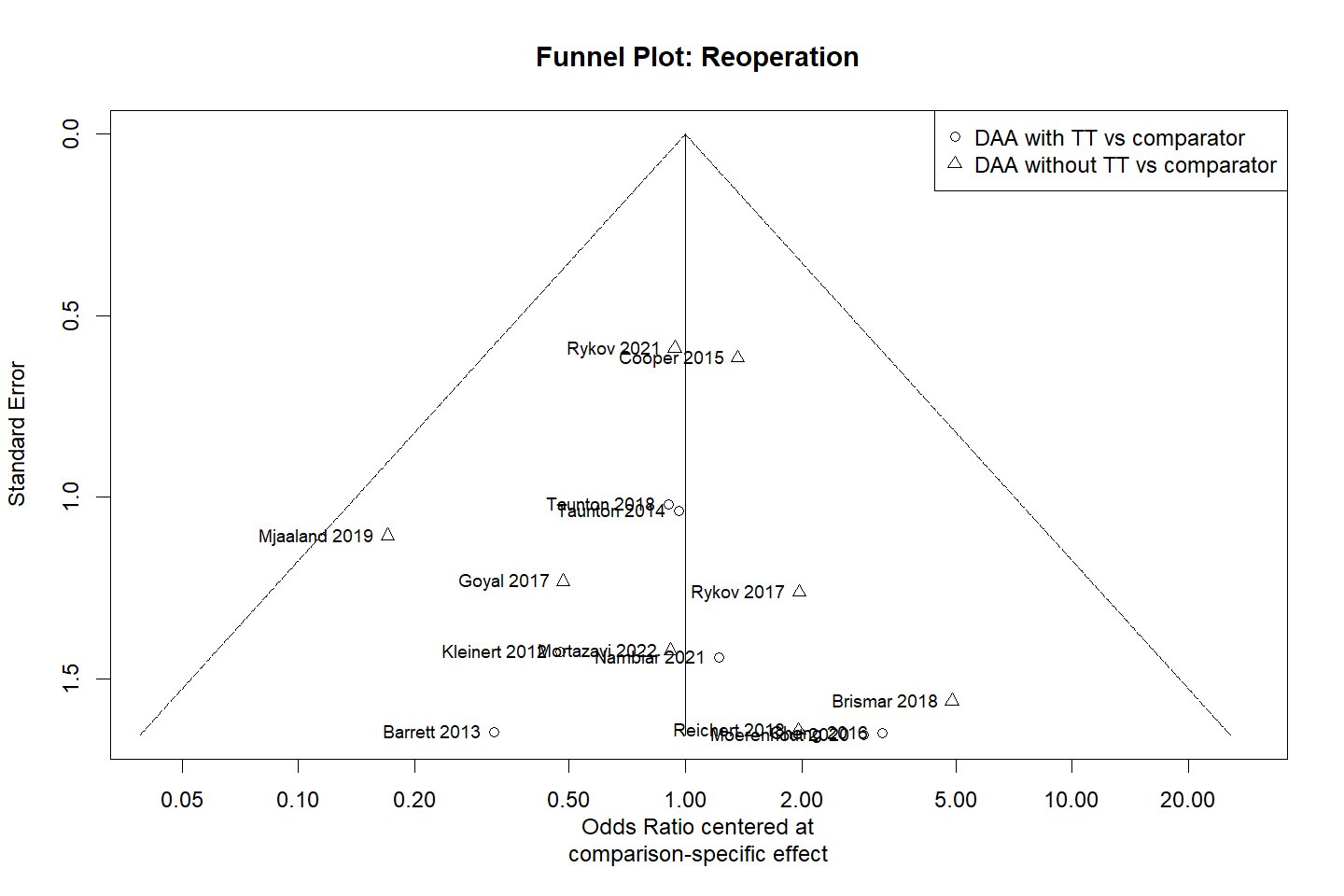

Supplement: Supplementary file 1 [file 13018_2024_4852_MOESM1_ESM.zip › Supplementary/Supplemental Figure 53 - Funnel plot Reoperation.jpg]

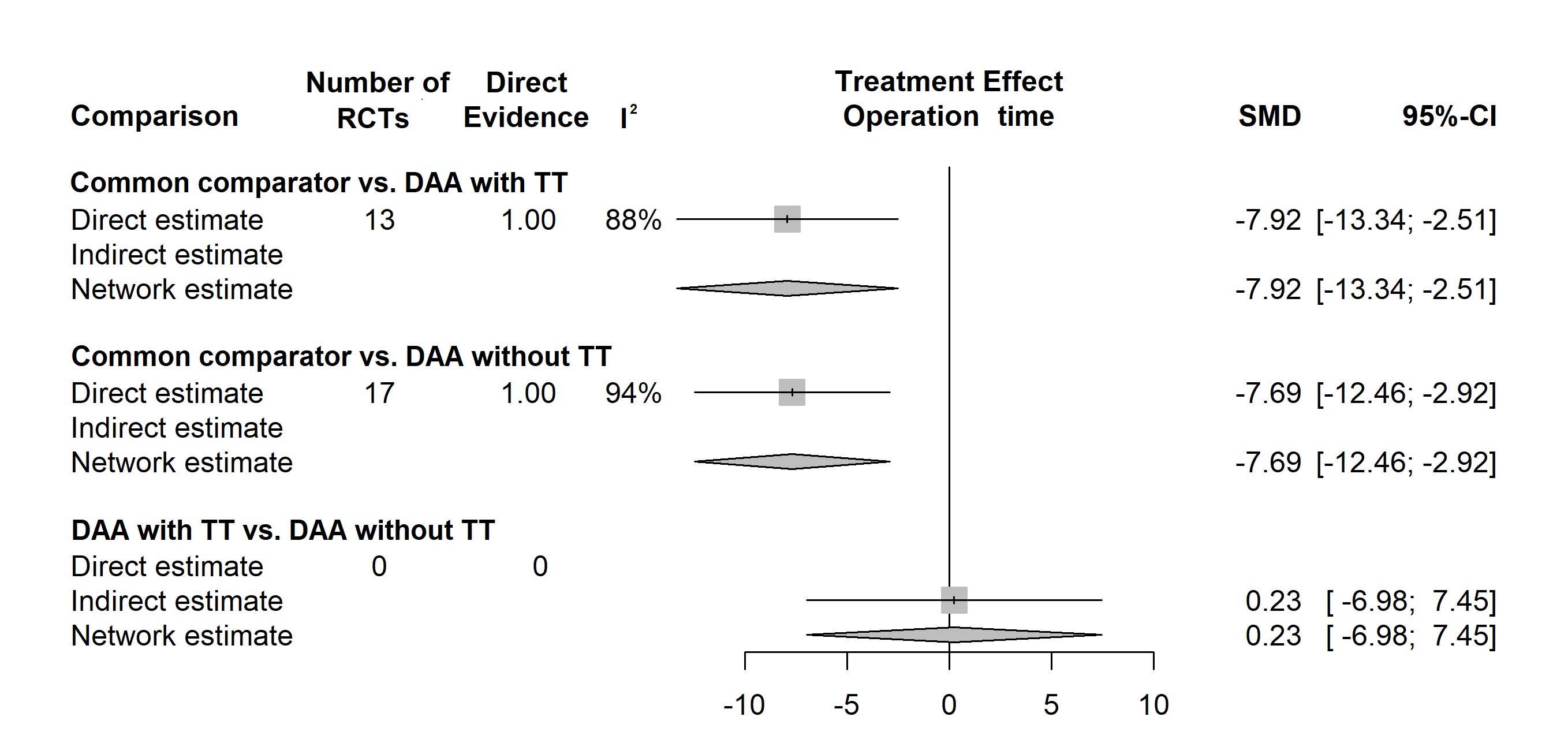

Supplement: Supplementary file 1 [file 13018_2024_4852_MOESM1_ESM.zip › Supplementary/Supplemental Figure 54 - Forest plot Sensitivity analysis Operation Time .jpg]

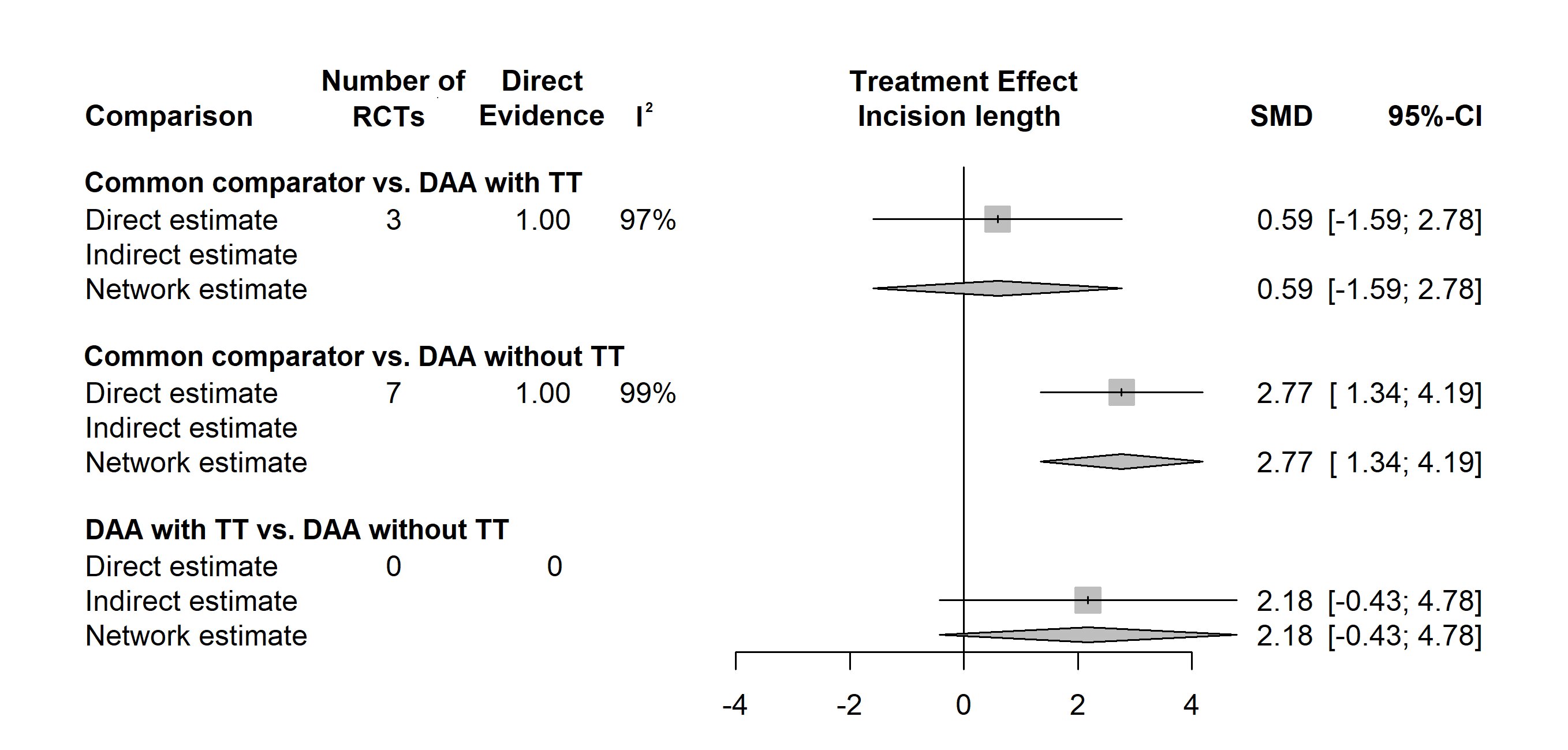

Supplement: Supplementary file 1 [file 13018_2024_4852_MOESM1_ESM.zip › Supplementary/Supplemental Figure 55 - Forest plot Sensitivity analysis Incision length.jpg]

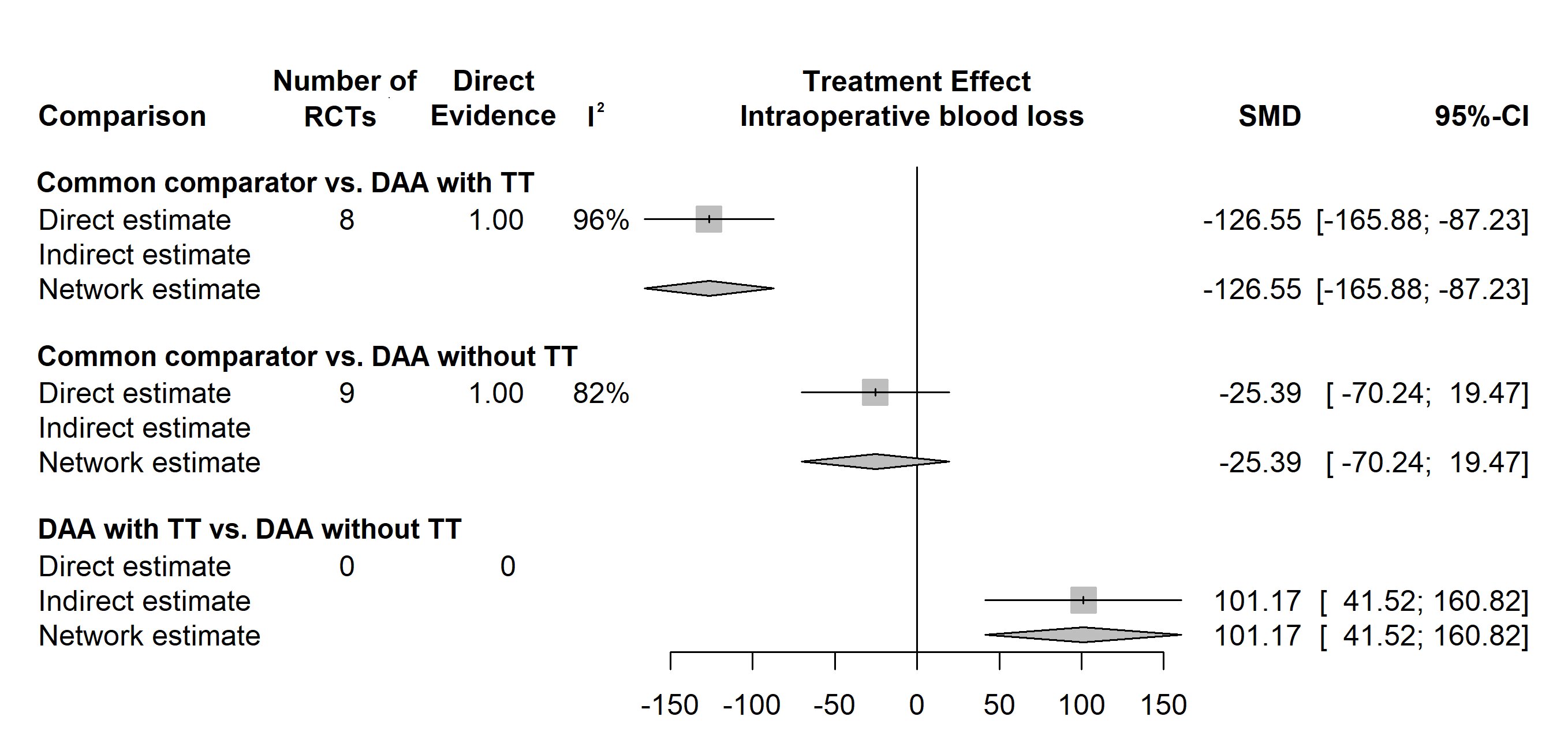

Supplement: Supplementary file 1 [file 13018_2024_4852_MOESM1_ESM.zip › Supplementary/Supplemental Figure 56 - Forest plot Sensitivity analysis Intraoperative blood loss.jpg]

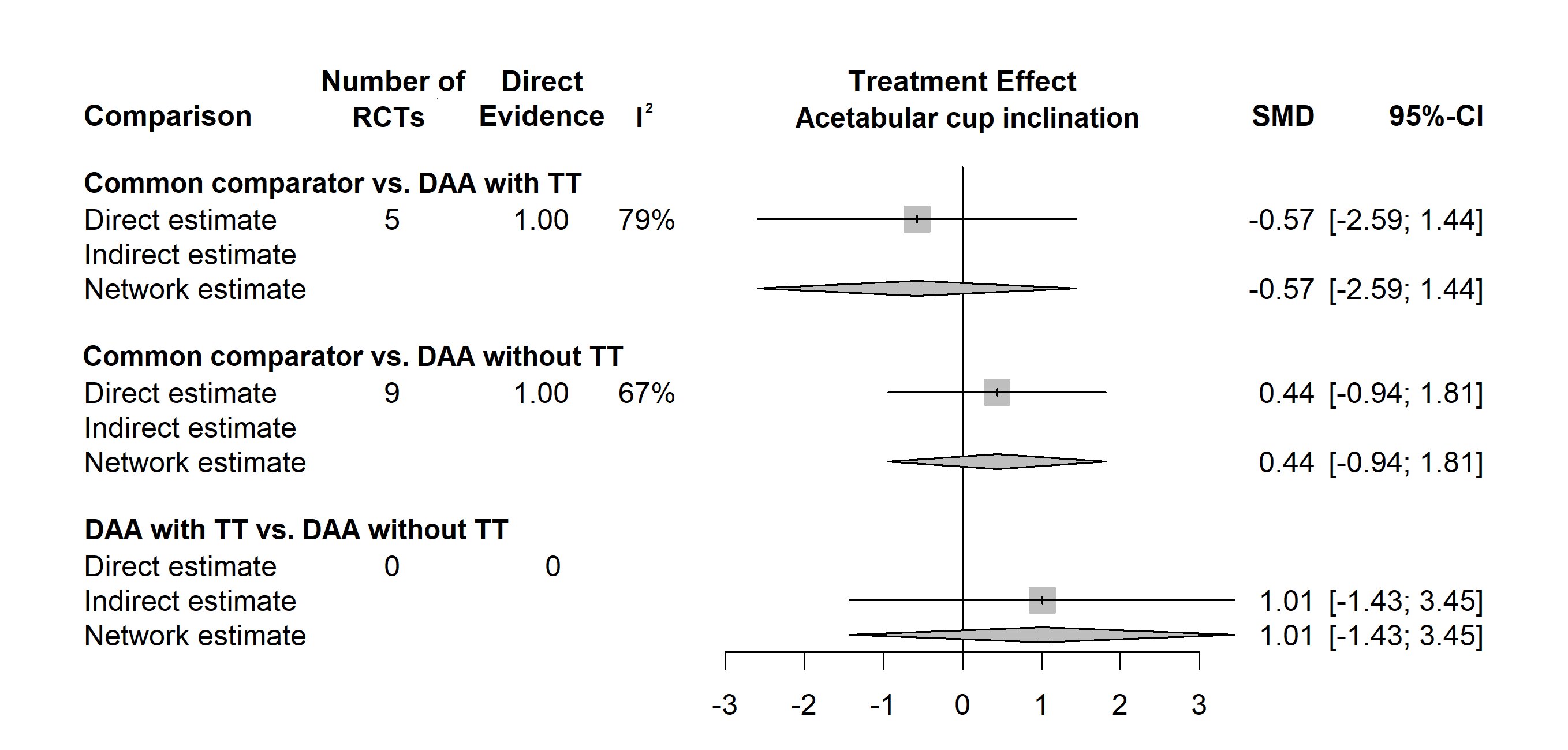

Supplement: Supplementary file 1 [file 13018_2024_4852_MOESM1_ESM.zip › Supplementary/Supplemental Figure 57 - Forest plot Sensitivity analysis Acetabular cup inclination.jpg]

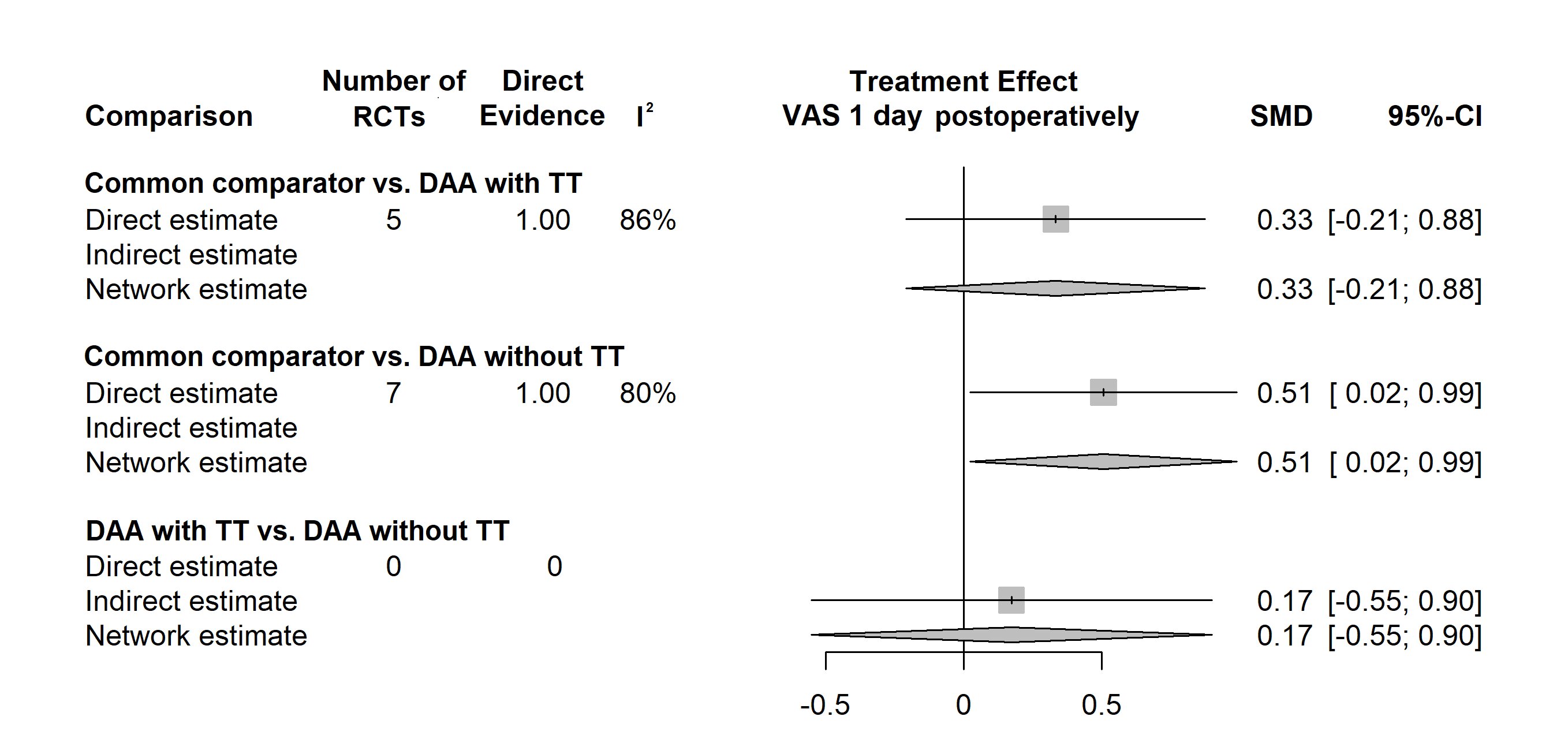

Supplement: Supplementary file 1 [file 13018_2024_4852_MOESM1_ESM.zip › Supplementary/Supplemental Figure 58 - Forest plot Sensitivity analysis VAS 1 day.jpg]

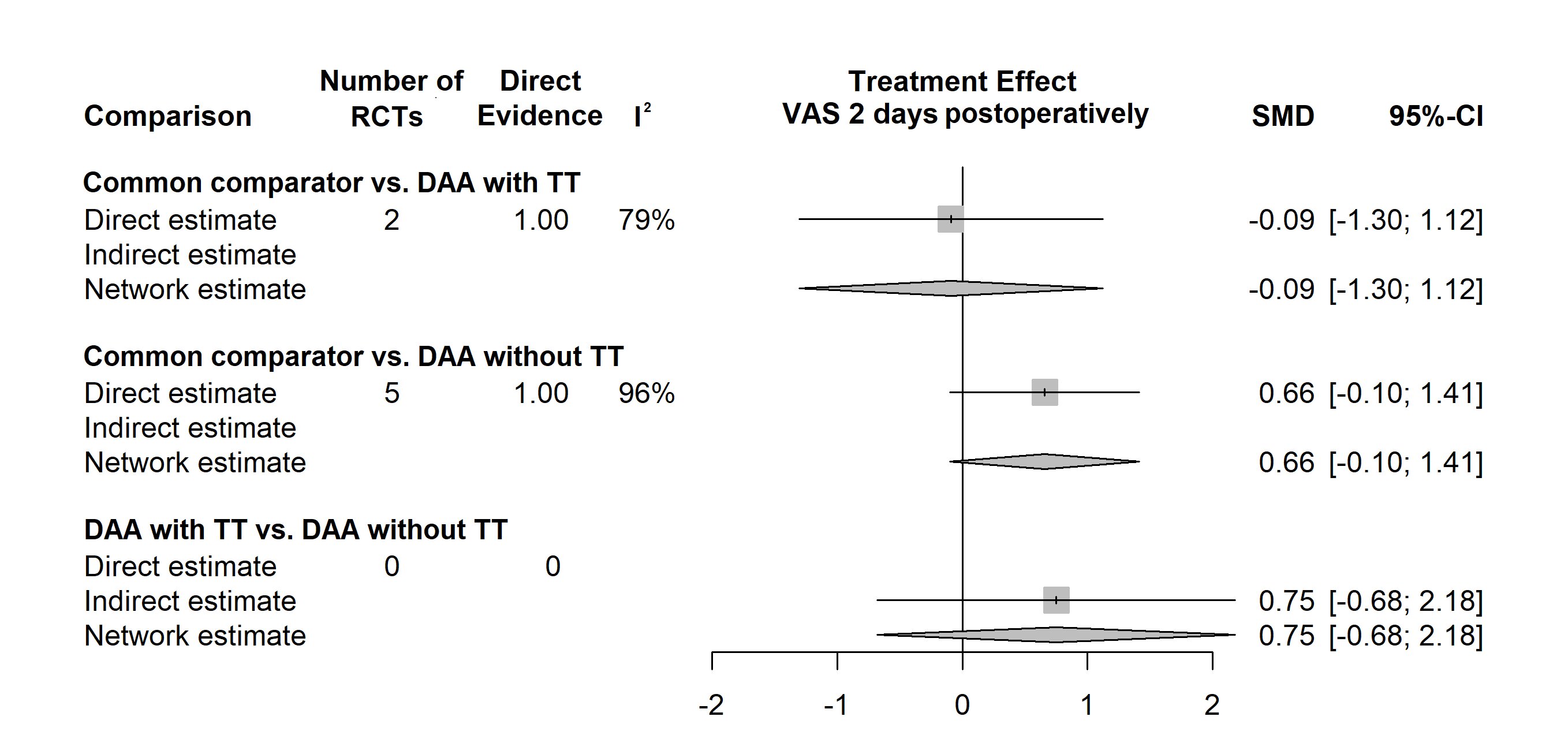

Supplement: Supplementary file 1 [file 13018_2024_4852_MOESM1_ESM.zip › Supplementary/Supplemental Figure 59 - Forest plot Sensitivity analysis VAS 2 days.jpg]

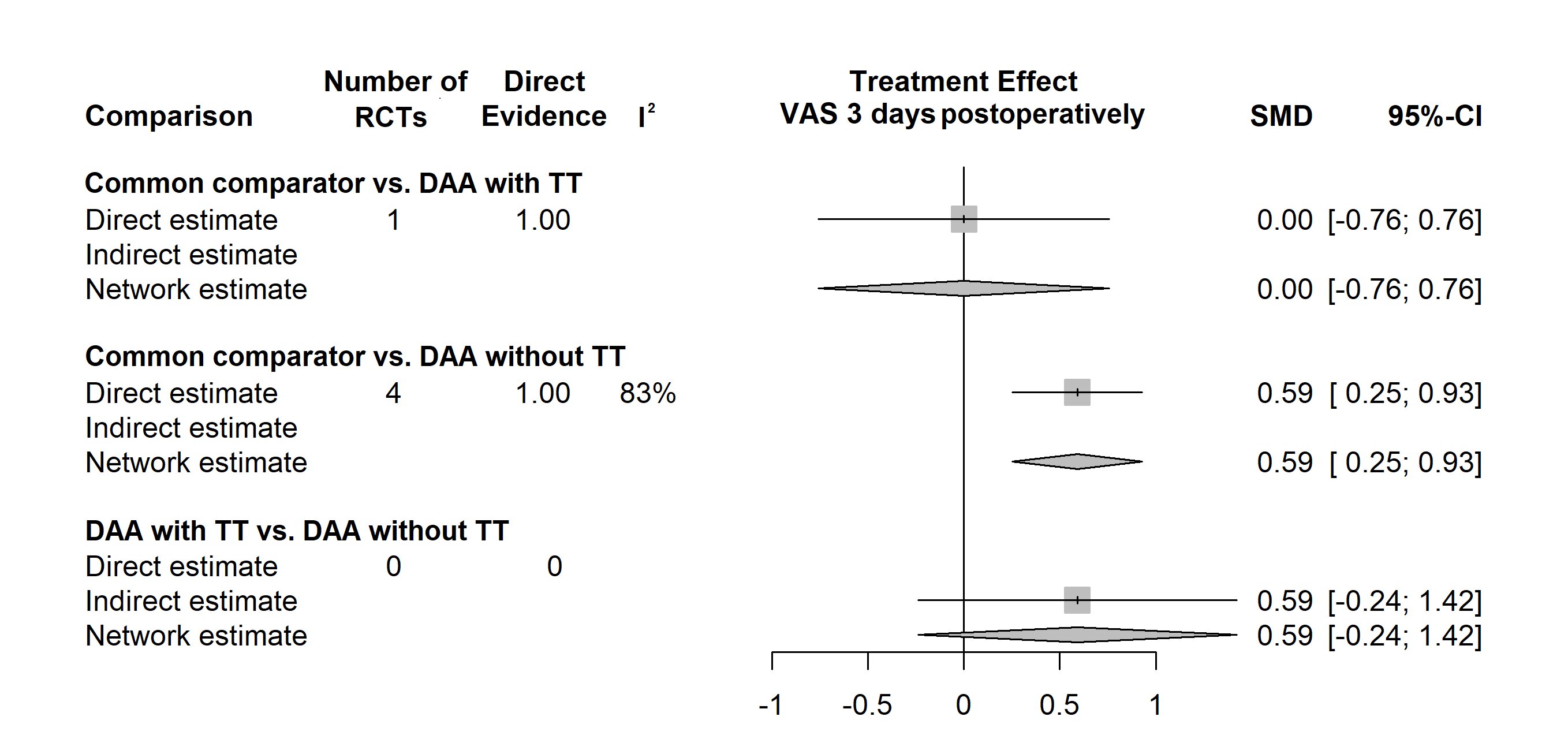

Supplement: Supplementary file 1 [file 13018_2024_4852_MOESM1_ESM.zip › Supplementary/Supplemental Figure 6 - Forest plot VAS 3 days.jpg]

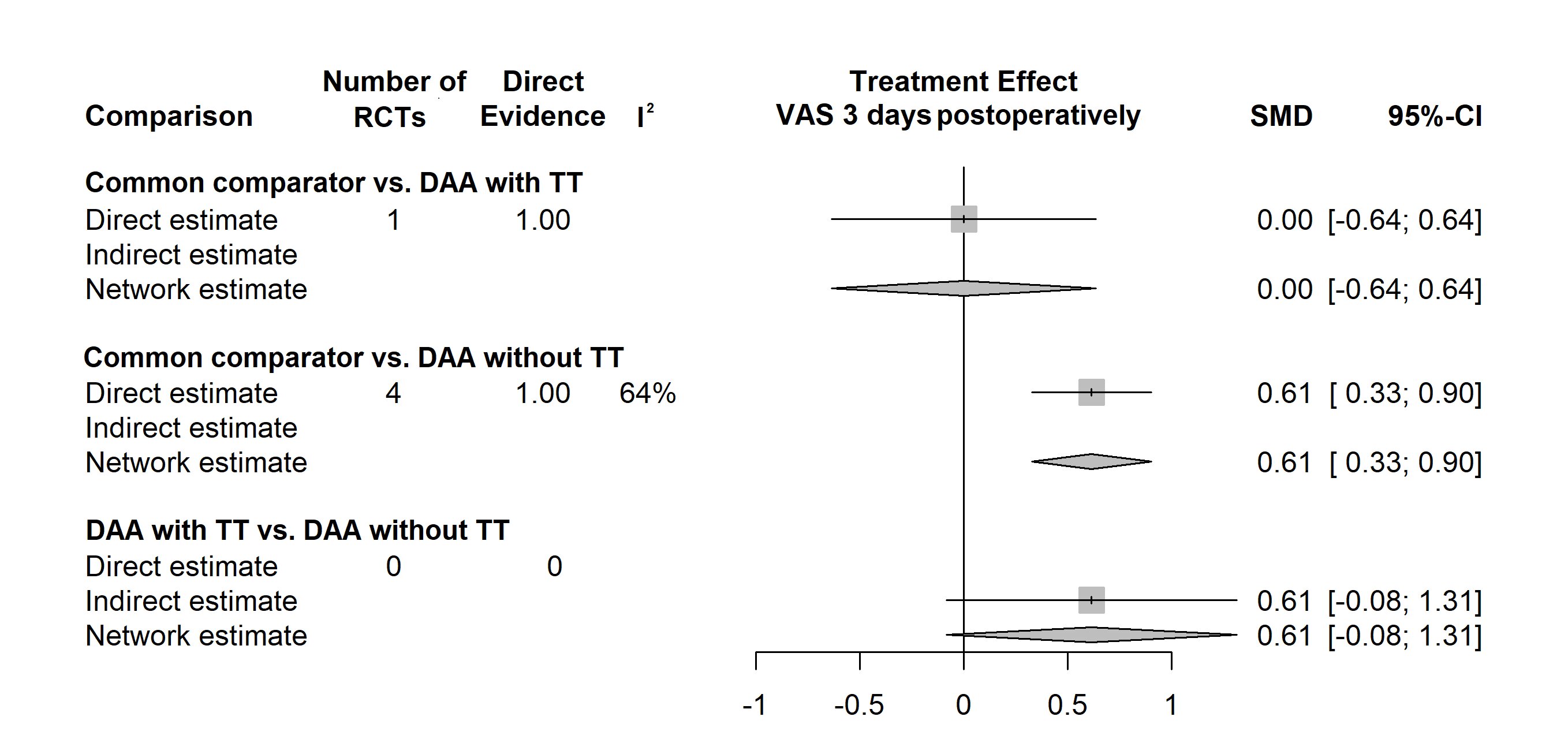

Supplement: Supplementary file 1 [file 13018_2024_4852_MOESM1_ESM.zip › Supplementary/Supplemental Figure 60 - Forest plot Sensitivity analysis VAS 3 days.jpg]

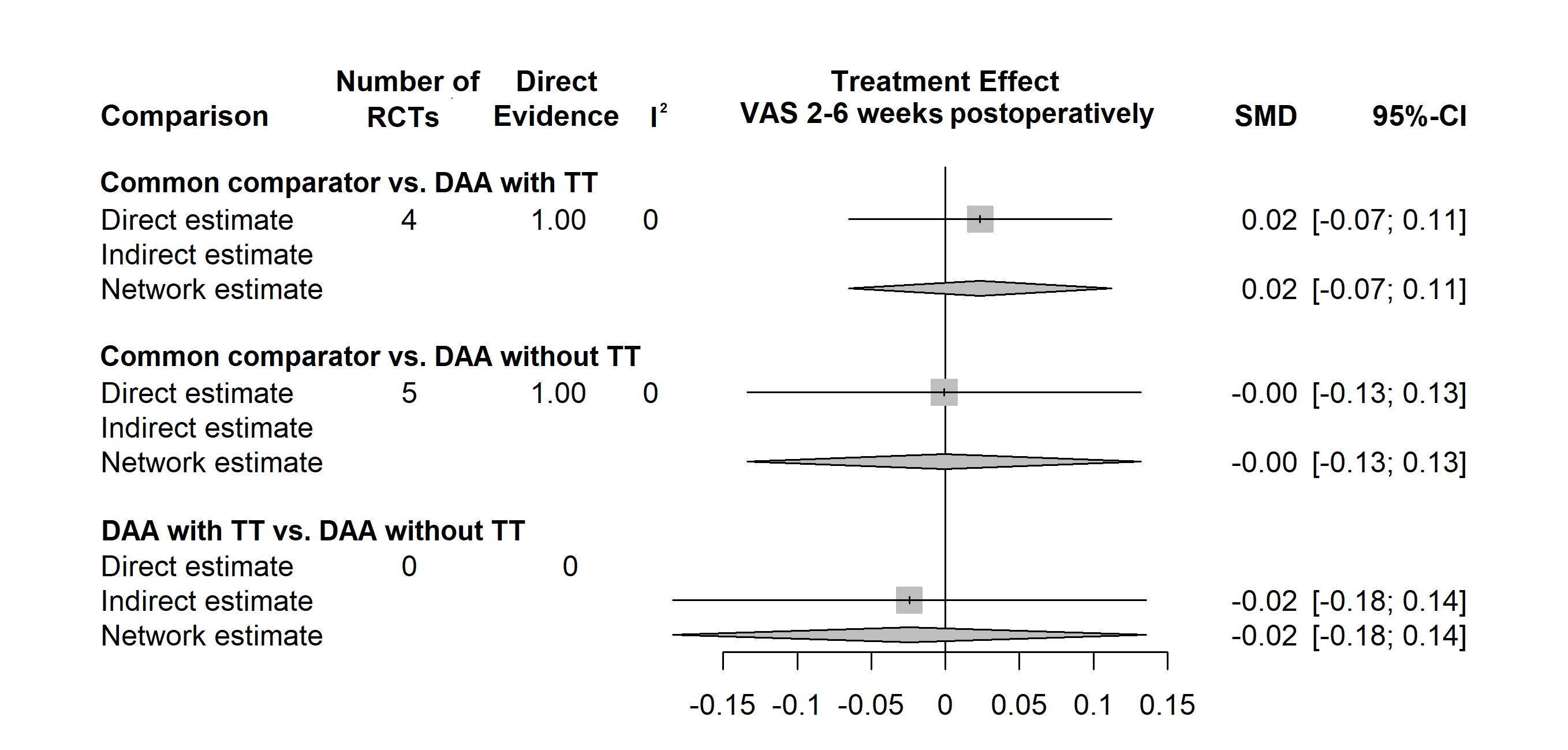

Supplement: Supplementary file 1 [file 13018_2024_4852_MOESM1_ESM.zip › Supplementary/Supplemental Figure 61 - Forest plot Sensitivity analysis VAS 2-6 week.jpg]

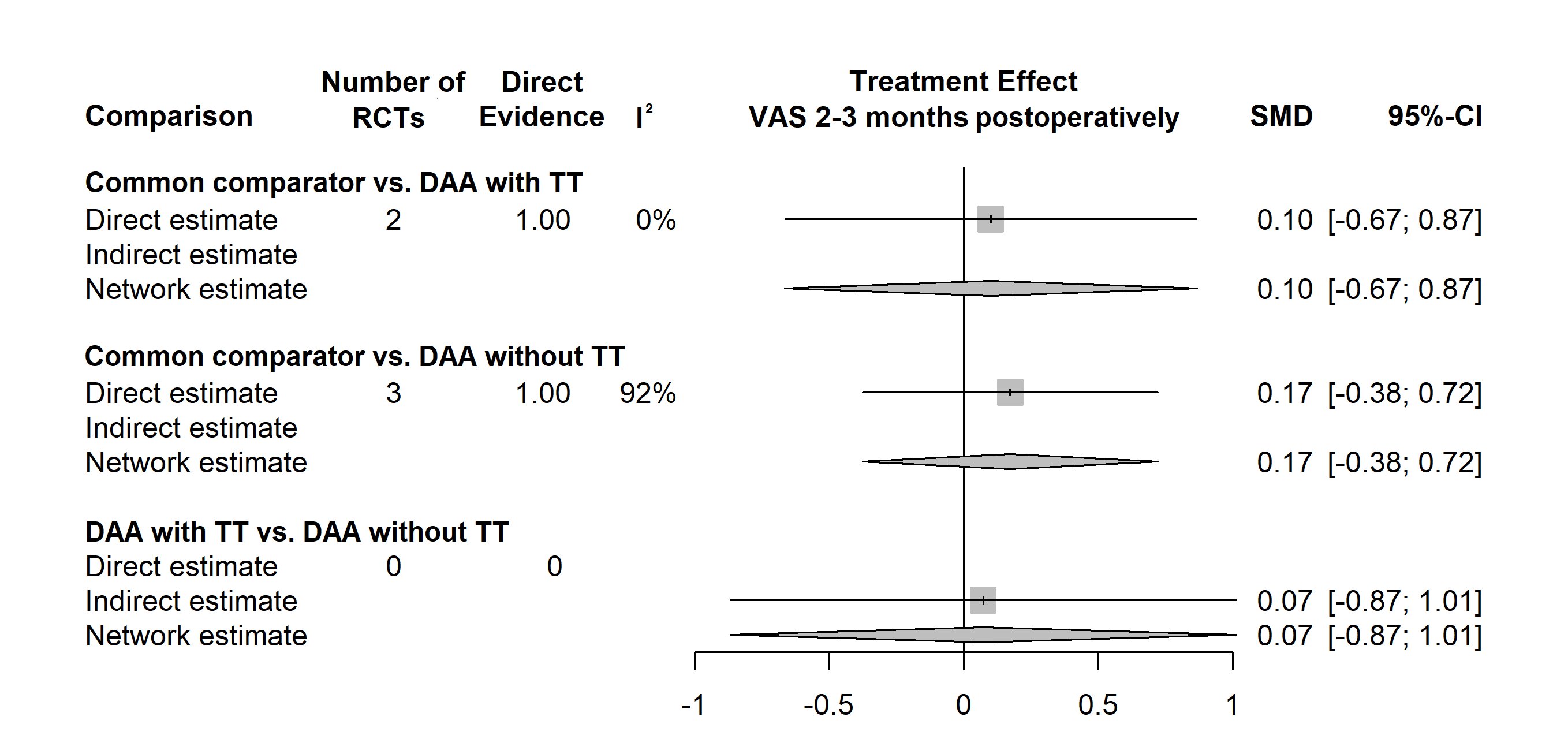

Supplement: Supplementary file 1 [file 13018_2024_4852_MOESM1_ESM.zip › Supplementary/Supplemental Figure 62 - Forest plot Sensitivity analysis VAS 2-3 months.jpg]

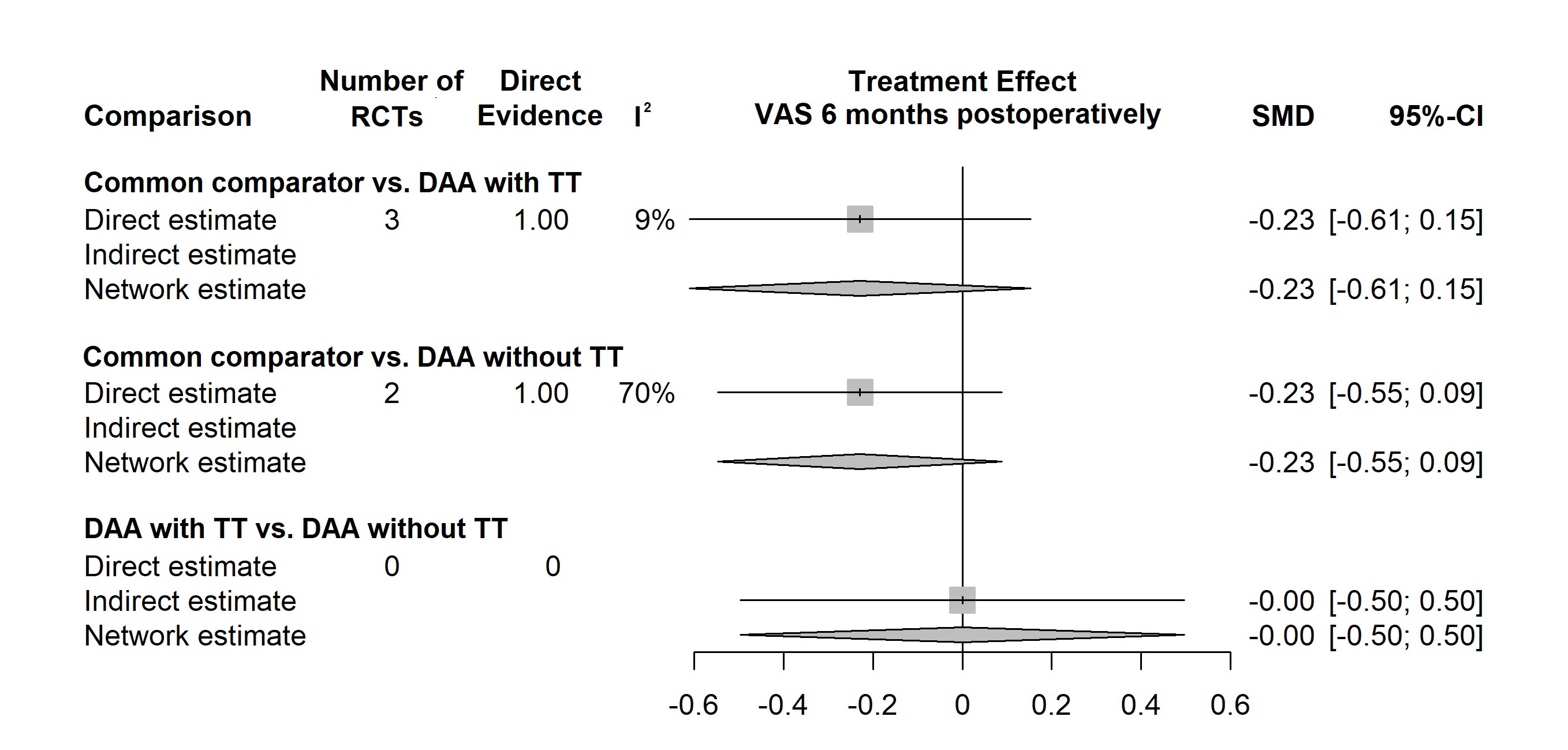

Supplement: Supplementary file 1 [file 13018_2024_4852_MOESM1_ESM.zip › Supplementary/Supplemental Figure 63 - Forest plot Sensitivity analysis VAS 6 months.jpg]

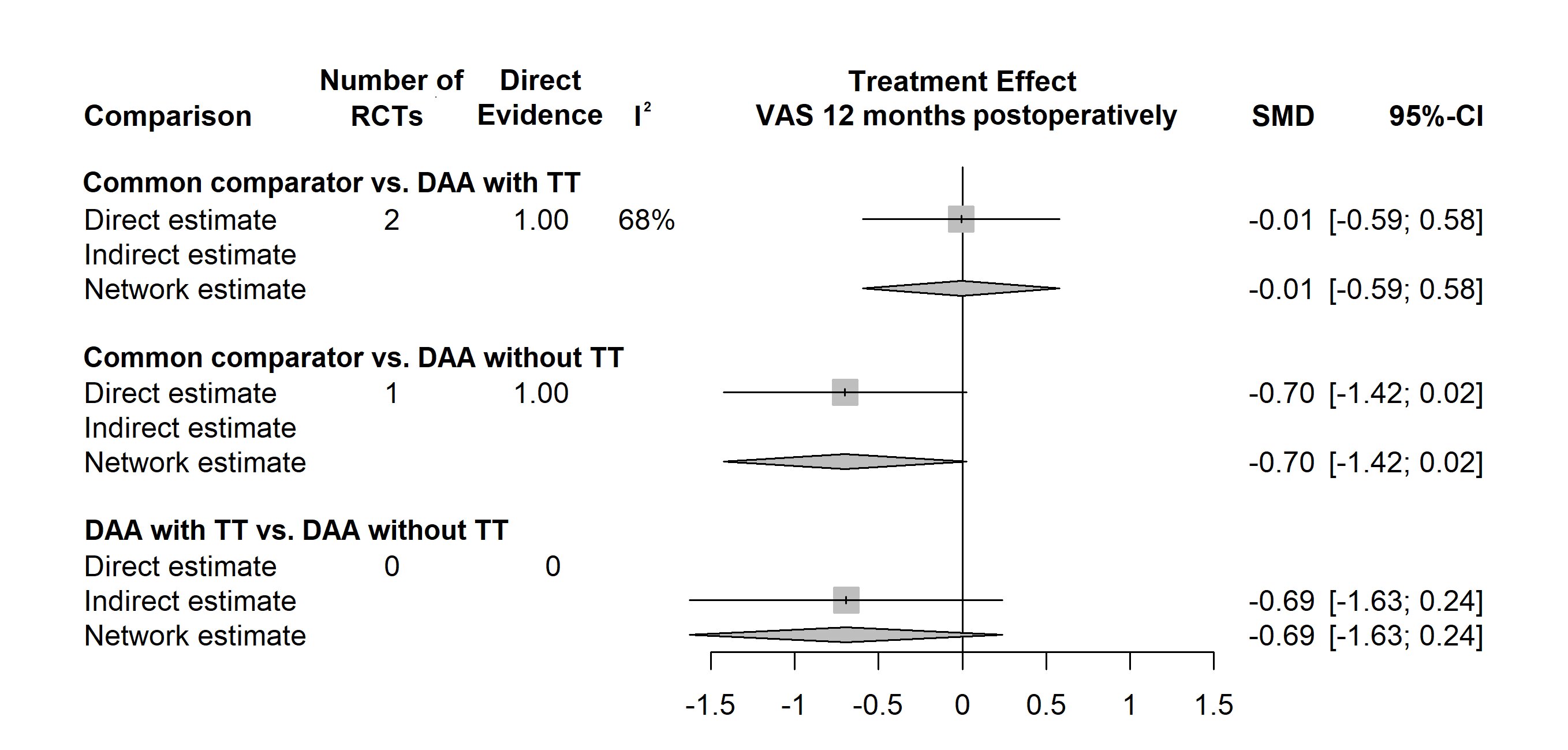

Supplement: Supplementary file 1 [file 13018_2024_4852_MOESM1_ESM.zip › Supplementary/Supplemental Figure 64 - Forest plot Sensitivity analysis VAS 12 months.jpg]

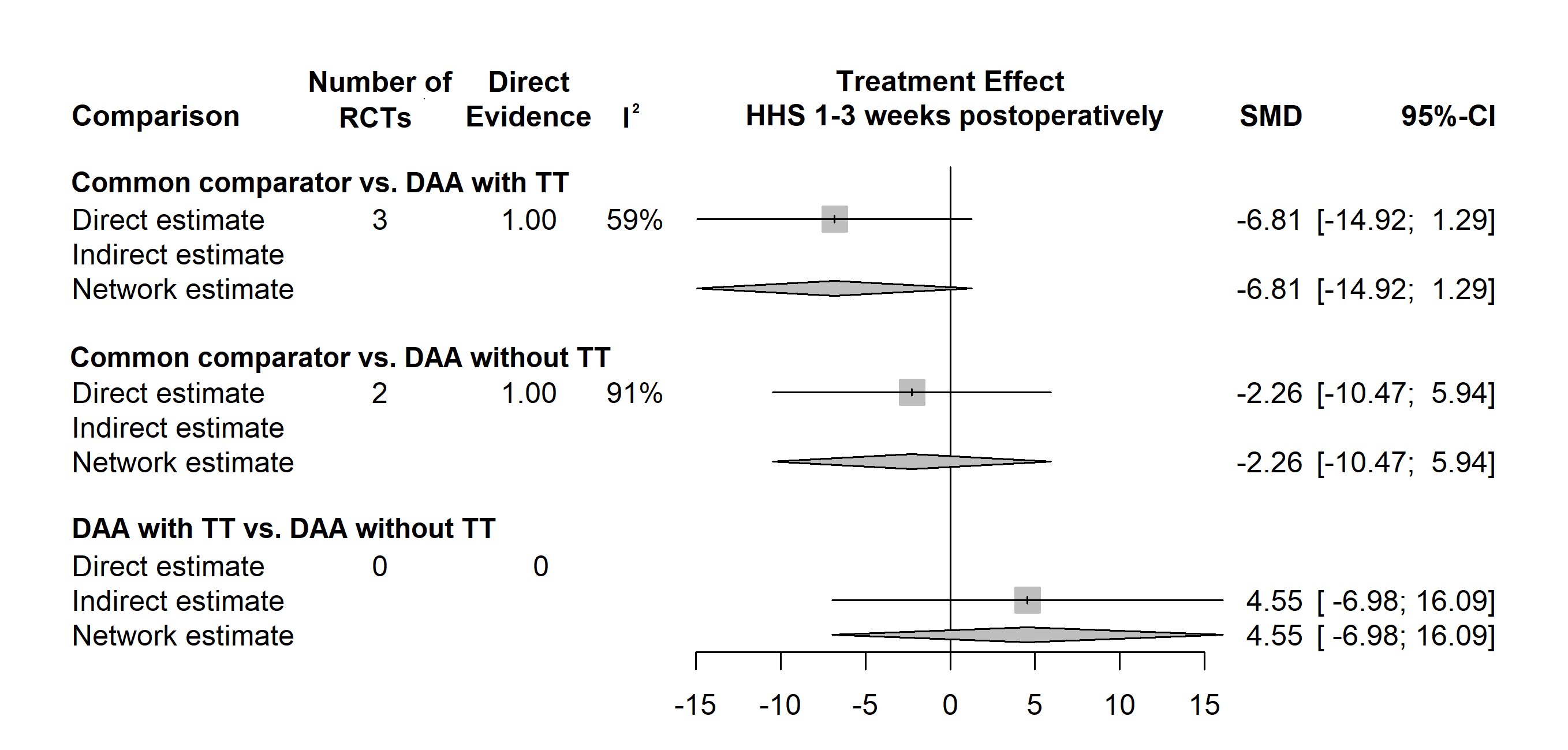

Supplement: Supplementary file 1 [file 13018_2024_4852_MOESM1_ESM.zip › Supplementary/Supplemental Figure 65 - Forest plot Sensitivity analysis HHS 1-3 weeks.jpg]

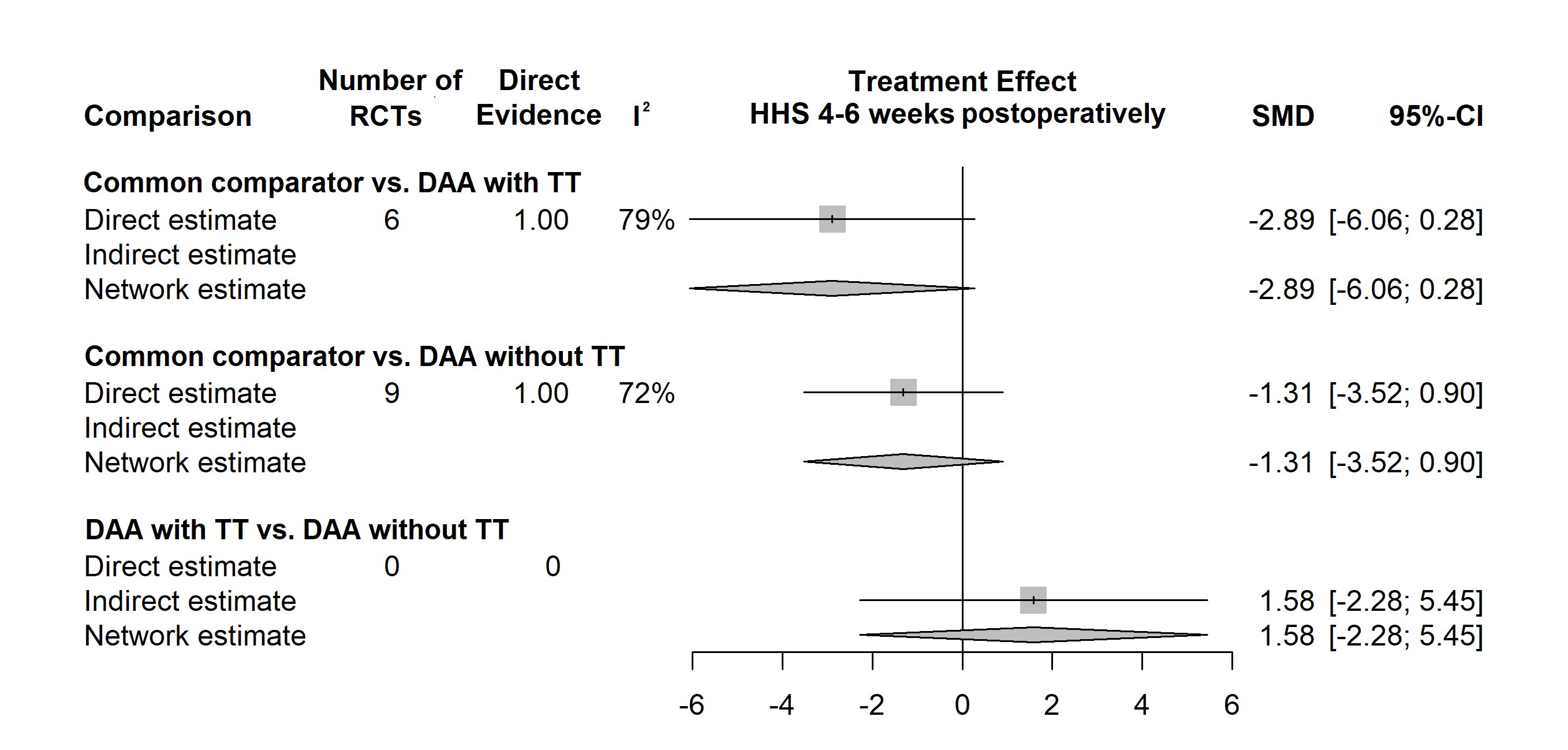

Supplement: Supplementary file 1 [file 13018_2024_4852_MOESM1_ESM.zip › Supplementary/Supplemental Figure 66 - Forest plot Sensitivity analysis HHS 4-6 weeks.jpg]

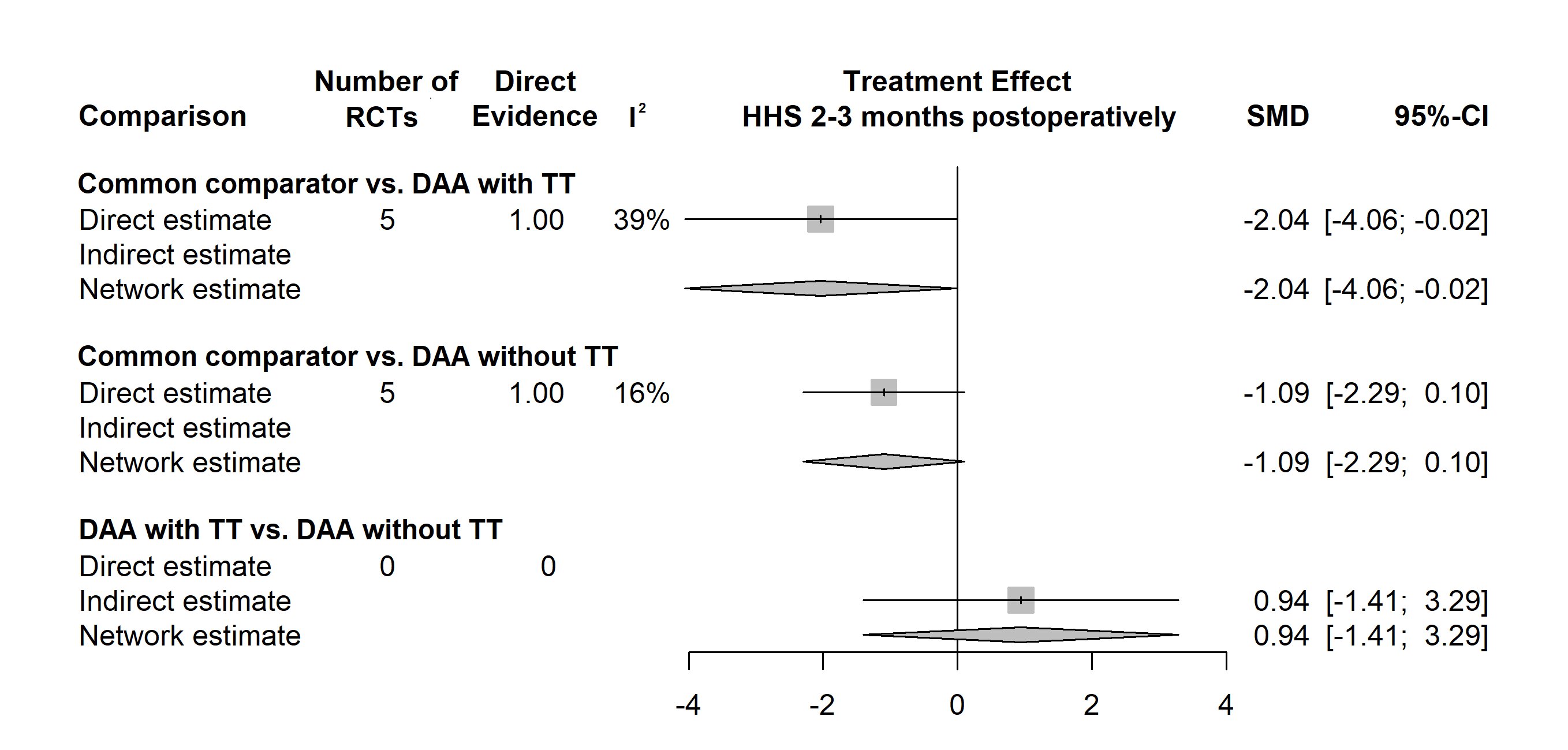

Supplement: Supplementary file 1 [file 13018_2024_4852_MOESM1_ESM.zip › Supplementary/Supplemental Figure 67 - Forest plot Sensitivity analysis HHS 2-3 months.jpg]

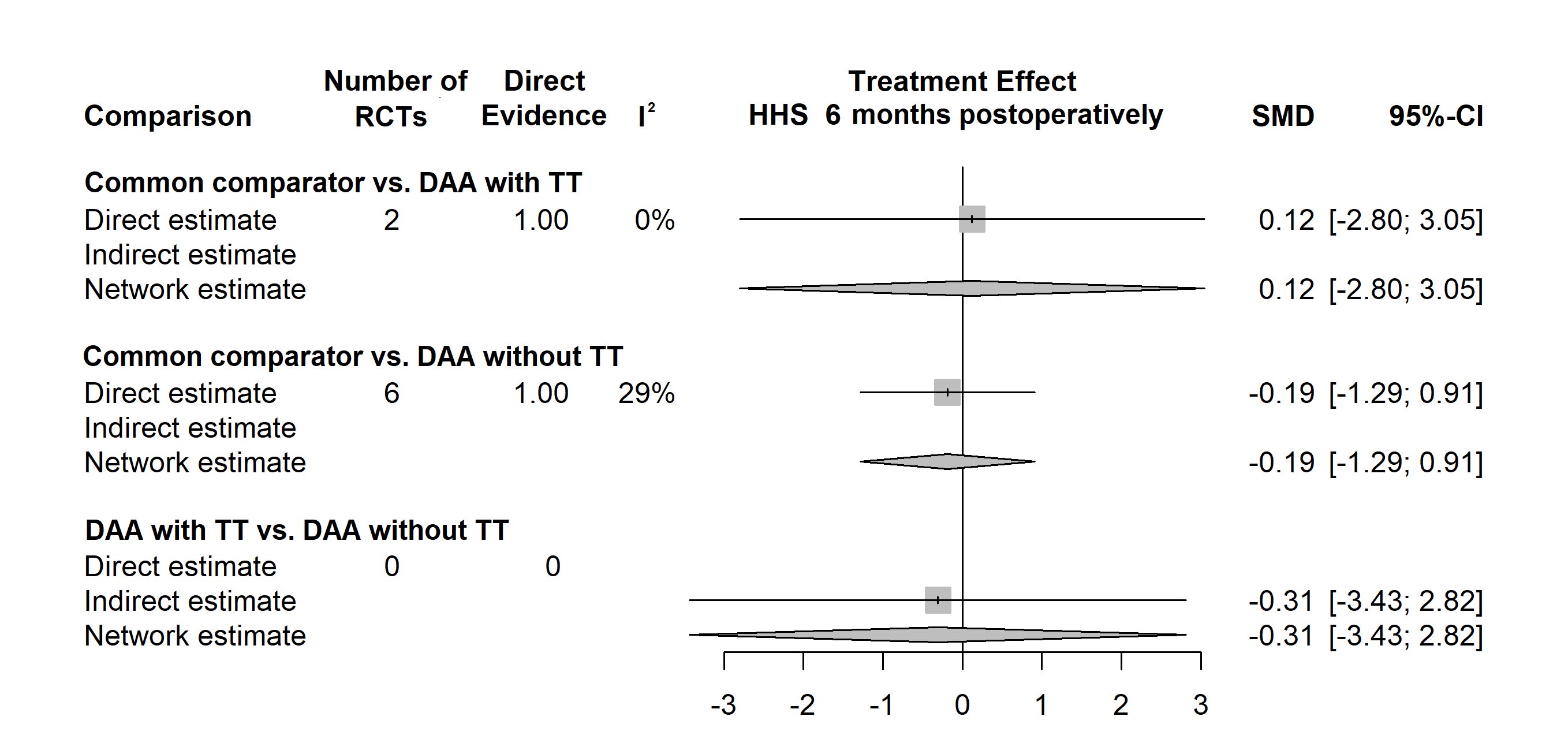

Supplement: Supplementary file 1 [file 13018_2024_4852_MOESM1_ESM.zip › Supplementary/Supplemental Figure 68 - Forest plot Sensitivity analysis HHS 6 months.jpg]

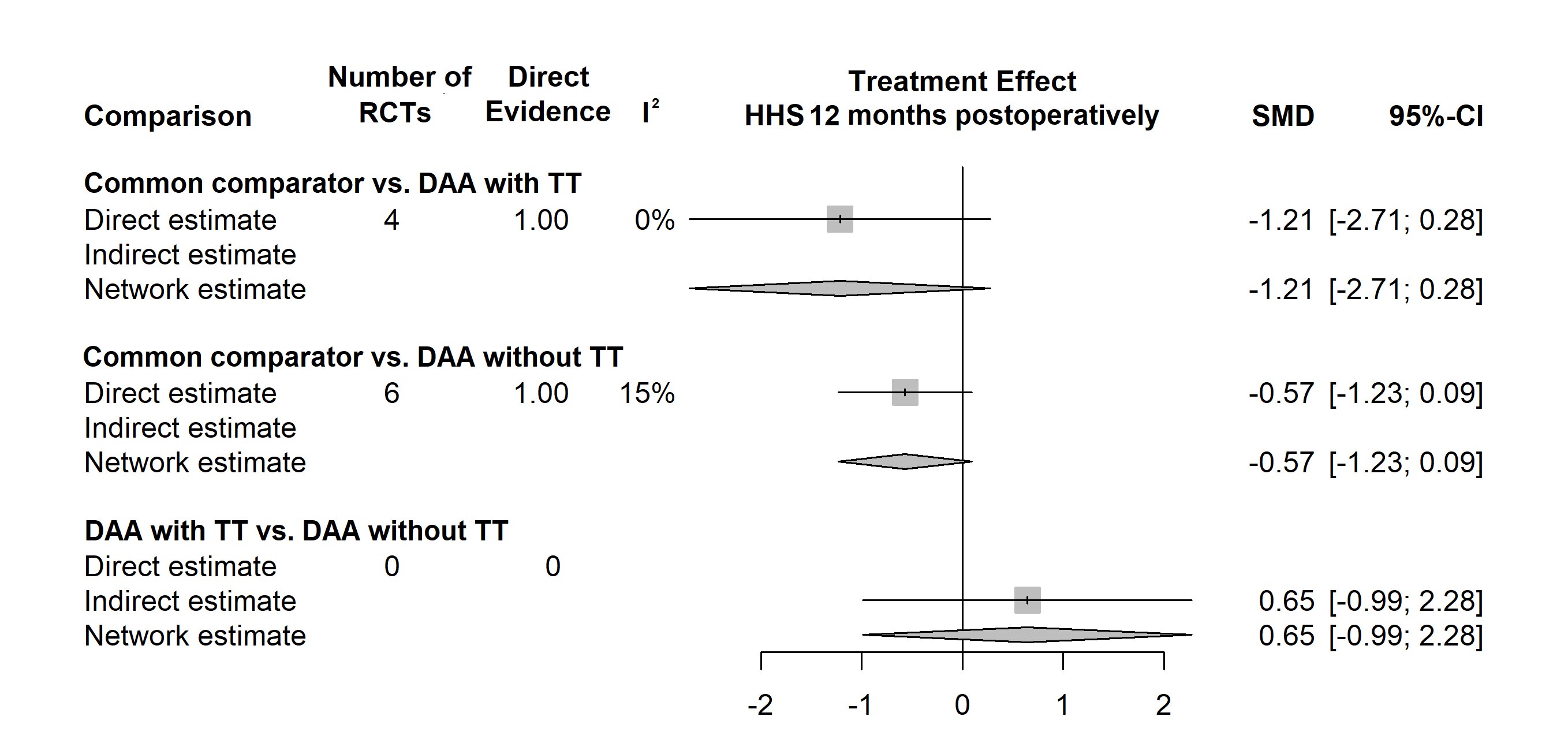

Supplement: Supplementary file 1 [file 13018_2024_4852_MOESM1_ESM.zip › Supplementary/Supplemental Figure 69 - Forest plot Sensitivity analysis HHS 12 months.jpg]

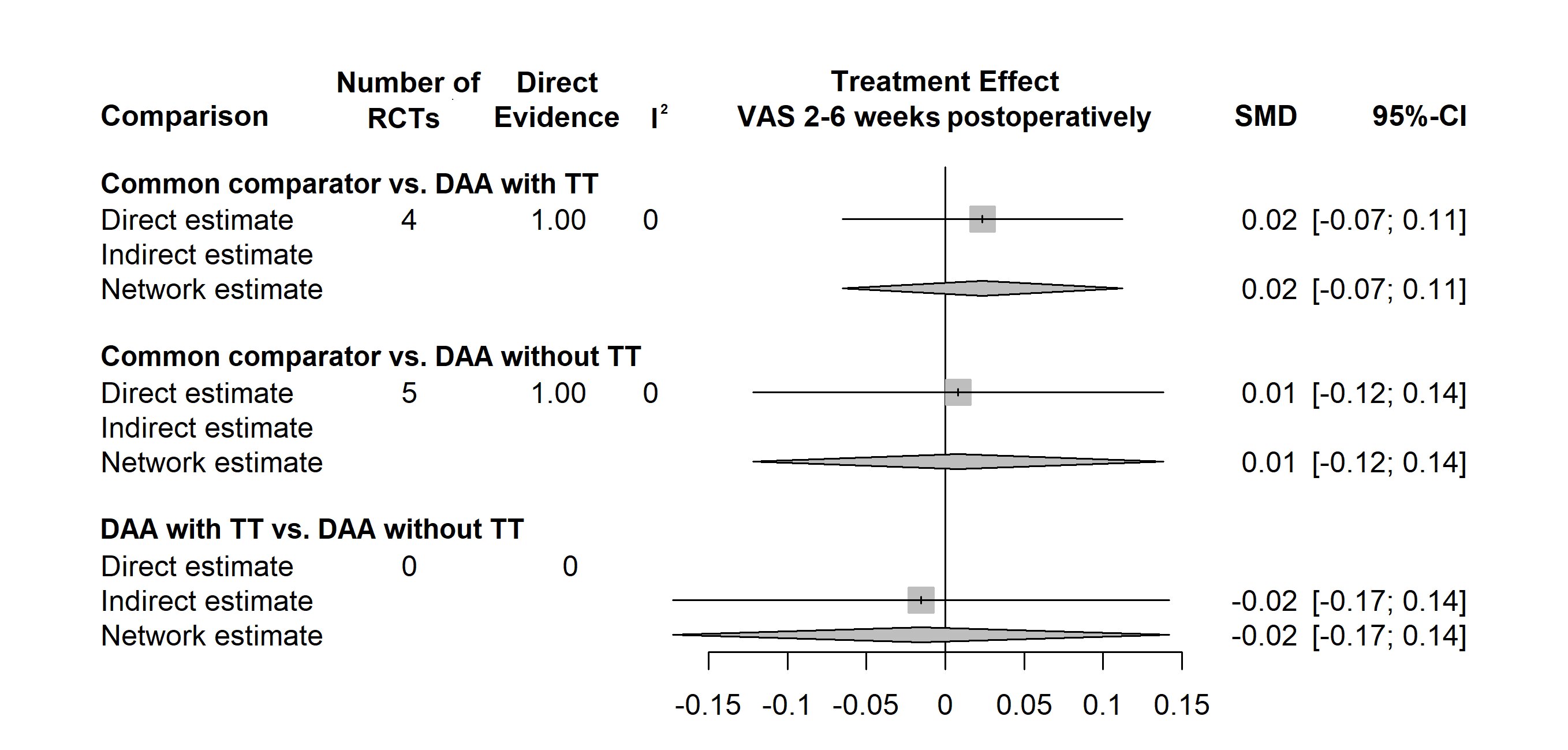

Supplement: Supplementary file 1 [file 13018_2024_4852_MOESM1_ESM.zip › Supplementary/Supplemental Figure 7 - Forest plot VAS 2-6 week.jpg]

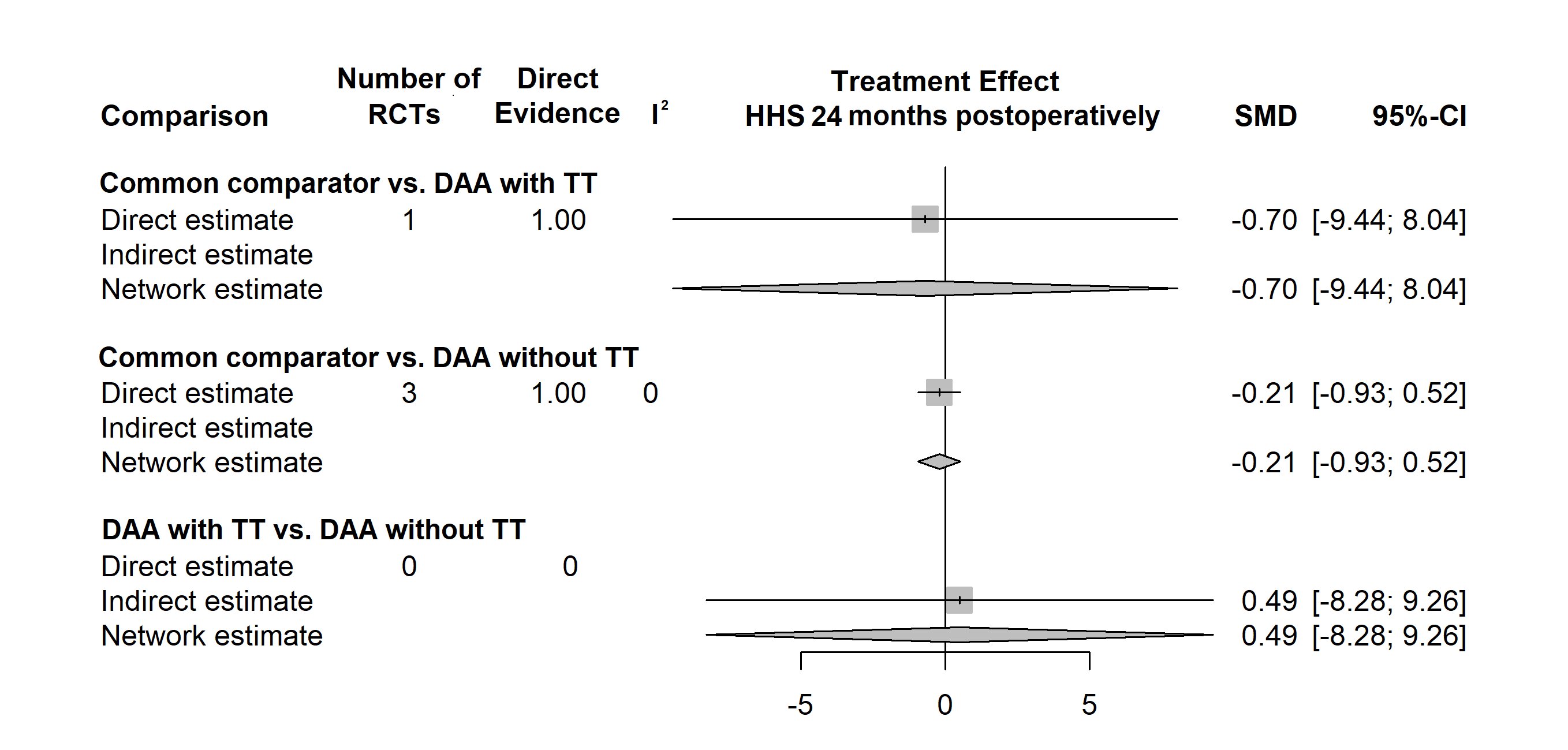

Supplement: Supplementary file 1 [file 13018_2024_4852_MOESM1_ESM.zip › Supplementary/Supplemental Figure 70 - Forest plot Sensitivity analysis HHS 24 months.jpg]

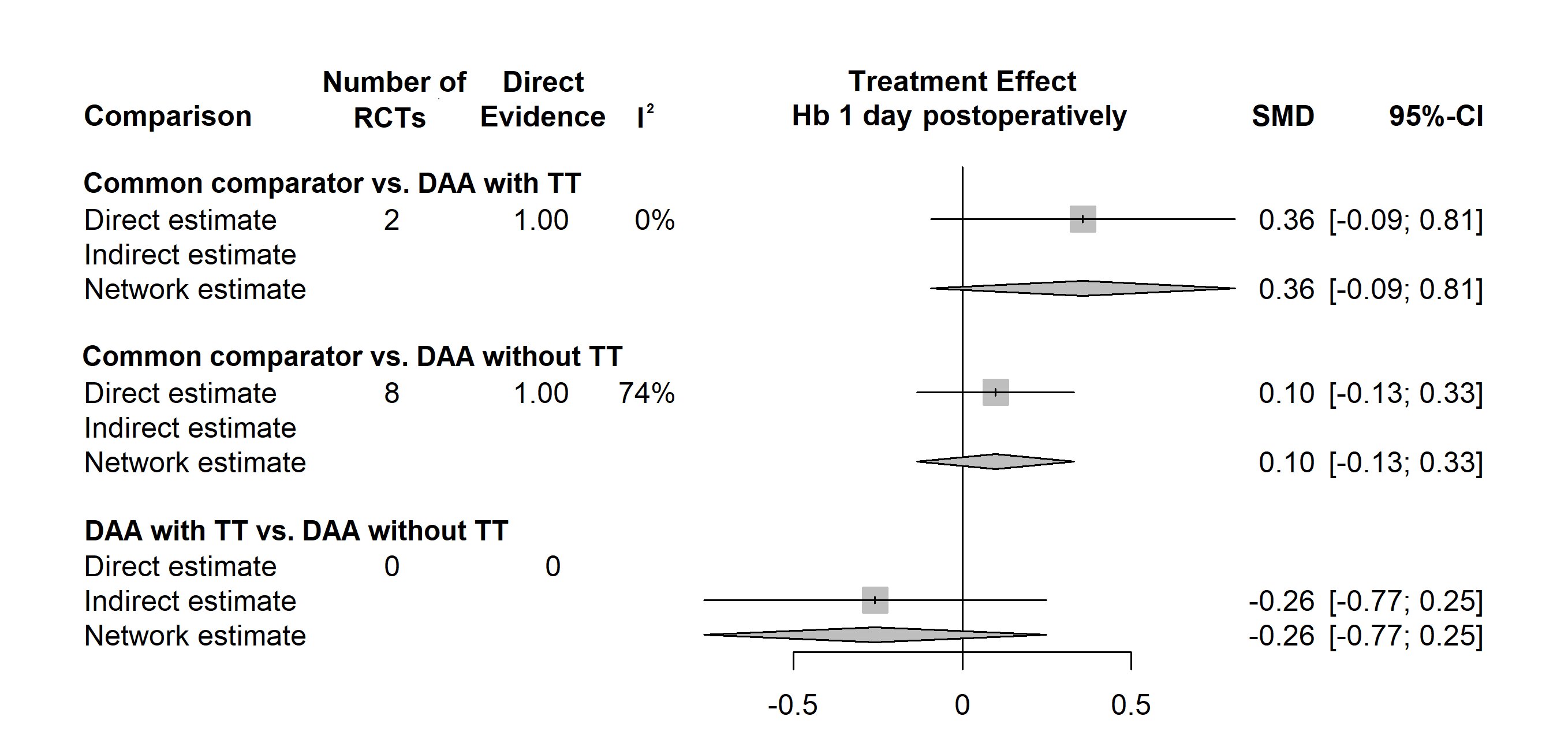

Supplement: Supplementary file 1 [file 13018_2024_4852_MOESM1_ESM.zip › Supplementary/Supplemental Figure 71 - Forest plot Sensitivity analysis Hb 1 day.jpg]

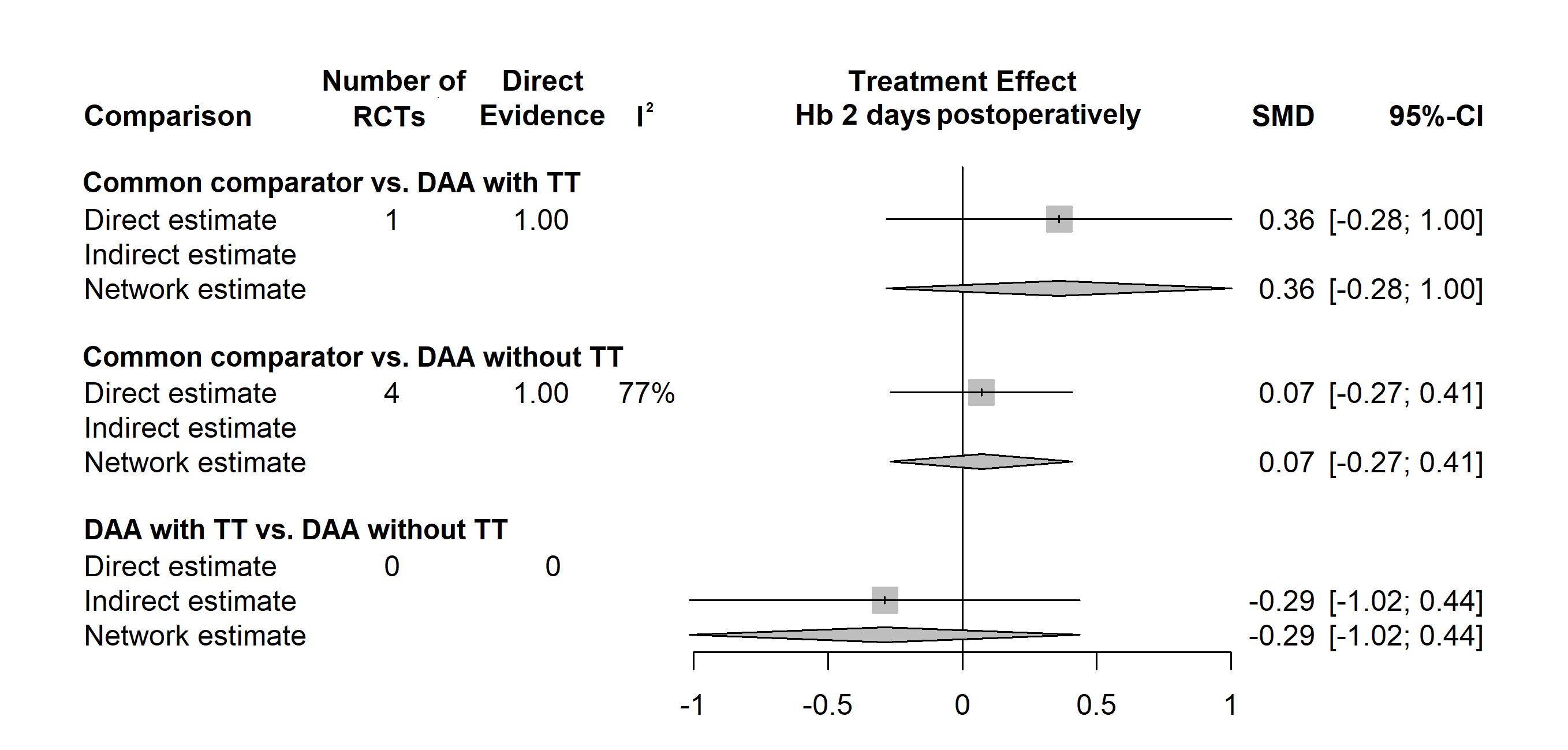

Supplement: Supplementary file 1 [file 13018_2024_4852_MOESM1_ESM.zip › Supplementary/Supplemental Figure 72 - Forest plot Sensitivity analysis Hb 2 days.jpg]

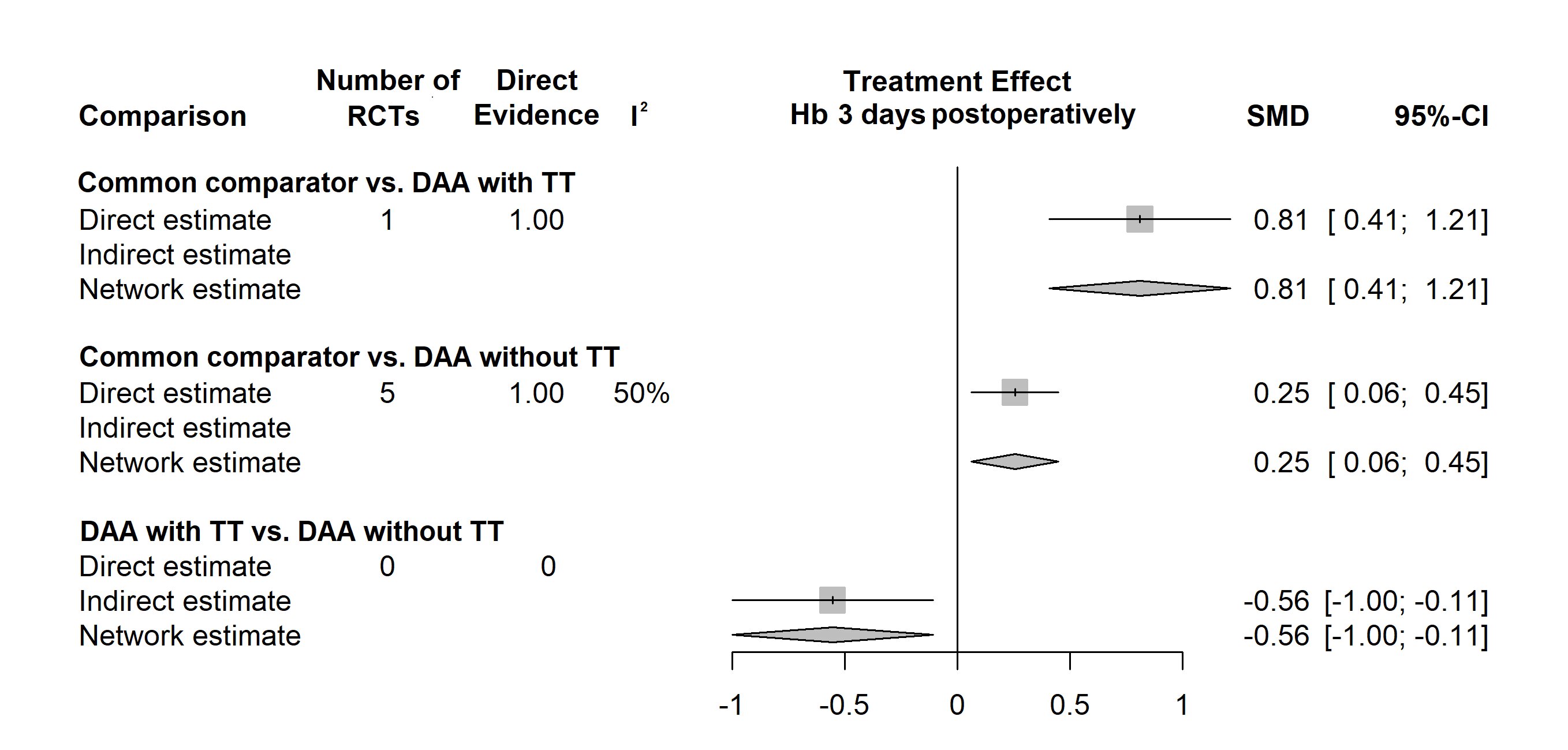

Supplement: Supplementary file 1 [file 13018_2024_4852_MOESM1_ESM.zip › Supplementary/Supplemental Figure 73 - Forest plot Sensitivity analysis Hb 3 days.jpg]

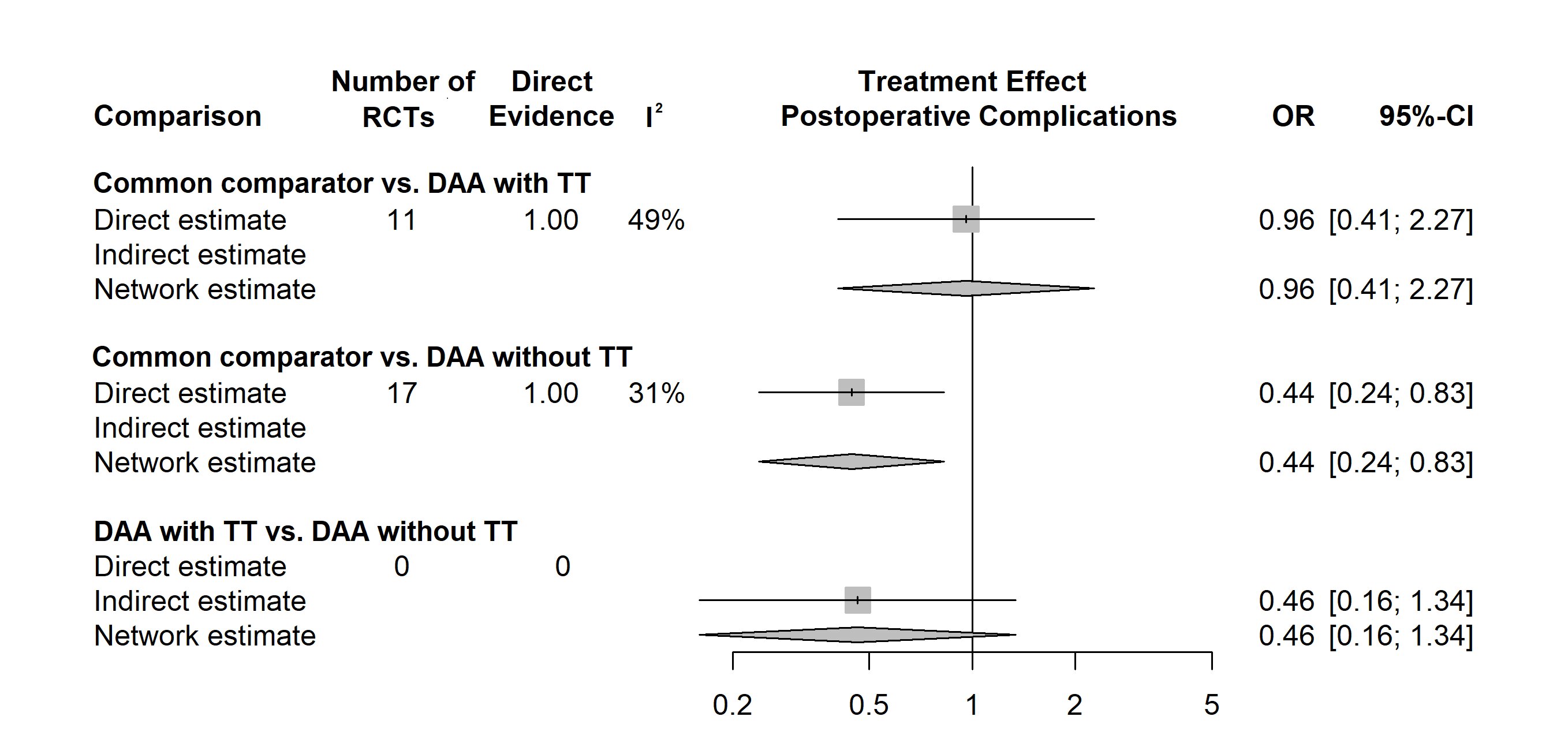

Supplement: Supplementary file 1 [file 13018_2024_4852_MOESM1_ESM.zip › Supplementary/Supplemental Figure 74 - Forest plot Sensitivity analysis Postoperative Complications.jpg]

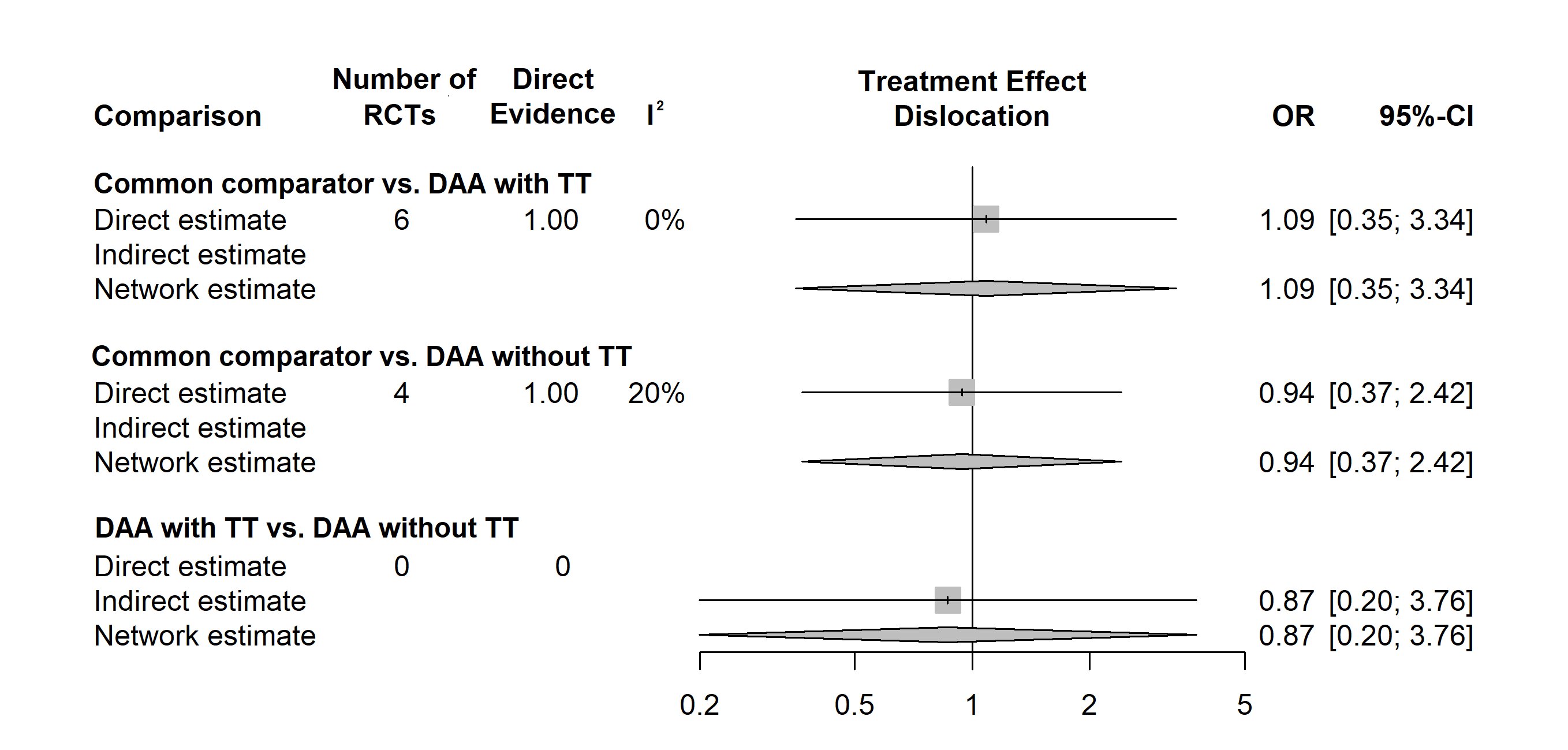

Supplement: Supplementary file 1 [file 13018_2024_4852_MOESM1_ESM.zip › Supplementary/Supplemental Figure 75 - Forest plot Sensitivity analysis Dislocation.jpg]

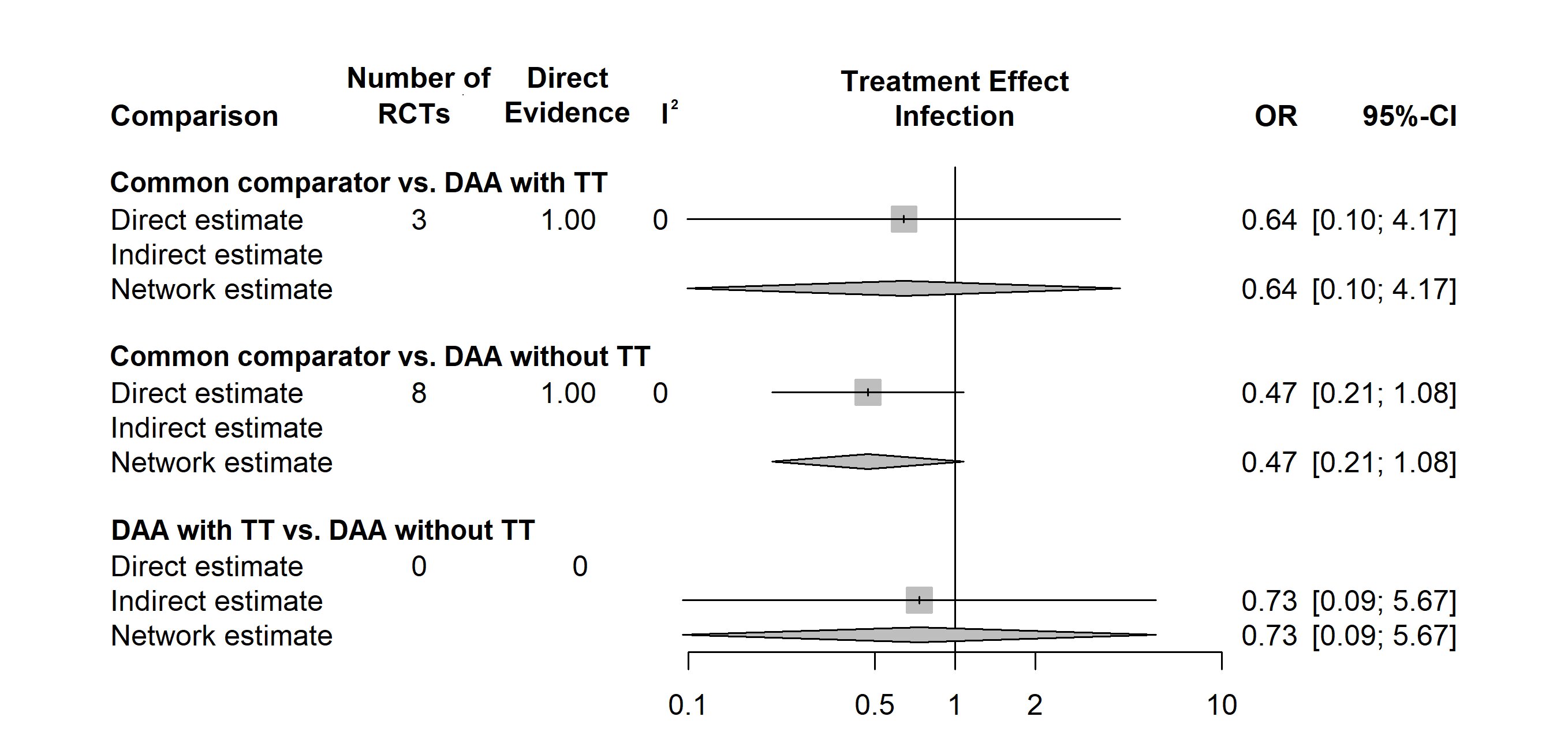

Supplement: Supplementary file 1 [file 13018_2024_4852_MOESM1_ESM.zip › Supplementary/Supplemental Figure 76 - Forest plot Sensitivity analysis Infection.jpg]

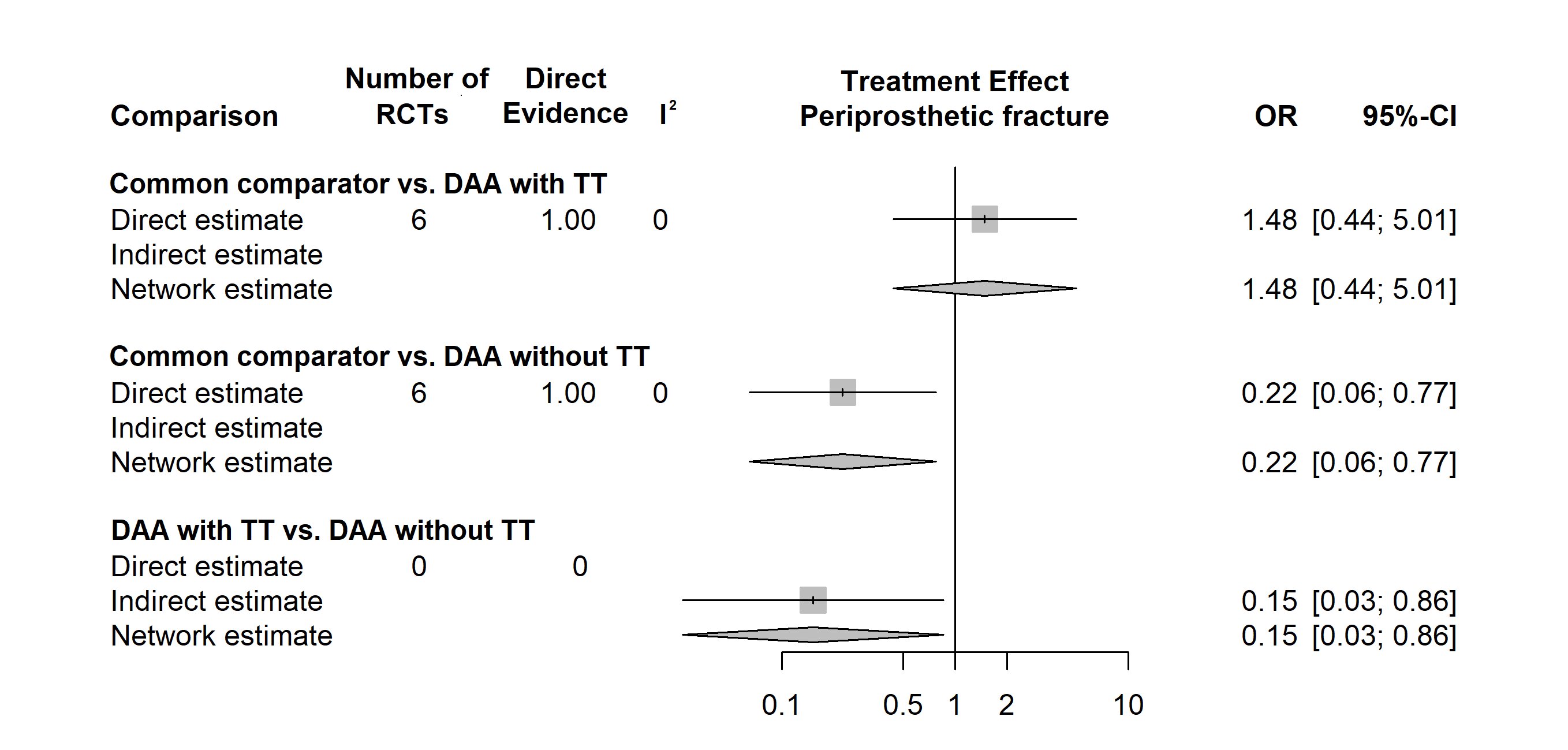

Supplement: Supplementary file 1 [file 13018_2024_4852_MOESM1_ESM.zip › Supplementary/Supplemental Figure 77 - Forest plot Sensitivity analysis Periprosthetic fracture.jpg]

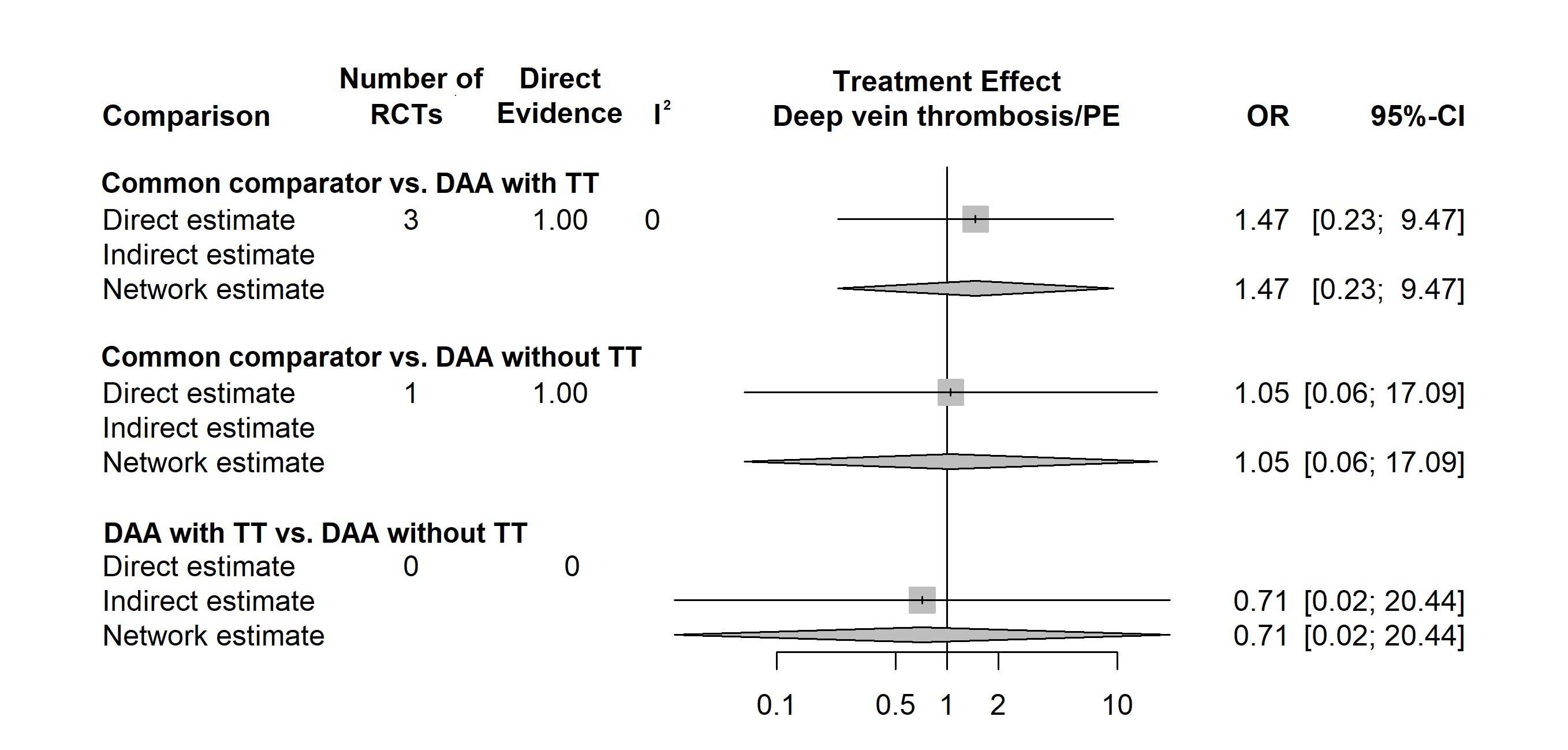

Supplement: Supplementary file 1 [file 13018_2024_4852_MOESM1_ESM.zip › Supplementary/Supplemental Figure 78 - Forest plot Sensitivity analysis DVT PE.jpg]

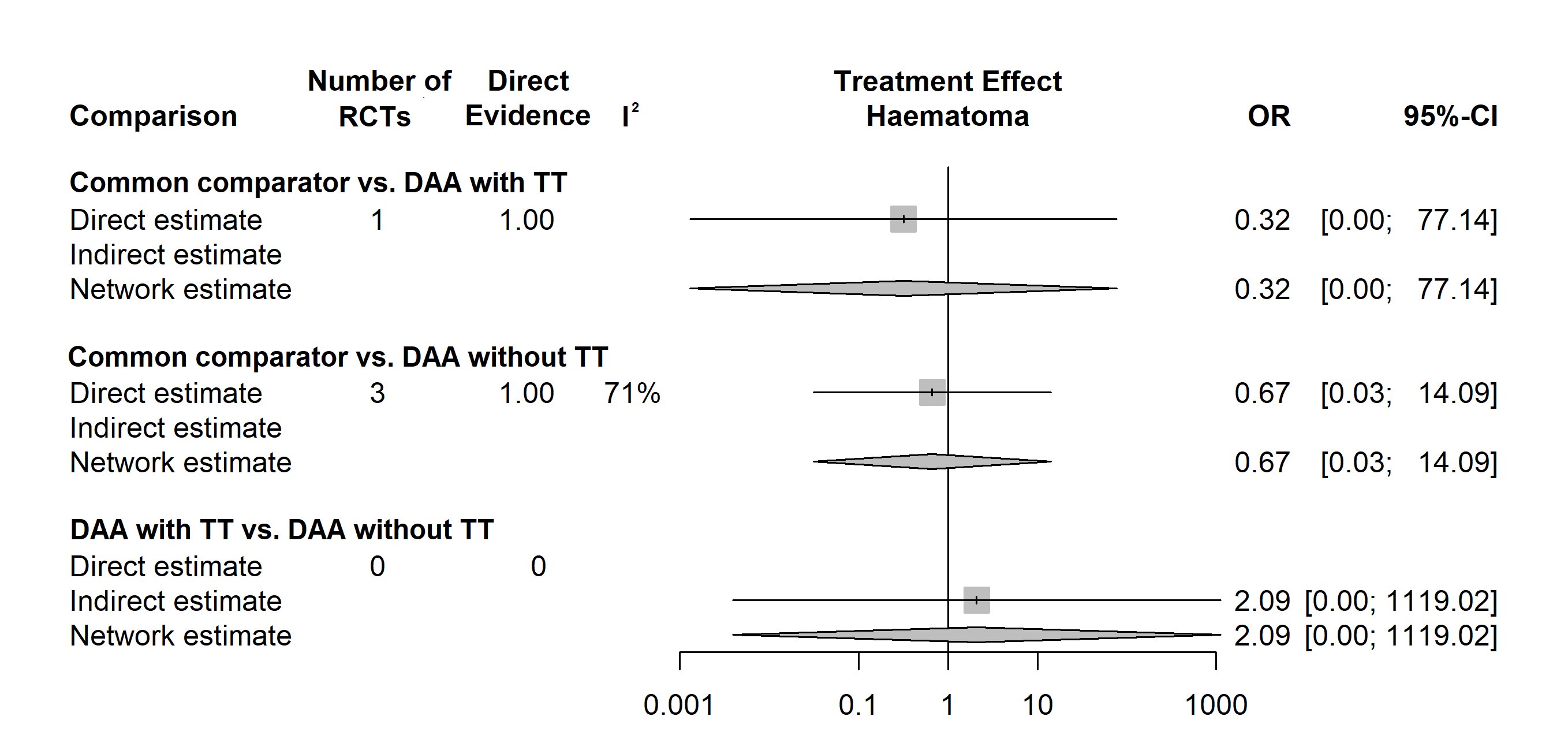

Supplement: Supplementary file 1 [file 13018_2024_4852_MOESM1_ESM.zip › Supplementary/Supplemental Figure 79 - Forest plot Sensitivity analysis Haematoma.jpg]

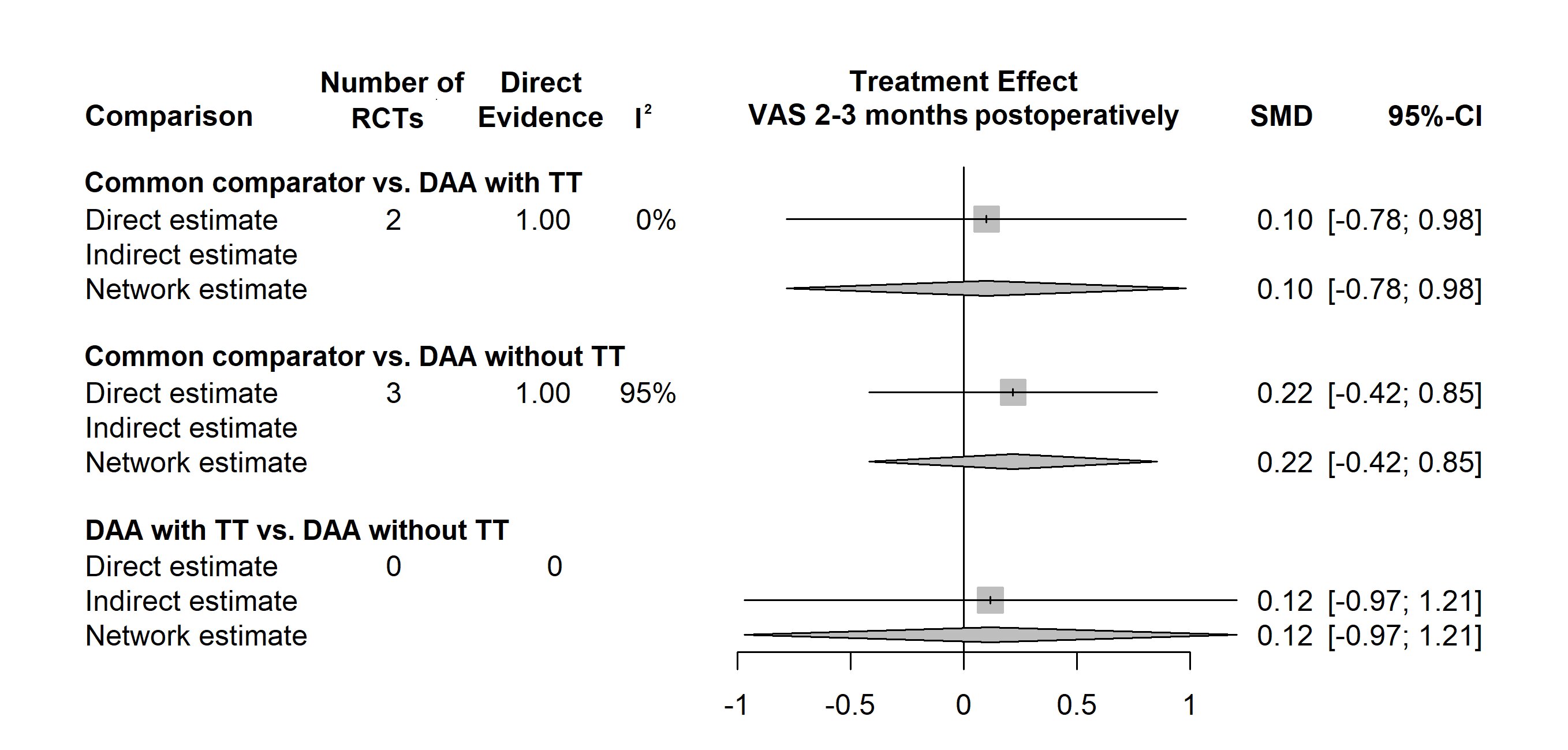

Supplement: Supplementary file 1 [file 13018_2024_4852_MOESM1_ESM.zip › Supplementary/Supplemental Figure 8 - Forest plot VAS 2-3 months.jpg]

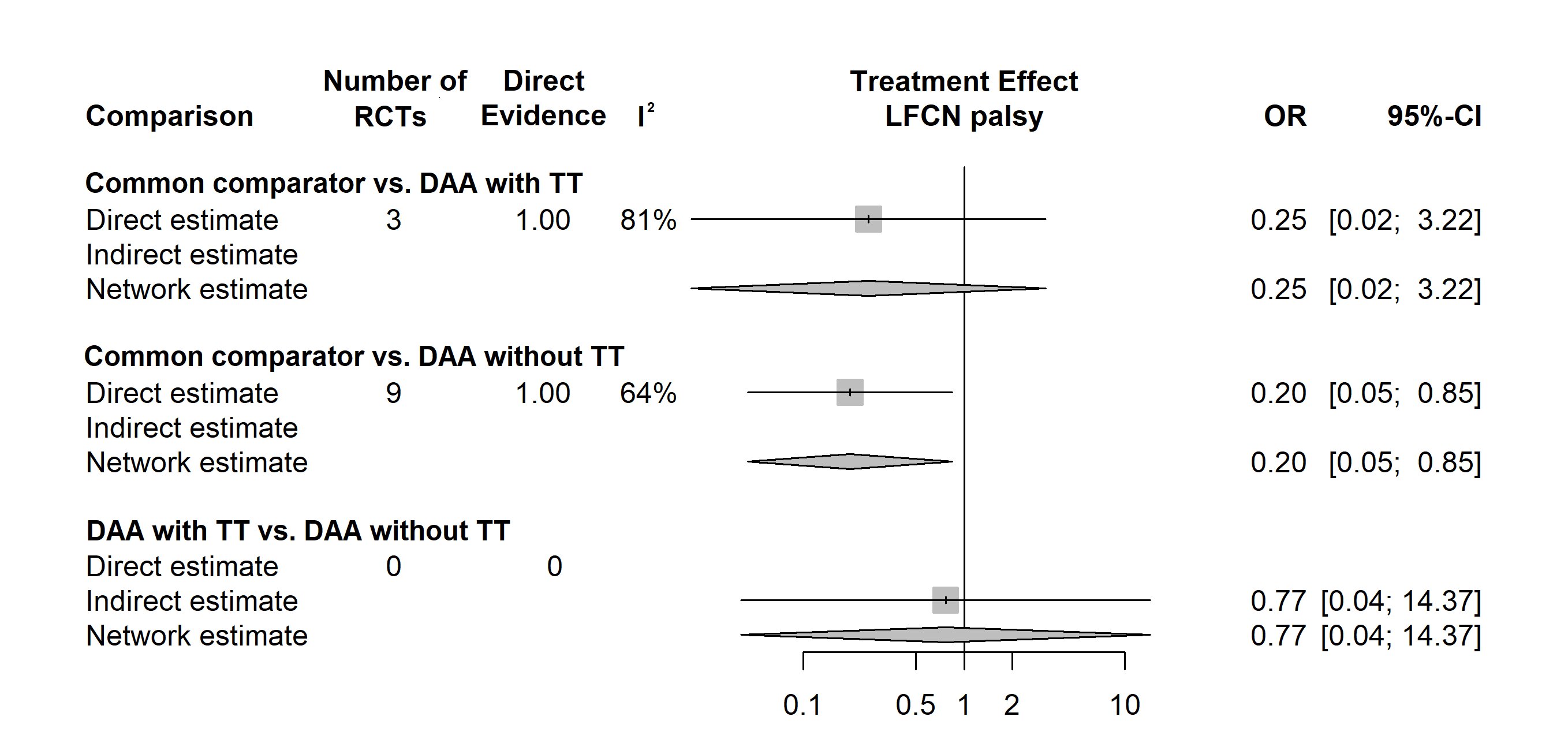

Supplement: Supplementary file 1 [file 13018_2024_4852_MOESM1_ESM.zip › Supplementary/Supplemental Figure 80 - Forest plot Sensitivity anaylisis LFCN palsy.jpg]

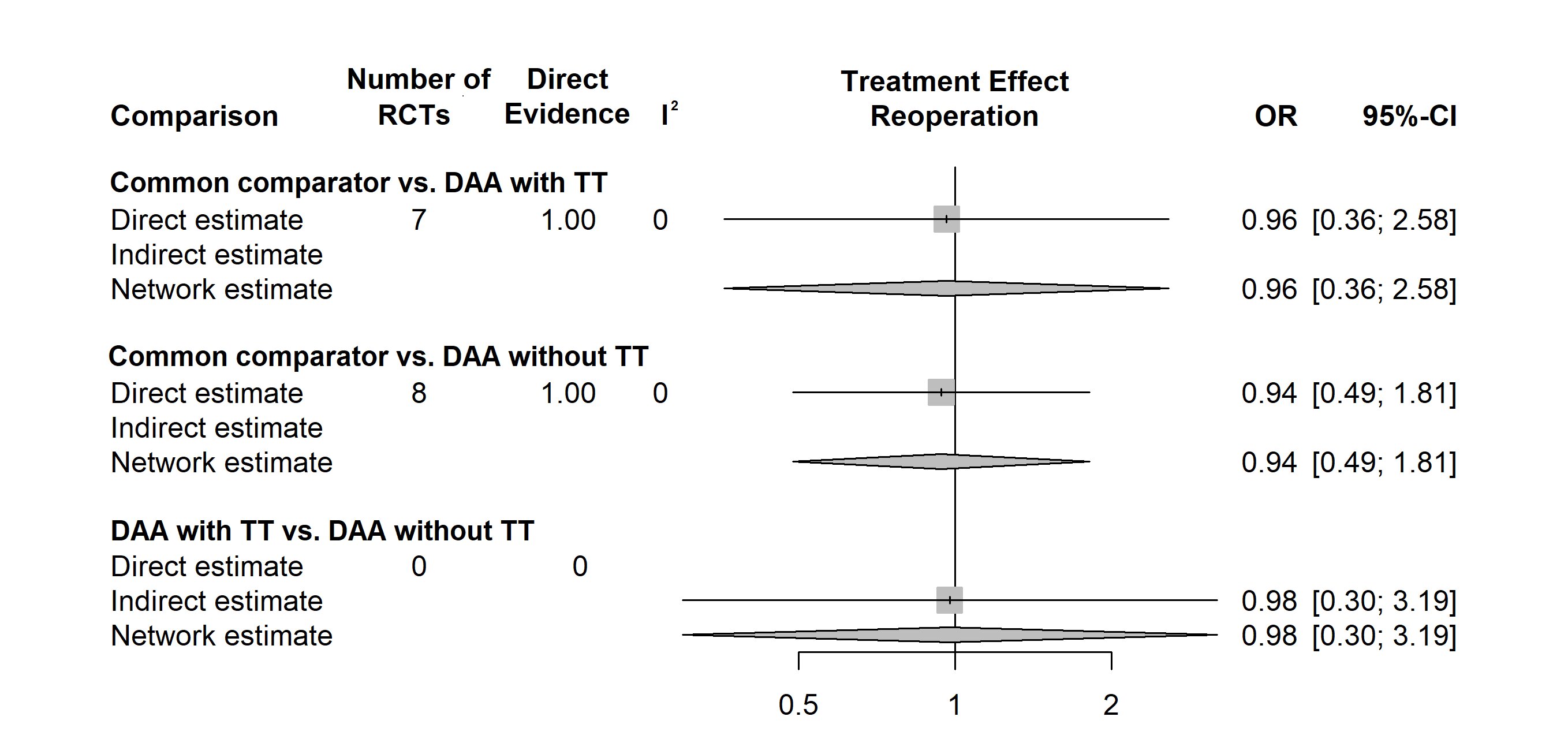

Supplement: Supplementary file 1 [file 13018_2024_4852_MOESM1_ESM.zip › Supplementary/Supplemental Figure 81 - Forest plot Sensitivity analysis Reoperation.jpg]

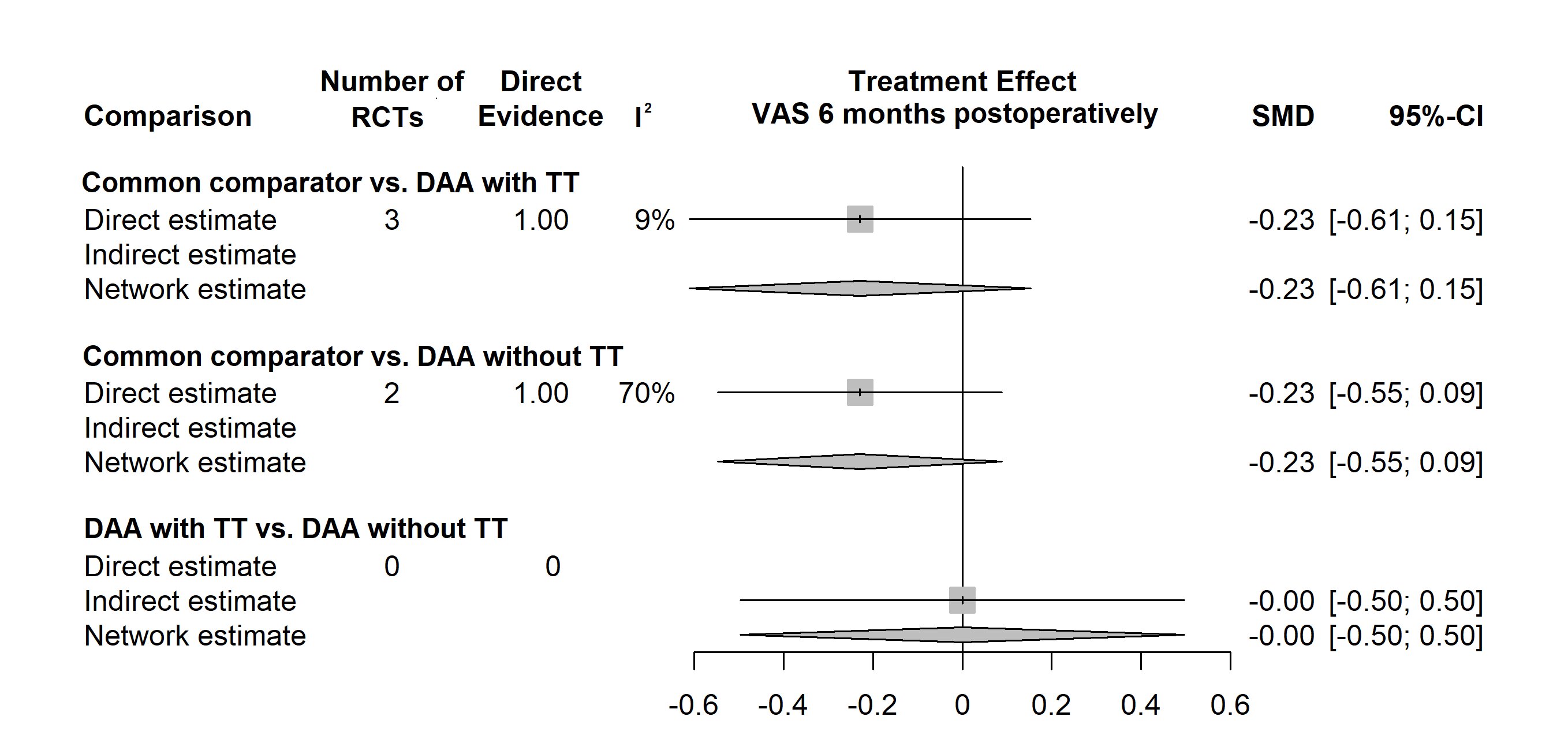

Supplement: Supplementary file 1 [file 13018_2024_4852_MOESM1_ESM.zip › Supplementary/Supplemental Figure 9 - Forest plot VAS 6 months.jpg]
